# Determinants of Human Adipose Tissue Gene Expression: Impact of Diet, Sex, Metabolic Status, and *Cis* Genetic Regulation

**DOI:** 10.1371/journal.pgen.1002959

**Published:** 2012-09-27

**Authors:** Nathalie Viguerie, Emilie Montastier, Jean-José Maoret, Balbine Roussel, Marion Combes, Carine Valle, Nathalie Villa-Vialaneix, Jason S. Iacovoni, J. Alfredo Martinez, Claus Holst, Arne Astrup, Hubert Vidal, Karine Clément, Jorg Hager, Wim H. M. Saris, Dominique Langin

**Affiliations:** 1 Inserm, UMR1048, Obesity Research Laboratory, Team 4, I2MC, Institute of Metabolic and Cardiovascular Diseases, Toulouse, France; 2 University of Toulouse, UMR1048, Paul Sabatier University, Toulouse, France; 3 Nutrition and Clinical Biochemistry Departments, Toulouse University Hospitals, Toulouse, France; 4 GeT, Genome and Transcriptome Platform, GenoToul, Toulouse, France; 5 SAMM, Statistique, Analyse, Modélisation Multidisciplinaire, University of Paris 1, Paris, France; 6 Bioinformatic Plateau, I2MC, Institute of Metabolic and Cardiovascular Diseases, Toulouse, France; 7 Department of Nutrition, Food Science, Physiology, and Toxicology, University of Navarra, Pamplona, Spain; 8 Institute of Preventive Medicine, Copenhagen University Hospitals, Copenhagen, Denmark; 9 Department of Human Nutrition, Faculty of Life Sciences, University of Copenhagen, Copenhagen, Denmark; 10 Inserm, U1060, INRA, UMR1235, Lyon University, Oullins, France; 11 Institute for Cardiometabolism and Nutrition (ICAN), Pitié-Salpêtrière Hospital, Paris, France; 12 Inserm, U872, Nutriomics, Cordelier Research Center, Paris, France; 13 University Pierre et Marie Curie-Paris 6, Paris, France; 14 CEA–Genomics Institute, Department of Human Genetics, National Genotyping Center, Evry, France; 15 Department of Human Biology, Nutrition and Toxicology Research Institute (NUTRIM), Maastricht University Medical Centre, Maastricht, The Netherlands; Georgia Institute of Technology, United States of America

## Abstract

Weight control diets favorably affect parameters of the metabolic syndrome and delay the onset of diabetic complications. The adaptations occurring in adipose tissue (AT) are likely to have a profound impact on the whole body response as AT is a key target of dietary intervention. Identification of environmental and individual factors controlling AT adaptation is therefore essential. Here, expression of 271 transcripts, selected for regulation according to obesity and weight changes, was determined in 515 individuals before, after 8-week low-calorie diet-induced weight loss, and after 26-week *ad libitum* weight maintenance diets. For 175 genes, opposite regulation was observed during calorie restriction and weight maintenance phases, independently of variations in body weight. Metabolism and immunity genes showed inverse profiles. During the dietary intervention, network-based analyses revealed strong interconnection between expression of genes involved in *de novo* lipogenesis and components of the metabolic syndrome. Sex had a marked influence on AT expression of 88 transcripts, which persisted during the entire dietary intervention and after control for fat mass. In women, the influence of body mass index on expression of a subset of genes persisted during the dietary intervention. Twenty-two genes revealed a metabolic syndrome signature common to men and women. Genetic control of AT gene expression by *cis* signals was observed for 46 genes. Dietary intervention, sex, and *cis* genetic variants independently controlled AT gene expression. These analyses help understanding the relative importance of environmental and individual factors that control the expression of human AT genes and therefore may foster strategies aimed at improving AT function in metabolic diseases.

## Author Summary

In obesity, an excess of adipose tissue is associated with dyslipidemia and diabetic complications. Gene expression is under the control of various genetic and environmental factors. As a central organ for the control of metabolic disturbances in conditions of both weight gain and loss, a comprehensive understanding of the control of adipose tissue gene expression is of paramount interest. We analyzed adipose tissue gene expression in obese individuals from the DiOGenes protocol, one of the largest dietary interventions worldwide. We found evidence for composite control of adipose tissue gene expression by nutrition, metabolic syndrome, body mass index, sex, and genotype with two main novel features. First, we observed a preeminent effect of sex on adipose tissue gene expression, which was independent of nutritional status, fat mass, and sex chromosomes. Second, the control of gene expression by *cis* genetic factors was unaffected by sex and nutritional status. Altogether, the effects of the investigated factors were most often independent of each other. Comprehension of the relative importance of environmental and individual factors that control the expression of human adipose tissue genes may help deciphering strategies aimed at controlling adipose tissue function during metabolic disorders.

## Introduction

Obesity is characterized by an excess of fat deposited in adipose tissue (AT) as triglycerides. An increase in adiposity is associated with increased risk of cardiovascular disorders and metabolic abnormalities, including hypertension, insulin resistance, type 2 diabetes, obstructive sleep apnea and cancers. Diet-induced weight loss prevents risk for type 2 diabetes and metabolic syndrome [1], [2], emphasizing the pivotal role of AT in obesity-related complications. As a key target tissue of dietary intervention and a node of integration between metabolism and immunity, adaptations occurring in AT are likely to have a profound impact on the whole body response [3], [4].

Obesity is a complex disorder with numerous contributing environmental and genetic factors. A multidisciplinary research effort involving a combination of clinical, biochemical and omics approaches appears mandatory to increase knowledge in the complexity of biological traits and processes associated with obesity [5]. Through probing of the transcriptional activity of tissues, the techniques allowing systematic analysis of AT gene expression have proved useful at identifying master genes [6] and regulatory networks involved in human obesity and related disorders [7]. Moreover, mRNAs are molecular species easily and evenly amplified. Hence, mRNA profiling remains one of the most powerful methods to comprehensively explore minute amounts of tissue. Real-time PCR, which provides great dynamic range and sensitivity, is a low throughput and time-consuming technology. DNA microarray analysis allows genome-wide profiling often applied to small subsets of samples. Combining benefits of both approaches recently became possible through the emergence of microfluidic-based technologies that use very limited sample and reagent quantities [8]. Moreover, large-scale investigation of gene expression in small AT samples obtained from microbiopsy has been impaired by poor yield of total RNA due to the richness in lipid. Optimization of AT biopsy handling and total RNA extraction is thus an essential step to profitably use AT samples for gene profiling applications.

The DiOGenes trial is one of the largest longitudinal dietary interventions worldwide consisting in an 8-week weight loss diet and a 26-week weight control phase with different dietary regimes [9], [10]. The prospective long-term, randomized, controlled study design offered a unique opportunity to apply genomics technology to dietary intervention aimed at maintaining weight loss. In this study, we applied an improved total RNA preparation from AT to the thousands of samples available during the DiOGenes study. Using a novel microfluidic technology, quantitative expression analysis of AT genes was performed in individuals from this cohort. The relationship between mRNA levels and bio-clinical and genetic data was investigated. These integrative analyses provide evidence of composite control of AT gene expression by nutrition, metabolic syndrome, body mass index (BMI), sex and genotype.

## Results

### Optimization of total RNA extraction and gene expression normalization in human adipose tissue

Despite recent development in single-step techniques dedicated to lipid-enriched samples, total RNA extraction from AT had to be improved before application to AT analysis in the DiOGenes clinical trial. Each step of total RNA extraction from small amounts of human AT samples was optimized in order to prevent the loss of precious samples (Table S1, Figure S1). In the context of large scale clinical programs, we also investigated whether long term storage of fat samples may have negative impact on total RNA integrity. AT samples frozen in liquid nitrogen can be stored at −80°C up to 3 years without affecting total RNA yield (Figure S1) or quality (data not shown). Flash freezing in liquid nitrogen before storage proves as efficient as soaking the samples in preservative solutions. This is a critical point as it allows use of fat samples for other applications than transcriptomics. Different approaches were used for real time qPCR data normalization. Use of the simple 2^−ΔCt^ method with *GUSB* as a reference transcript proved to be the best for normalization in human subcutaneous AT (Figure S2).

### DiOGenes study

The DiOGenes dietary intervention consisted of two phases [9], [10]. The first phase was an 8-week low-calorie diet (LCD) with the objective of ≥8% weight loss. In the second phase, the successful patients were randomized into one of five *ad libitum* weight maintenance diets (WMD): four diets combining high and low protein content with high and low glycemic index of carbohydrates and a control diet according to National dietary guidelines on healthy diets. Clinical investigations including subcutaneous AT microbiopsies were performed before and at the end of each phase.

Five hundred sixty eight obese individuals, age 24 to 63 (mean weight: 99.6±17.1 kg) had clinical data available and good quality AT RNA samples. Two groups of patients were defined (Figure S3). The first group, group A, included 311 obese individuals (107 men and 204 women) with gene expression data available at each clinical investigation day. The second, group B, had 204 individuals with gene expression data available at baseline and after LCD. Subjects were also categorized according to the occurrence of metabolic syndrome at baseline [11]. Group A had 125 metabolic syndrome and 186 non-metabolic syndrome individuals at baseline. Group B had 81 metabolic syndrome and 123 non-metabolic syndrome individuals at baseline. All baseline anthropometric and plasma characteristics are described in Table S2. In both men and women, blood pressure, triglycerides, HDL-cholesterol, C reactive protein, adiponectin, fasting glucose and insulin were significantly different in metabolic syndrome compared to non-metabolic syndrome individuals. In addition, women with metabolic syndrome had higher weight, BMI, fat mass and waist circumference.

### Adipose tissue gene expression is regulated during the different phases of the DiOGenes dietary program

Massive parallel reverse transcription quantitative PCR (RT-qPCR) was performed on AT from the DiOGenes study using a microfluidic qPCR device [8]. AT expression data from 271 genes of interest (Table S3) was investigated on 1341 samples from 515 subjects. The genes were selected from our previous published and unpublished DNA microarray analyses on a limited number of individuals. The choice was made using the following criteria: regulation during dietary weight loss programs [12]–[14], including the DiOGenes trial [14], and differential expression according to the presence or absence of obesity and metabolic syndrome [15], [16]. Forty percent of these genes encoded proteins involved in metabolism and 23% participated in immune response. This list encompassed 38 AT macrophage [15], [16] and 84 adipocyte markers [12], [15], i.e. genes expressed in these cell types at much higher levels than in any other AT cell type.

**Figure 1:**
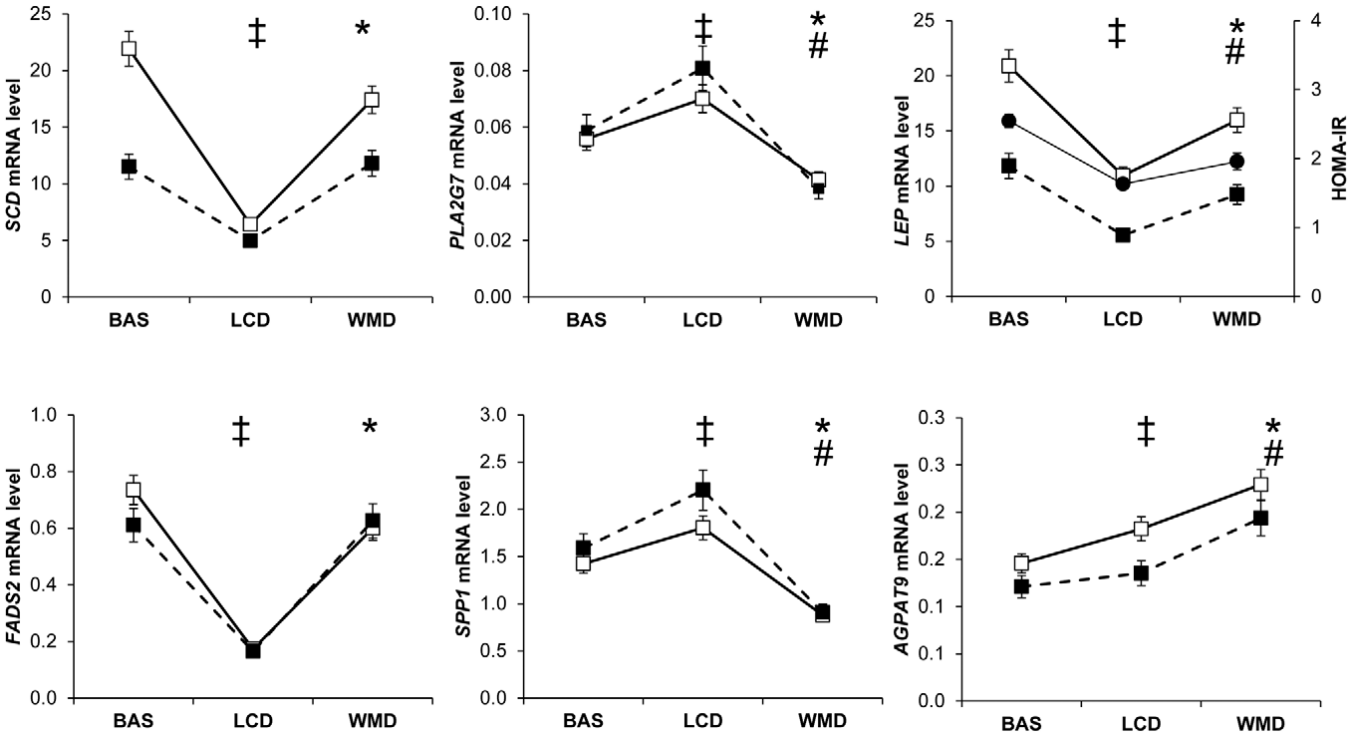
Most remarkable profiles of adipose tissue gene expression during a dietary weight loss program. Adipose tissue gene expression was assessed at baseline (BAS) and during a dietary trial including an 8 week low calorie diet (LCD) followed by a 26 week weight maintenance diet (WMD) (men, black squares *n* = 107; women, open squares *n* = 204). (‡) Comparison between BAS and end of LCD, *P*<0.05 according to linear mixed effect model ran separately for men and women. (*) Comparison between end of LCD and end of WMD, *P*<0.05 according to linear mixed effect model ran separately for men and women. (#) Comparison between BAS and end of WMD, *P*<0.05 according to linear mixed effect model ran separately for men and women. The evolution of the insulin resistance index HOMA-IR in the entire population is shown as black circles for comparison with leptin mRNA levels. Error bars represent S.E.M.

**Figure 2:**
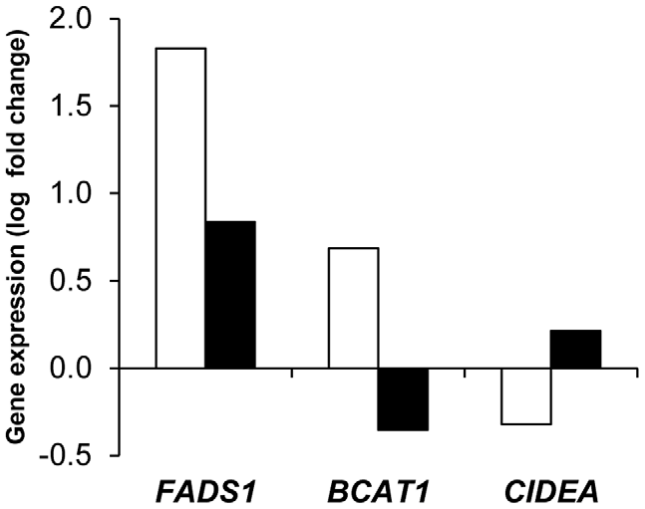
Adipose tissue markers of weight regain and weight loss during the 26-week weight maintenance diet. Changes in mRNA levels between the end of low calorie diet and the end of the 26 week weight maintenance diet were compared in women regaining (open bars *n* = 31) or losing (black bars *n* = 29) weight.

Controlling for weight variation, a majority of genes were regulated in both men and women by the dietary weight management program. The main pattern observed on 175 genes was an opposite regulation of AT gene expression between LCD and WMD phases (Figure 1, Table S4). Genes downregulated during LCD and upregulated during WMD (*n* = 158) were mostly associated with metabolic functions (*n* = 110), including 72 genes defined as adipocyte markers. The top ranking genes included *SCD*, *FADS1* and *FADS2* encoding enzymes involved in unsaturated fatty acid synthesis. An inverse trend was seen for 17 genes including 9 immunity-related genes. Four of those genes were AT macrophage markers. Most of the genes had similar expression at the end of the intervention compared to baseline. Forty three genes showed variations in expression at the end of WMD compared to baseline (Table S5). The majority showed decreased expression compared to baseline. *LEP* showed a downregulation during calorie restriction that persisted until the end of the intervention. As shown in Figure 1, this pattern is superimposable with the evolution of HOMA-IR, an index of insulin resistance. The macronutrient composition of the diet during the WMD phase had no effect on AT gene expression. We also looked at genes related to weight changes during the *ad libitum* WMD by comparing changes in mRNA levels between the end of LCD and the end of WMD in women who lost and those who regained at least 50% of the weight lost during calorie restriction. The changes in mRNA levels of 16 genes differed between the two groups of women (Table S6). *CIDEA* which is involved in fat cell lipid droplet metabolism was the best marker for weight loss (Figure 2). *FADS1*, encoding a fatty acid desaturase, and *BCAT1*, encoding a branched-chain amino acid aminotransferase, were the best markers for weight regain.

### Sex, metabolic syndrome, and obesity impact adipose tissue gene expression

**Figure 3:**
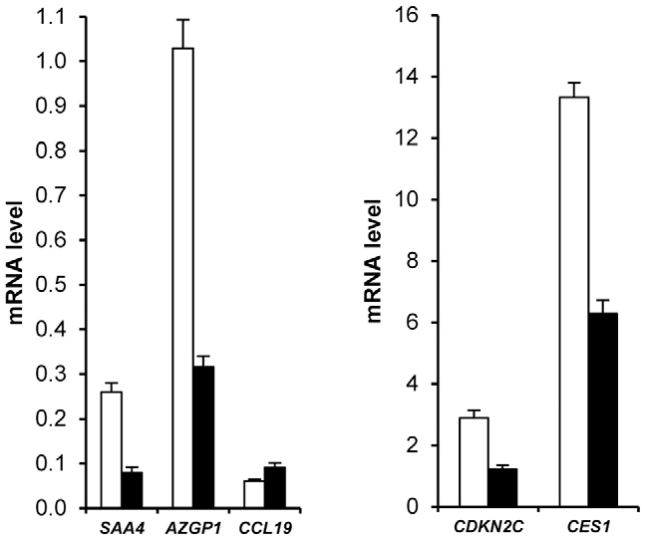
Sexual dimorphism of adipose tissue gene expression. Men, open bars (*n* = 180). Women, black bars (*n* = 323). Most representative adipose tissue genes are shown (See Table S7). Error bars represent S.E.M.

Principal component analysis of gene expression data from group A and group B subjects at baseline indicated that the major component explaining AT gene expression data distribution was sex. Figure S4a depicts partial least square-discriminant analysis of AT genes with sex specificity. To list the AT genes with sex-biased expression, a mixture model controlling for centre was first built with data from group A. One hundred and eighty six genes exhibited sex specificity. The same model was then run with data from group B, giving a list of 158 genes. Sex specificity for 109 transcripts persisted during the dietary intervention (Table S7). Higher expression in female AT was found for all genes except for *CCL19*, which showed higher expression in male AT (Figure 3). Fat mass being higher in women than in men could possibly explain this marked sexual dimorphism. However, 88 genes remained different when controlling for fat mass. Only 5 genes were located on sex chromosomes (Table S7). *SAA4*, *AZGP1*, *CDKN2C* and *CES1* were the highest ranked genes with a more than two-fold higher expression level in female than in male AT (Figure 3).

**Figure 4:**
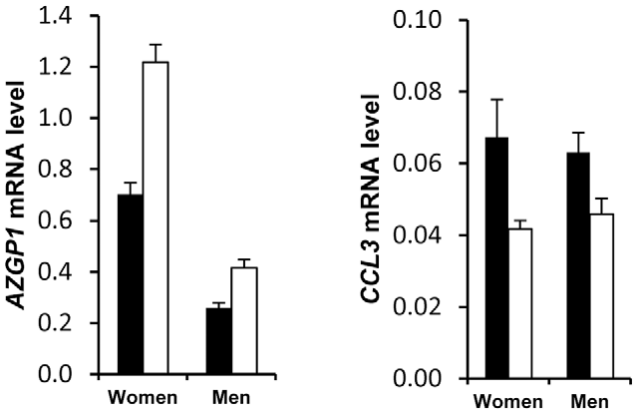
Differential adipose tissue gene expression in obese individuals according to the presence of metabolic syndrome. Adipose tissue gene expression in obese individuals with (black bars *n* = 206) and without (open bars *n* = 309) the metabolic syndrome (see Table S8). Error bars represent S.E.M.

Exploratory analysis of AT gene expression also indicated a discriminatory effect according to the presence or absence of metabolic syndrome (Figure S4b). Because the clinical presentation of metabolic syndrome is different in men and women and might be at least in part originating in the AT [16], [17], we separately analysed the 2 populations to assess the molecular characteristics of AT from patients with metabolic syndrome. A metabolic syndrome signature was found for 22 genes (Table S8). *CCL3* and *AZGP1* showed two-fold higher and lower expression, respectively, in women with metabolic syndrome compared to women without metabolic syndrome (Figure 4). The difference, albeit less pronounced, was also present in men.

To assess the contribution of obesity to AT gene expression, the impact of BMI was studied in men and women separately at baseline and along the dietary intervention. In women, 51 genes showed significant BMI dependency that persisted during the whole dietary intervention (Table S9). In men, a single gene, *AZGP1*, was dependent on BMI at each time point of the intervention (data not shown).

### Human adipose tissue functional interconnections

**Figure 5:**
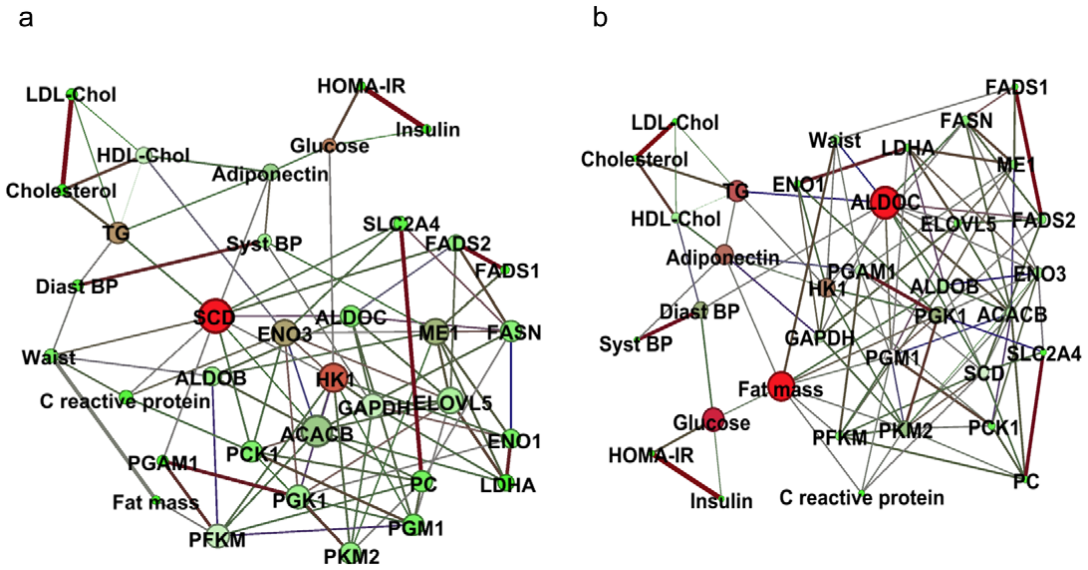
Dependency networks and dietary intervention. Two dependency networks were constructed from selected bio-clinical data and gene expression from the lipogenic module. Each node is a gene or a bio-clinical parameter. Node degree is indicated with size. Node color indicate betweenness centrality measure that counts how often a node appears on shortest paths between two other nodes in the network; red nodes are variables with high betweenness and green nodes are variables with low betweenness. Betweenness centrality indicates nodes that are the most likely to disconnect network if removed. The variables are connected by an edge only if their partial correlation is significantly nonzero. Edge thickness is proportional to the strength of correlation. Edge color indicates correlation from the most positive (red) to the negative (green). (a) Dependency network of changes from baseline to the end of the low calorie diet. (b) Dependency network of changes from baseline to the end of the weight maintenance diet.

To elucidate the relationship between AT gene expression and related phenotypes at a greater depth, we used a co-correlation network-based approach. Interactions between the two matching bio-clinical and AT gene expression data sets were modeled using partial correlations. By eliminating over-estimation of co-correlations due to correlation with a third variable, partial correlations measure direct correlation between two variables with control for confounding variables. First, in addition to bio-clinical parameters (Table S2), we selected 38 metabolism and immunity related genes that showed regulation in the present analysis (Table S10). Since sex appeared to control AT gene expression variance more than other parameters, we used the network approach on the 180 men and 335 women separately. Figure S5 displays the regulatory networks in men and women. Edges represent direct and strong correlations and thickness connection strength between variables. Each node represents a variable. Node degree refers to the number of edges attached to the node. High degree indicates hubs which are the most connected variables. Betweenness centrality quantifies the importance of a variable within network. Nodes with highest betweenness centrality are those providing the strongest network connection and show key variables in the network. The topology of the male and female networks was similar with 75% of edges in common between men and women. A majority of highly connected co-expression networks consisted of the same clinical parameters and genes that clustered together in both men and women. Several macrophage markers (Table S3) showed strong connection. A module consisting of the same group of correlated genes in both the male and female networks encompassed genes involved in *de novo* lipogenesis such as *FASN, SCD, FADS1, FADS2* and *ELOVL5*. In men and women, *CIDEA* and *AZGP1*, two cachexia markers, were connected. Next, the network-based approach was used on 204 women from group A (Table S2) to expand the analyses to functional interconnections during the dietary intervention. The list of genes consisted of the lipogenesis module observed at baseline (Figure S5) extended to related glycolysis and glucose metabolism genes (Table S11). Networks were built during LCD (Figure 5a) and from baseline to the end of the WMD (Figure 5b).The two data-driven dependency networks in AT showed 62% of shared edges. *HK1* appeared as an important hub, with 10 connections, including another hub, *SCD*, *ACACB, ELOVL5* and fasting glucose (Figure 5a). Notably, two components of the metabolic syndrome, waist and triglycerides, were highly connected to the most important node, *SCD*, which was also highly connected to glycolytic genes (*SLC2A4* and *PCK1*) during LCD. From baseline to the end of the WMD (Figure 5b), fat mass, waist and triglycerides were associated with the network key node *ALDOC*, which was connected to several lipogenic genes (*FASN, ACACB, SCD, FADS2* and *ELOVL5*).

### Human adipose tissue gene expression is under genetic control by *cis* signals

**Figure 6:**
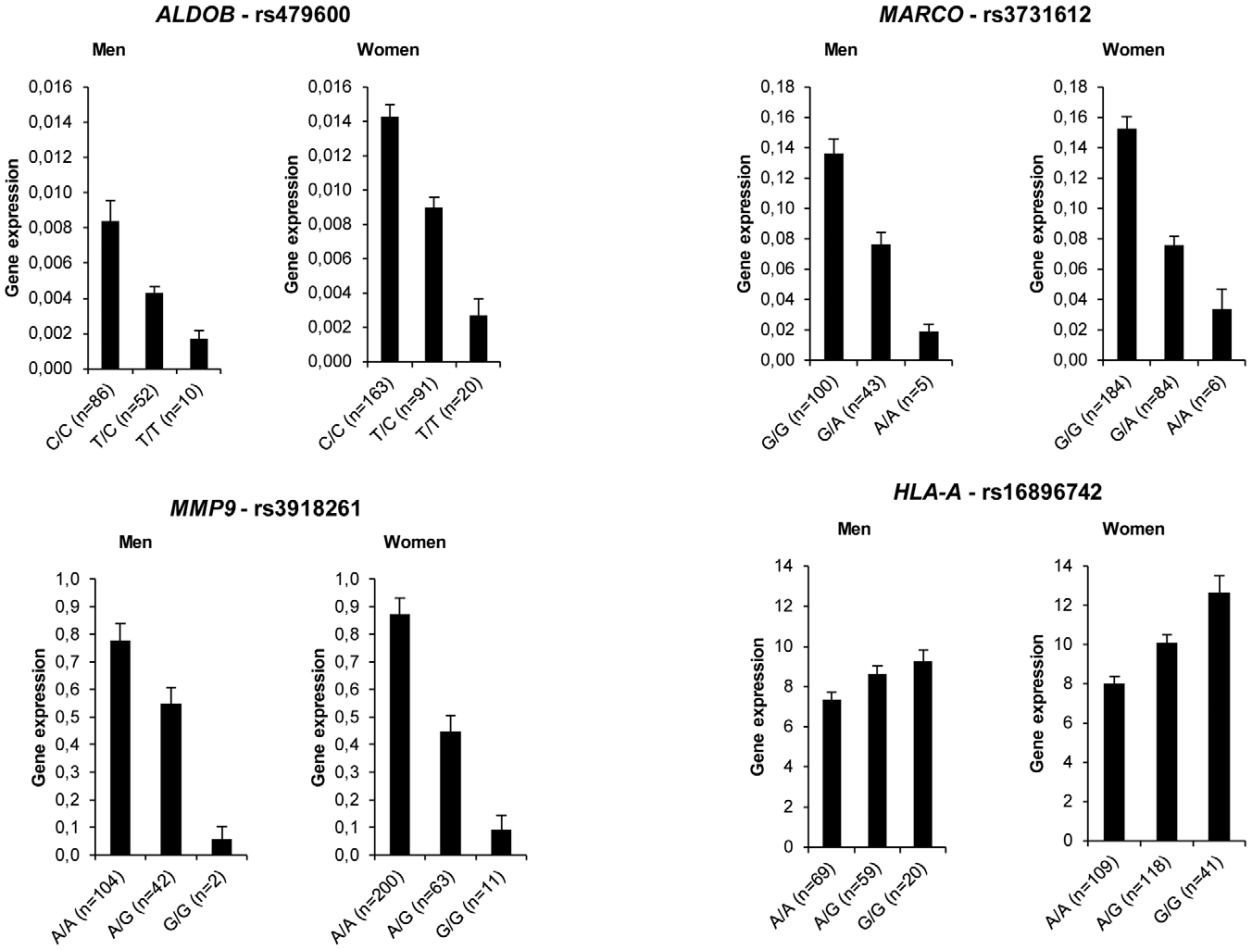
Adipose tissue gene expression according to genotype distribution of *cis* signals. Most representative adipose tissue genes are shown (see Table S12). Error bars represent S.E.M.

We identified 2953 single nucleotide polymorphisms (SNPs) which were in the close proximity of 252 genes. At baseline, 118 SNPs representing 46 genes showed association with AT gene expression (Table S12). The strongest associations (*P*<10^−10^) were found for *ALDOB*, *MARCO*, *MMP9* and *HLA-A* (Figure 6). Four SNPs located in the intronic regions of *MARCO*, which encodes an AT macrophage-specific marker regulated by obesity [15] and dietary intervention (Table S5), showed associations with *P*<10^−20^. A moderate effect of sex and BMI was observed for 3 and 13 SNPs, respectively. However, these effects were not consistent among SNPs with significant associations with AT gene expression in the corresponding genes. The majority of the associations observed at baseline remained significant when expression after LCD and WMD were considered (Table S13). Of note, no SNP showed association with diet-induced variations in mRNA levels (*P*>0.5).

## Discussion

Carefully monitored weight-control diets favorably affect parameters of the metabolic syndrome and delay the onset of diabetic complications [1]. AT is a key player in the homeostatic control of whole body metabolism. Besides the more recent DNA methylome and microRNA arrays, gene expression profiling is one of the most comprehensive omics technologies, which permits parallel quantitative measurements of a large number of targets. However, a minimal amount of high quality total RNA is required. To study large intervention programs, we optimized the entire process, from needle biopsy of human subcutaneous AT to long term storage of samples. A single needle biopsy allows fast and painless AT sampling that may be easier to perform than blood sampling in morbidly obese subjects. Biopsies of about 200 mg are easily obtained in large scale intervention studies and can also be obtained from lean individuals. From this amount of tissue up to 34,100 different transcripts can be quantified using the Fluidigm Biomark Dynamic Array technology. This high-throughput technology has been successfully applied to many biological fields [18]–[20]. It allows reduction in cost and time, and improvement of accuracy, throughput and performance compared with conventional instruments. We show here its potencies to study AT expression of multiple genes in large-scale population-based interventions. Normalization is an essential step to correct for systematic bias in transcriptomic data. In microfluidic RT-qPCR assays, systematic errors due to sampling, reverse transcription and preamplification steps as well as set and plate spreading must be eliminated. Numerous different methods can be used for data normalization, including those used for transcriptome analyses [21]. When analyzing real-time PCR data, the most widely used is the 2^−ΔCt^ method using a reference gene [22]. The ideal reference, also referred to as a house-keeping gene, should be constantly transcribed in all cell types and tissues regardless of internal and external influences. However, the expression of house-keeping genes may vary considerably. *GAPDH* is one of the most commonly used house-keeping genes. However, *GAPDH* expression was shown to be regulated during dietary intervention in this study and previous work [12]. The ribosomal *18S* is another common reference gene. However, because of its very high expression level, this transcript shows such a strong expression after the preamplification step that it overflows the detection system. Here, the use of *GUSB* as a reference gene in the easy-to-use 2^−ΔCt^ method proved to deliver the best normalization for human subcutaneous AT.

AT gene expression profiling combined with clinical investigations has opened a novel approach to decipher physiological and pathophysiological processes [5]. Most previous studies have aimed at comparing obese and lean individuals and observing the effects of altered body weight during dynamic weight change. The highly clinically relevant weight stabilization phase has rarely been studied. We have previously investigated AT gene expression during multiple phase dietary interventions on a limited number of individuals [12]. The DiOGenes intervention study was designed to investigate diet-induced changes on a much larger scale [10]. This trial focused on identifying key dietary and genetic factors as a basis for predicting whether individuals may reach and maintain healthy weight. The number of participants and the complete bioclinical characterization combined with AT biopsy offered a unique opportunity to study the interactions between sex, metabolic status, dietary phases and genetic factors. Unexpectedly, a striking sex effect on AT gene expression that persisted during the dietary intervention for most of the sex-specific genes was found. As a prototypical example, it has repeatedly been shown that leptin AT mRNA level is higher in females than in males even after correction for the degree of body fat mass [23]. Similar sexual dimorphism has previously been reported for 45 of the genes described in the present study in mouse AT [24]. Higher expression in female than in male AT was found for all but 1 gene. However, to ascertain that the majority of human AT genes showing sex differences have female-biased expression, a systematic analysis would be required. Higher fat mass in women than in men could possibly explain this marked sexual dimorphism. However, 80% of the genes remained different when controlling for fat mass. Indeed, *SAA4* and *CDKN2C* AT expression are increased and decreased, respectively, in morbidly obese subjects whereas the two genes show higher expression in women than men [25], [26]. The contribution of gonadal hormones and sex chromosomes has been investigated in mouse models. Sexual steroids play a strong role in sex-biased gene expression in various tissues with minor sex differences explained by direct effects of the sex chromosome in liver genes [27]. Here, less than 5% of the human AT sex-related genes were located on sex chromosomes. The large number of subjects with a wide range of adiposity allowed testing the effect of BMI on AT gene expression at baseline and during the dietary intervention. In women, 51 genes show BMI dependency which persists at each time point of the dietary intervention indicating adiposity-dependent control of gene expression that is not influenced by diet-induced changes in weight. *AZGP1* was the only common gene to men and women and among the top ranking less expressed genes in the morbidly obese patients (BMI>40). It was recently shown as down regulated with fat mass expansion in obesity in both visceral and subcutaneous fat with positive association with adiponectin [28]. Of note, the network approach also showed such connection in the male network. The lower number of significant genes in men may be related to real sex differences but may also be due to the lower number of men in the cohort. A metabolic syndrome signature was also found in AT. As a top ranking gene, *CCL3* encodes macrophage inflammatory protein 1α, a CC chemokine involved in the interactions between immune cells and regulated by insulin resistance in AT [29]. *AZGP1* appears as a marker of sexual dimorphism, obesity and metabolic syndrome encodes an adipokine with putative antidiabetic properties [30].

When looking along the DiOGenes dietary program, the main pattern was an opposite regulation of AT gene expression between LCD and WMD phases. Genes downregulated during LCD and upregulated during WMD were mostly adipocyte genes associated with metabolic functions [12]. The top ranking genes encoded enzymes involved in fatty acid desaturation [31]. An inverse trend was seen for immunity-related genes. As a result of the opposite regulation between LCD and WMD, most of the genes had similar expression at the end of the intervention compared to baseline. However, a subset of genes showed downregulation at the end of the dietary intervention. The list included several genes previously characterized as human AT macrophage-specific markers (*CD68*, *CD163*, *CD209*, *IL10*, *LIPA*, *MARCO*, *MS4A4A, PLA2G7, SPP1*) [12], [15]. This coordinated downregulation most likely reflects a decrease in AT macrophage number as observed in a 6-month weight reducing intervention [32]. Leptin mRNA levels were also lower at the end of the dietary intervention. The superimposition of *LEP* and HOMA-IR data lend support to our hypothesis that variation in *LEP* expression contributes to the improvement in insulin sensitivity observed during diet-induced weight loss [3].

Genes related to weight changes during WMD include *AGPAT9*, presumably involved in the biosynthesis of triacylglycerol and phospholipid, *ALOX12*, encoding the arachidonate12-lipoxygenase involved in production of inflammatory and adipogenesis mediators, and the proangiogenic *VEGFA*, which all showed robust overexpression in individuals who regained weight. *PKM2*, *AP2M1, ACTR3* and *CES1* had been shown in microarray experiments to have higher expression in individuals who failed to control their weight [14]. *FADS1*, encoding a fatty acid desaturase, and *BCAT1*, encoding a branched-chain amino acid aminotransferase, were the best markers for weight regain. Interestingly, branched-chain amino acid catabolism is down-regulated in obese individuals [33]. *CIDEA*, which plays a critical role in fat cell lipid droplet metabolism [34], showed decreased expression in individuals continuing to lose weight after LCD, supporting a role for the encoded protein in the adaptation of subcutaneous AT to body weight changes as over-feeding-induced weight gain induces its expression [35]. The macronutrient composition of the diet (i.e., protein content and glycemic index) during the WMD phase had no effect on AT gene expression in agreement with previous data on energy-restricted diets differing in fat and carbohydrate content [13]. Therefore, during dietary weight management programs, energy balance and fat mass variations rather than the composition of the diet is a determinant of AT gene expression.

During LCD, network analysis of gene expression and clinical parameters showed that the top associations function as part of a major hub gene, the stearoyl CoA desaturase *SCD*, which is highly connected to components of the metabolic syndrome and the gene encoding a glycolytic enzyme, hexokinase *HK1*, connected to glycemia. These genes are targets of ChREBP, a transcription factor involved in glucose-mediated control of *de novo* lipogenesis gene expression [36], [37]. These connections suggest that ChREBP target genes are regulated during LCD. This transcription factor was not studied here. The link between ChREBP and metabolic improvements along the dietary intervention requires further investigation [38]. Along the dietary intervention, clinical data and gene co-expression network analysis also revealed *ALDOC*, an aldolase involved in glycolysis, and fat mass as key nodes. Both hubs were connected to components of the metabolic syndrome. The *ALDOC*-centered module included key genes for *de novo* lipogenesis, illustrating the common transcriptional control of glycolysis and fatty acid synthesis [37]. The fat mass centered module was composed of glycolytic genes, indicating a direct link between change in fat mass and aerobic glycolysis, which seems to be related to the connection between *de novo* lipogenesis gene expression and metabolic features [39].

Human AT gene expression is under strong genetic control [40]. Recent genome-wide gene expression and genotyping analysis identified 10,000 *cis* SNPs associated to gene expression in subcutaneous AT [41]. The number of *cis* expression SNPs (eSNPs) was much higher than the number of *trans* eSNPs. In the present study, more than 80% of the genes with eSNPs had not previously been reported [41]. This high level of detection was related to several factors. First, we selected SNPs located in the immediate vicinity of the genes that allow capture of significant associations with our sample size [42]. Second, we investigated a carefully selected population enrolled in a multicentric dietary intervention [9], [10]. Thereby, we could control for biological and non-biological confounders such as center, sex, fat mass and diet. Highly significant associations were found for *MARCO* and *MMP9*. *MARCO* encodes a class A scavenger receptor shown to be specific of AT macrophages compared to other human AT cell types [15]. AT macrophages also specifically produce metalloproteinase 9, a key enzyme involved in remodeling processes [43]. Of 46 genes with eSNPs, 19 were directly related to immunity and inflammation and were highly expressed in human AT, in agreement with the existence of an AT macrophage gene network module with tight *cis* genetic control [40]. Strikingly, the eSNPs identified here were not influenced by sex and diet-induced changes in AT gene expression. We found no evidence of association between *cis* SNPs and variations in mRNA levels during the dietary protocol, suggesting that *cis* genetic control operates at baseline and is preserved during the dietary intervention but does not influence the response to the diet.

**Figure 7:**
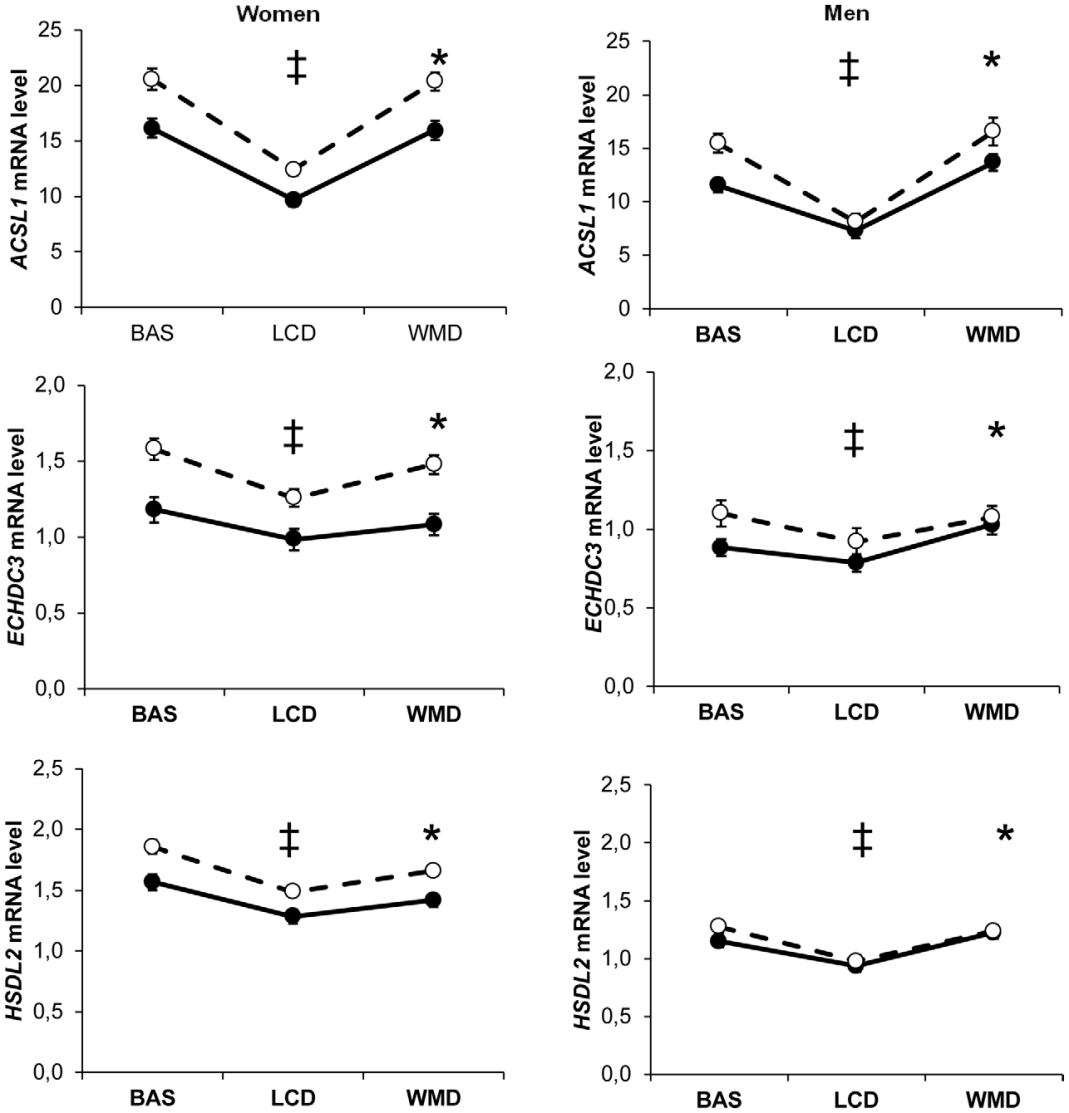
Most representative adipose tissue gene expression profiles according to dietary intervention, sex, and metabolic syndrome. *ACSL1, ECHDC3* and *HSDL2* adipose tissue gene expression according to dietary intervention (BAS, baseline; LCD, low calorie diet; WMD, weight maintenance diet), sex and metabolic syndrome (presence, black circles; absence, open circles) in 311 obese individuals. (‡) Comparison between BAS and end of LCD, *P*<0.05 according to linear mixed effect model ran separately for men and women. (*) Comparison between end of LCD and end of WMD, *P*<0.05 according to linear mixed effect model ran separately for men and women. (#) Comparison between BAS and end of WMD, *P*<0.05 according to linear mixed effect model ran separately for men and women. Error bars represent S.E.M.

As prototypical examples, *ACSL1, ECHDC3* and *HSDL2* mRNA levels were influenced by all the investigated factors (Figure 7). SNPs did show associations with AT mRNA levels as transcript abundance varied during the dietary intervention. It also differed according to sex and metabolic syndrome. Dietary intervention did not alter the sexual dimorphism in gene expression. ACSL1, which catalyzes the conversion of long chain fatty acids into acyl-CoAs, is the most abundant ACSL isoform expressed in AT. AT-specific ablation of *Acsl1* in mice shows that the enzyme plays a crucial role in directing acyl-CoAs towards β-oxidation in fat cells [44]. Here, *ACSL1* gene expression was lower in individuals with metabolic syndrome. An association between *ACSL1* gene polymorphisms and the metabolic syndrome has recently been reported [45]. These data suggest that impaired adipocyte fatty acid oxidation due to *ACSL1* defect may constitute a feature of the metabolic syndrome.

The present data provide evidence for control of AT gene expression by nutrition, sex, metabolic status and genotype. A main feature was a major effect of sex, which was independent of sex chromosomes, fat mass and dietary intervention. Another characteristic was that the control of gene expression by genetic elements appeared unaffected by nutritional status. Altogether, the effects of the investigated factors were most often independent of each other. Understanding the relative importance of environmental and individual factors that control the expression of human AT genes may help in deciphering strategies aimed at improving AT function in metabolic diseases.

## Methods

### Human subcutaneous adipose tissue sampling

Fat samples used for the optimization of total RNA extraction were obtained using abdominal dermolipectomy from the plastic surgery department of the Toulouse University Hospitals. The patients were not included in a weight reduction program.

DiOGenes was registered in ClinicalTrials.gov (NCT00390637). Briefly, 932 overweight and obese adults in 8 European centers participated in a dietary program with a 8-week 3.3 MJ/day LCD (Modifast, Nutrition et Santé, France). Subjects achieving 8% of initial body weight loss were randomized to a 6-month *ad libitum* WMD consisting in one of four low-fat diets that differed in glycemic index and protein content, or a control diet as described in [9]. Abdominal subcutaneous AT biopsies from the DiOGenes protocol were obtained by needle aspiration under local anesthesia after an overnight fast at baseline, at the end of LCD and at the end of WMD [14]. All clinical investigations were performed according to standard operating procedures. Analysis of blood samples was performed at the Department of Clinical Biochemistry, Gentofte University Hospital, Denmark as described in [9].

The study was performed according to the latest version of the Declaration of Helsinki and the Current International Conference on Harmonization (ICH) guidelines. All subjects gave verbal and written informed consent. Applications were submitted to the regional Ethics Committees from the participating centres and the study was not undertaken without a positive statement from the committee regarding the study.

### Optimization of total RNA extraction

Five single-step methods for total RNA extraction were evaluated on 6 human subcutaneous AT samples (Table S1). Among the 5 extraction methods, 2 yielded very few or partly degraded total RNA and one low purity RNA. The QIAGEN methods (RNeasy Mini and RNeasy Lipid Tissue Mini) yielded sufficient amount of good quality total RNA that appeared to be free of genomic DNA contamination. Based on the QIAGEN RNeasy Lipid Tissue Mini Kit, an in-house optimized total RNA preparation protocol that uses chloroform delipidation and phenol/guanidine isothiocyanate-based (QIAzol) extraction, silica-gel membrane purification and microspin technology was set up (see below). This adapted protocol provided a higher mean total RNA supply with more consistent yield than other methods.

### Adipose tissue sampling and storage conditions

Human AT samples of weights ranging from 0.04 g to 1.5 g were collected, flash frozen in liquid nitrogen and stored at −80°C. Figure S1a was drawn from 84 AT samples. It shows a positive correlation between the amount of total RNA extracted and the weight of the fat biopsies up to 0.2 g. Above 0.5 g of fat, the amount of total RNA per g of tissue becomes more variable. The data reveal that 0.3 to 0.5 g of fat are enough for substantial total RNA recovery. Such an amount may yield a minimum of 5 µg of total RNA which is sufficient for both microarray application and RT-qPCR. Besides sample preparation, storage conditions are a major concern because of the instability of mRNA due to contaminating RNases. In order to prevent total RNA degradation, commercial RNA stabilization reagents are available. Figure S1b shows adipose tissue total RNA yield and quality using alternative protocols. Samples of about 0.5 g of human fat tissue were collected and stored at −80°C following 5 different protocols: 1) immediate storage of the freshly cleaned fat sample at −80°C 2) flash-frozen in liquid nitrogen and stored at −80°C 3) stored overnight in RNAlater RNA Stabilization Reagent (QIAGEN) at 2–8°C, then removed from the RNAlater and stored at −80°C 4) freshly cleaned fat sample stored at −80°C in QIAzol Lysis Reagent (QIAGEN) 5) homogenized in QIAzol Lysis Reagent with ultra-turax homogenizer and stored at −80°C. These samples were extracted after short term, 1 month, and after long term, 1 year, storage. The 5 protocols gave similar total RNA yield and quality.

### In-house total RNA extraction protocol

The frozen AT sample was homogenized in QIAzol (QIAGEN) (2.5 ml of QIAzol for 500 mg of tissue, 5 ml for 1 g of tissue, 1 ml for ≤200 mg of tissue) using a rotor-stator homogenizer until homogeneity (20–40 s; longer time may lead to overheating) then incubated at room temperature for 5 min. Two hundred µl of chloroform was added for 1 ml of QIAzol (otherwise the volume of chloroform was adjusted to QIAzol volume with a 1∶5 ratio) and vigorously shaked for 15 s using a vortex then incubated at room temperature for 3 min. After centrifugation at 4000 rpm for 15 min at 4°C the upper aqueous phase was transferred to a new tube. One volume of 70% ethanol was added and mixed by vortexing. Seven hundred µl of this sample was pipetted onto an RNeasy Mini Spin Column (QIAGEN) in a 2 ml tube and centrifuged at ≥10,000 rpm for 15 s at 25°C. Flow-through was discarded. This step was repeated using the reminder of the sample. Seven hundred µl of Buffer RW1 (QIAGEN) was added to the column and centrifuged at ≥10,000 rpm for 15 s at 25°C. Flow-through was discarded. The column was transferred into a new 2 ml tube. Five hundred µl of Buffer RPE (QIAGEN) was added to the column and centrifuged at ≥10,000 rpm for 15 s at 25°C. Flow-through was discarded. Another 500 µl of Buffer RPE was added to the column and centrifuged at ≥10,000 rpm for 2 min at 25°C. The column was transferred into a new 2 ml tube and centrifuged at ≥10,000 rpm for 1 min at 25°C. To elute, the RNeasy Mini Spin Column was transferred to a 1.5 ml tube and 30 µl of preheated RNAase-free water at 50°C directly pipetted onto the column then centrifuged at ≥10,000 rpm for 1 min at 25°C.

### Total RNA quality check

For the optimization of total RNA extraction from AT samples (Table S1), total RNA quality was checked using ethidium bromide-stained agarose gels. Concentration was determined using a Nanodrop spectrophotometer, Illkirch, France). For AT biopsies from the dietary program, total RNA concentration and quality were estimated by capillary electrophoresis using the Experion analyzer (BioRad, Marnes-la-Coquette, France). The amount of total RNA from the DiOGenes study was 25.3±9.3 µg/g of AT (*n* = 1363) validating the RNA extraction and purification method in a large multicenter study. Total RNA was of good quality and free of genomic DNA.

### RT–quantitative PCR using BioMark System

#### Specific target amplification

cDNA was prepared as previously described [46] from 200 ng or 500 ng of total RNA, depending on RNA concentration and diluted in water to 5 ng/µl (RNA-equivalent). Because 286 transcripts were to be quantified using 96.96 Dynamic Arrays (Fluidigm), each diluted cDNA sample was split into 4 parts. The first part was used to check 18S expression level homogeneity using ABI Prism 7900HT (Applied Biosystems) to ensure good quality reverse transcription step. The 3 other parts were dedicated to target amplification for Biomark Dynamic Arrays (Fluidigm). Inventoried TaqMan assays (Applied Biosystems) were pooled into 3 separate sets (A, B, C), each using 96 probe and primers pairs, to a final concentration of 0.2× for each of the 96 assays. Each set included *GUSB* as main reference gene. *18S* was included in set A. To increase sensitivity, a multiplexed preamplification process was performed for each pool on every 1.25 µl cDNA using 14 cycle cDNA preamplification step (95°C for 15 s and 60°C 4 min) and Taqman PreAmp Master Mix (Applied Biosystems) in a standard PCR thermocycler. To check reproducibility and test normalization approaches a sample calibrator made of the cDNA from the same AT biopsy total RNA sample was included in each plate for each of the 3 assay pools.

#### Quantitative PCR

Preamplified cDNA was diluted 1∶5 in 10 mmol/L Tris, 1 mmol/L EDTA. Diluted cDNA (2.25 µl) was added to 2.5 µl Taqman Universal PCR Master Mix (Applied Biosystems) and 0.25 µl GE Sample Loading Reagent (Fluidigm). In a separate tube, 3.5 µl of Taqman Assay was added to 3.5 µl Sample Loading Reagent. Five µl cDNA samples were loaded into the sample inlet wells, and 5 µl assay samples were loaded into assay detector inlets. For each plate, 1 well was loaded with H_2_0 as control for contamination and 1 well was loaded with the calibration sample. The chip was primed and placed into the NanoFlex Integrated fluidic circuit controller where 8 nl of cDNA and 1 nl of Assay were mixed. Real time PCR analysis was completed on the BioMark System (Fluidigm).

#### Data processing

Raw data obtained from the system's software using the default global threshold setting (BioMark Real-time PCR Analysis V2.1.1, Fluidigm) were checked using the graphical representation of the plate layout. Among the 414720 reactions investigated, 23 were rejected due to bubbles or instable ROX signal. All amplification curves were displayed for each well of the calibrator sample. When threshold for cycle did not meet quality criteria (i.e. threshold set in the linear phase of the amplification curve instead of the exponential phase), threshold value was set manually and the analysis parameters were changed to auto detector function for the corresponding gene and all the 15 Dynamic Arrays from the same set of genes. Wells with very high (>30) or absent (999) endogenous Ct resulted in exclusion of 10 genes with too low or inconsistent expression level. Based on data from the calibrator sample, 3 other genes with >20% outliers were removed from the analysis. This resulted in 271 gene transcripts with good quality expression data. Missing data were implemented using k nearest neighbour methods.

#### Optimal normalization method of adipose tissue gene expression

Different methods were assessed for real time qPCR data normalization. The simplest one is based on the 2^−ΔCt^ method [22] using an internal single reference gene. The use of a reference gene depends on both tissue and cell type as well as the experimental conditions. In order to capture the best control gene for the study of human subcutaneous AT gene expression, we previously measured the mRNA levels of 32 potential reference genes by RT-qPCR using TaqMan Human Endogenous Control Plate (Applied Biosystems) and the ABI Prism 7900HT Sequence Detection System (Applied Biosystems) on cDNA obtained from total RNA of 3 AT samples collected at each step of the program (see below) from 6 obese individuals representing 6 of the different European investigation centers (data not shown). A gene expression normalization factor was calculated using the analysis software Data Assist (Applied Biosystems) that uses the geNorm algorithm [47]. The β-glucuronidase (*GUSB*) transcript proved the best reference regarding the stability of mRNA level at different body weights and fat mass, and across replicate samples. Accordingly, in experiments including AT samples from lean, obese and weight-reduced individuals, *GUSB* also proved the best reference gene (data not shown). Consequently, *GUSB* was included in the 286 genes to be investigated. It is to be noted that among the 32 so-called house-keeping genes tested, four, ribonuclease P/MRP subunit (*POPP4*), translation initiation factor eIF-2B subunit α (*EIF2B1*), proteasome 26S subunit ATPase 4 (*PSMC4*) and glyceraldehyde-3-phosphate dehydrogenase (*GAPDH*) were selected as targets of interest and included in the list of transcripts to be quantified. Thus, they do not represent appropriate reference genes.

Global normalization approaches have proven efficient for microarray data. These methods use the whole data set. Large scale data normalization algorithms such as rank-invariant and quantile processes were tested as potential alternatives to the reference gene method [21]. Both assume that the expression level of the majority of the genes does not change. Of interest here, quantile normalization is dedicated to correct for inter-plate variations. Figure S2a shows the coefficient of variation for the 4 normalization methods compared to the raw dataset applied to the calibration sample assayed in the 48 plates. The quantile and rank-invariant methods were compared to the 2^−ΔCt^ calculation using the eukaryotic ribosomal protein 18S (*18S*), or *GUSB* as reference. When compared to non-normalized data, the quantile and rank-invariant methods did not improve variability while using *18S* greatly increased variability. The best performance was obtained with *GUSB*. All of the 4 methods tested provided similar results when applied to the whole data set for all samples (Figure S2b). Altogether, using *GUSB* appeared as the best process of normalizing these large scale data. Moreover, the relative expression level of this gene is similar across the 3 datasets (Figure S2c).

### DNA extraction and genotyping

Genomic DNA was extracted from the buffy coats with a salting out method. Genomic and amplified DNA samples were quality-checked, quantified and normalized to approximately 100 ng/ml and 2.0 µg before genotyping. High throughput SNP genotyping was carried out using the Illumina iScan Genotyping System (Illumina, San Diego, CA, USA). Seven hundred forty eight individuals were genotyped using the Illumina 660W-Quad SNP chip. SNP genotyping was done in accordance with manufacturer's protocols. The Integrated mapping information is based on NCBI's build 37. The coding sequences were investigated 15 kb downstream and 10 kb upstream. All SNP with a genotype frequency ≥95% and in Hardy-Weinberg equilibrium (*P*>0.05) were selected for further analyses. Among 3965 SNPs related to 252 genes, 2953 SNPs remained after data filtering.

### Statistical analysis

#### Exploratory and descriptive analyses

The R software (v2.11.0) was used for all statistical analyses. To normalize variance, skewed clinical data were logged and gene expression data were log2 transformed. One-way ANOVA was used for analysis of total RNA extraction optimization data. In order to obtain a global overview of the dataset principal component analysis was performed as a first step in the data analysis. PLS-DA was used to find the relationship between gene expression profiles and phenotypic features [48].

Shapiro-Wilk distribution normality and Bartlett's test variance homogeneity, which incorporate within-subject and between-subject variation, were applied to all gene expression data. A linear mixed effect model, including fixed effects as sex and metabolic syndrome status and introducing centre as random effect, was used as the model basis (nlme package) [49]. Tukey HSD was used as a post-hoc test. Supplementary factors are specified and the linear mixed effect model regression equations displayed in the legend of the tables. Genotype frequency and Hardy-Weinberg equilibrium were tested using SNP assoc package. Genetic association to gene expression was tested using the SimHap package. The effect of sex was tested using chi2 test and Fisher's exact test. A Benjamini-Hochberg procedure [50] was used to control the false discovery rate at 5% with the multtest package (mt.rawp2adjp).

#### Gaussian Graphical Model network analysis

Two association networks were constructed using Gaussian Graphical Models (GGM) [51] to represent the relationships between clinical variables and the male and female baseline gene sets. An intertwined, sparse LASSO (least absolute shrinkage and selection operator) approach [52], which is able to handle large-scale data and implemented in the R package simone [53], was employed to ensure that the networks were inferred based on the assumption that common functionality exists in both men and women. This assumption is integrated in the GGM by means of a covariance matrix that mixes the gender-specific covariance and the global covariance over men and women together. The networks were set to a density, the number of edges in the network divided by the number of pairs of vertices (i.e. the number of possible edges), of 15%, which corresponds approximately to the maximum number of edges to obtain a readable network. Dependency networks were built using partial correlations as a measure of dependency and an undirected GGM where the variables are connected if and only if their partial correlation is significantly non zero. Unlike pairwise measure of associations, such as Pearson correlation coefficients, partial correlation provides a stronger criterion for dependency by adjusting for confounding effects, and thus removes spurious associations to a large extent. This is useful in order to integrate multiple layers of information, as it filters out false positives by discovering only high confidence direct interactions.

To study the dietary intervention, two networks were inferred to model the relationships between log scaled ratios from baseline to the end of LCD and from baseline to the end of WMD. A sparse LASSO inference method, as implemented in the R package simone [52], was used with the “AND” policy, which tends to produce less false positive edges, and a 15% network density constraint. The co-expression pattern networks were laid out using the network analysis package Gephi (gephi.org) with the Fruchterman Reingold algorithm [54].

## Supporting Information

Figure S1 Optimization of human adipose tissue total RNA extraction. (a) Relation between adipose tissue weight and total RNA amount. *n* = 84. (b) Comparison of different adipose tissue preparation and storage conditions (black bars, after 1 month storage; open bars, after 1 year storage) on total RNA recovery : immediate storage at −80°C (−80°C), flash-freezing in liquid nitrogen and storage at −80°C (N_2_), overnight incubation in RNAlater RNA Stabilization Reagent (Qiagen) at 4°C and storage at −80°C (RNA later), storage at −80°C in QIAzol Lysis Reagent (QIAzol) and storage at −80°C after homogeneization in QIAzol Lysis Reagent with ultra-Turax (Turax). *n* = 4. (c) Effect of duration of storage at −80°C on adipose tissue total RNA yield with comparison of flash-freezing in liquid nitrogen before storage (black bars) and storage in QIAzol Lysis Reagent (open bars). *n* = 3–15.
(TIF)

Figure S2 Validation of quantitative PCR normalization methods. (a) Comparison of methods applied to the calibration sample. Bar charts represent coefficient of variation of calibration sample gene expression level using 4 different normalization methods compared to the raw dataset. The quantile and rank-invariant methods are compared to the delta Ct calculation with the *18S*, or *GUSB*, data from the corresponding preamplified cDNA. (b) Comparison of methods applied to the whole dataset. Bar charts represent coefficient of variation of calibration sample gene expression level using 4 different normalization methods compared to the raw dataset. The quantile and rank-invariant methods are compared to the delta Ct calculation with the *18S*, or *GUSB* data from the corresponding preamplified cDNA. (c) *GUSB* expression levels shown as cycle threshold across the 3 datasets (A, B and C) related to the 3 groups of 96 genes of interest (95 genes plus *GUSB*) respectively.
(TIF)

Figure S3 Flow chart of DiOGenes analyses. Individuals from group A are those with gene expression data available at baseline, at the end of the 8-week calorie restriction and at the end of the 26-week weight follow-up. Individuals from group B are those with gene expression data available only at baseline. BAS: baseline, LCD: low calorie diet, WMD: weight maintenance diet. MS: metabolic syndrome. BMI: body mass index. SNP: single nucleotide polymorphism. Dotted arrows represent the statistical analyses output.
(TIF)

Figure S4 Exploratory analyses of adipose tissue gene expression from 515 subjects at baseline. (a) Plots of PLS-DA (Partial Least Square-Discriminant Analysis) used for explaining differences between 180 men (black circles) and 335 women (open circles) (R^2^ = 0.257; Q^2^ = 0.259). (b) Plot of PLS-DA used for explaining differences between 309 non-metabolic syndrome (grey triangles) and 206 metabolic syndrome (black squares) individuals (R^2^ = 0.270; Q^2^ = 0.106).
(TIF)

Figure S5 Topology of the male and female networks at baseline. A dependency network was constructed from selected gene expression and bio-clinical data from 180 men (a) and 335 women (b). Each node is a gene or a bio-clinical parameter. Node degree is indicated with node size. Node color indicates betweenness centrality metric that measures how often a node appears on shortest paths between nodes in the network, from red (high level) to green (low level). Betweenness centrality indicates influential nodes for highest values. The variables are connected by an edge only if their partial correlation is significantly nonzero. Edge thickness is proportional to the strength of correlation. Edge color indicates positive (red) or negative (light green) correlation. The orange ellipse indicates the lipogenic module.
(TIF)

Table S1 Methods for extraction of total RNA from human adipose tissue. *: 0, degradation; §, low purity with spurious genomic DNA; §§, high quality based on 28 to 18S RNA ratio and absence of genomic DNA. RNA quality was checked using ethidium bromide stained agarose gels. Concentration was determined using Nanodrop spectrophotometer. Values refer to means ± SEM.
(DOCX)

Table S2 Clinical and biological parameters of obese individuals before dietary intervention. Values refer to means ± SEM. *** p<0.001, ** p<0.01, * p<0.1 between non-metabolic syndrome (non-MS) and metabolic syndrome (MS) subjects as estimated using linear mixed effect model ran separately for men and women with metabolic syndrome status as fixed and centre as random effect. BMI is body mass index [weight (kg)/height^2^ (m)]. SBP and DBP are, respectively, systolic and diastolic blood pressures. HDL-C and LDL-C are, respectively, high and low density lipoprotein-cholesterol. The homeostatic model assessment (HOMA-IR) is method to assess insulin resistance [glucose (mM) x insulin (mU/l)/22.5].
(DOCX)

Table S3 Description of target genes. Cell marker column refers to the adipose tissue cell type.
(DOCX)

Table S4Genes with opposite regulation during low calorie diet and weight maintenance diet phases. A linear mixed effect model was run separately for men and women with time point (clinical investigation day, CID), weight at baseline and weight change as fixed effect. Centre and subject were entered as random effect. Diet was included as supplementary fixed effect in the model investigating genes during weight maintenance diet. The regressions equations tested without and with weight are displayed below:
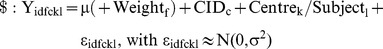


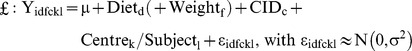
Y is the log2 expression value for gene i, in subject l, and centre k. The random term ε represents the random error that was assumed to be normally distributed. The Tukey HSD was used as post-hoc test. The Benjamini-Hochberg procedure was used to control for multiple testing. Only significant genes common to men and women and weight-independent genes were selected. *: Values refer to median mRNA level fold change from 311 subjects (107 men, 204 women).(DOCX)

Table S5Genes regulated at the end of the dietary intervention. A linear mixed effect model was ran separately for men and women with weight, clinical investigation day (CID) and diet as fixed, and centre and subject as random effect. The regressions equations tested without and with weight are displayed below:
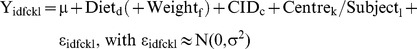
Y is the log2 expression value for gene i, in subject l, and centre k. The random term ε represents the random error that was assumed to be normally distributed. The Tukey HSD was used as post-hoc test. The Benjamini-Hochberg procedure was used to control for multiple testing. Only significant genes common to men and women and weight-independent genes are shown. *: Values refer to median mRNA level fold change from 311 subjects (107 men, 204 women).(DOCX)

Table S6Markers of weight loss and weight regain during the weight maintenance diet phase. *: Values refer to ratio of mean mRNA level fold change from CID2 to CID3 between 31 women who gained (Regain) and 29 who lost (Loss) at least 50% of the weight lost during calorie restriction. £: Values refer to median mRNA level fold change between CID2 and CID3 in 31 women who gained at least 50% of the weight lost during calorie restriction. §: Values refer to median mRNA level fold change between CID2 and CID3 in 29 women who continued to lose at least 50% of the weight lost during calorie restriction. A linear mixed effect model was ran separately for men and women with weight, CID and diet as fixed, and centre and subject as random effect. The regression equation tested is displayed below:
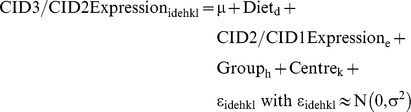
CID3/CID2Expression is the log2 expression value for gene i, in subject l, and centre k. The random term ε represents the random error that was assumed to be normally distributed. The Tukey HSD was used as post-hoc test. The Benjamini-Hochberg procedure was used to control for multiple testing. CID1, CID2 and CID3 are, respectively, clinical investigation days at baseline, after the 8-week calorie restriction and after the 26-week weight maintenance diet.(DOCX)

Table S7Sexual dimorphism of adipose tissue gene expression in 515 obese individuals. A linear mixed effect model was ran with gender as fixed and centre as random effect. The regression equation tested without and with fat mass (% of body weight) is displayed below:
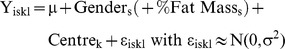
Y is the log2 expression value for gene i, in subject l, and centre k. The random term ε represents the random error that was assumed to be normally distributed. The Tukey HSD was used as post-hoc test. The Benjamini-Hochberg procedure was used to control for multiple testing. Only genes consistently significant among all phases of the dietary intervention were considered (see Figure S3). Bold indicates genes which sexual dimorphism is influenced by fat mass. *: Values refer to ratio of mean mRNA levels at baseline between 180 men and 335 women.(DOCX)

Table S8Differential adipose tissue gene expression according to presence and absence of metabolic syndrome in 515 obese individuals. P-value between non-metabolic syndrome and metabolic syndrome subjects as estimated using linear mixed effect model ran separately for men and women with metabolic syndrome status as fixed and centre as random effect, Tukey HSD as post-hoc test and Benjamini-Hochberg to control for multiple testing. The regression equation tested is displayed below:
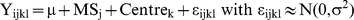
Y is the log2 expression value for gene i, in subject l, and centre k. The random term ε represents the random error that was assumed to be normally distributed. MS and non-MS are, respectively, subjects categorized according to the occurrence of metabolic syndrome [11], or not. The Tukey HSD was used as post-hoc test. *: Values refer to ratio of mean mRNA levels between metabolic syndrome (92 men, 114 women) and non-metabolic syndrome (88 men, 221 women) subjects at baseline (see Figure S3).(DOCX)

Table S9Obesity-related genes in women along the dietary intervention. Values refer to ratio of mean mRNA level from highest and lowest BMI decile subjects in 204 women from group A. CID1, CID2 and CID3 are, respectively, clinical investigation days at baseline, after the 8-week calorie restriction and after the 26-week weight maintenance diet. A linear mixed effect model was run with BMI as fixed and centre as random effect at each time point. Diet was entered as supplementary fixed effect in the model investigating genes during weight maintenance diet. The regressions equations tested without and with change in BMI are displayed below:
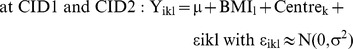


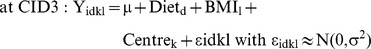
Y is the log2 expression value for gene i, in subject l, centre k and Diet d. The random term ε represents the random error that was assumed to be normally distributed. The Benjamini-Hochberg procedure was used to control for multiple testing.(DOCX)

Table S10 Genes selected for network analysis at baseline.
(DOCX)

Table S11 Lipogenic and glycolytic genes selected for network analysis during dietary intervention. Genes were selected as part of the lipogenic module observed in Figure S5 with other genes encoding proteins involved in lipogenesis or glucose metabolism.
(DOCX)

Table S12SNPs showing associations with adipose tissue gene expression at baseline. Gene expression data were log transformed. Association of 2953 SNPs with gene expression was tested at baseline in 422 individuals (148 men and 274 women) using a linear mixed model with gender (and BMI) as fixed and centre (and diet) as random effect assuming a linear log-additive allele-dose effect with each SNP coded 0, 1 and 2 according to the number of minor alleles an individual carries (0 = wild-type, 1 = heterozygote, 2 = homozygote). The regression equation displayed below was tested without then with BMI:
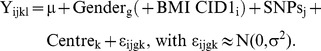
Y is the log2 expression value for gene i, in subject l, and centre k. The random term ε represents the random error that was assumed to be normally distributed. Gender difference between genotype distribution was investigated using a chi2 test to test the independence between the people belonging to the individual and the value criteria. For regression expected values >5, the non-parametric Fisher's exact test was used. The Benjamini-Hochberg procedure was used to control for multiple testing. CID1 is clinical investigation day at baseline.(DOCX)

Table S13Significativity of SNP association with adipose tissue gene expression along the dietary program. To test the validity of the association of the SNPs significant at CID1 with gene expression along the dietary program, a linear mixed model was ran with gene expression data at CID1, CID2 and CID3 according to the regression equations displayed below. Each SNP was evaluated under a log additive model assuming a linear allele-dose effect (0 = Wild-type, 1 = heterozygote, 2 = homozygote). The regression equations were tested without then with BMI.
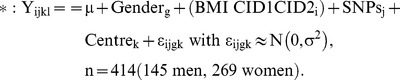


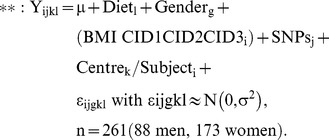


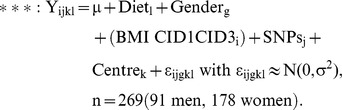
Y is the log2 expression value for gene i, in subject l, and centre k. The random term ε represents the random error that was assumed to be normally distributed. The Benjamini-Hochberg procedure was used to control for multiple testing. CID1, CID2 and CID3 are, respectively, clinical investigation days at baseline, after the 8-week calorie restriction and after the 26-week weight maintenance diet.(DOCX)
