# Supplementary figures and images for: Determinants of Human Adipose Tissue Gene Expression: Impact of Diet, Sex, Metabolic Status, and Cis Genetic Regulation
Source: PLoS Genet. 2012 Sep 27;8(9):e1002959. doi: 10.1371/journal.pgen.1002959 (PMC3459935; doi:10.1371/journal.pgen.1002959)

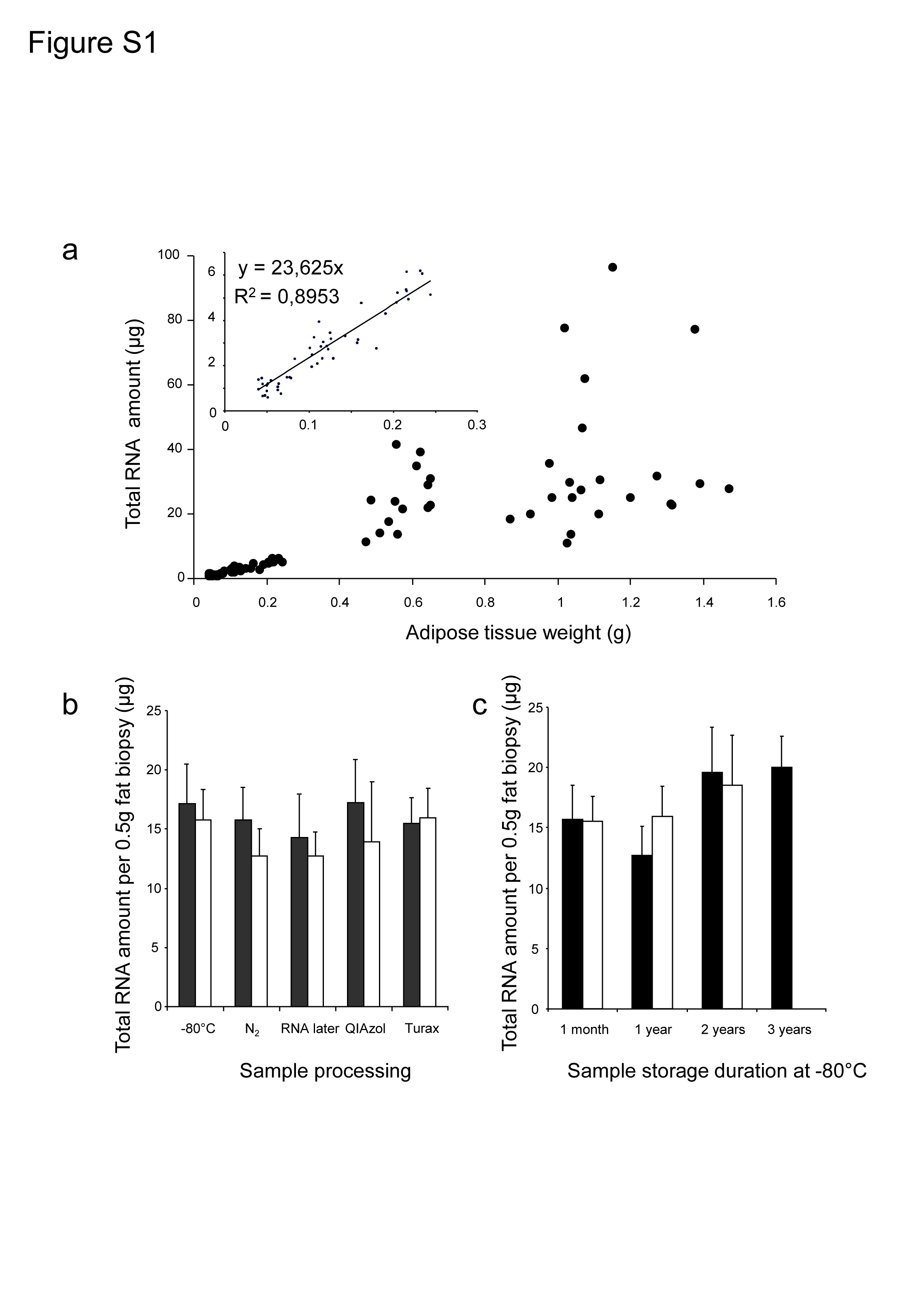

Supplement: Figure S1 — Optimization of human adipose tissue total RNA extraction. (a) Relation between adipose tissue weight and total RNA amount. n = 84. (b) Comparison of different adipose tissue preparation and storage conditions (black bars, after 1 month storage; open bars, after 1 year storage) on total RNA recovery : immediate storage at −80°C (−80°C), flash-freezing in liquid nitrogen and storage at −80°C (N2), overnight incubation in RNAlater RNA Stabilization Reagent (Qiagen) at 4°C and storage at −80°C (RNA later), storage at −80°C in QIAzol Lysis Reagent (QIAzol) and storage at −80°C after homogeneization in QIAzol Lysis Reagent with ultra-Turax (Turax). n = 4. (c) Effect of duration of storage at −80°C on adipose tissue total RNA yield with comparison of flash-freezing in liquid nitrogen before storage (black bars) and storage in QIAzol Lysis Reagent (open bars). n = 3–15. (TIF) [file pgen.1002959.s001.tif]

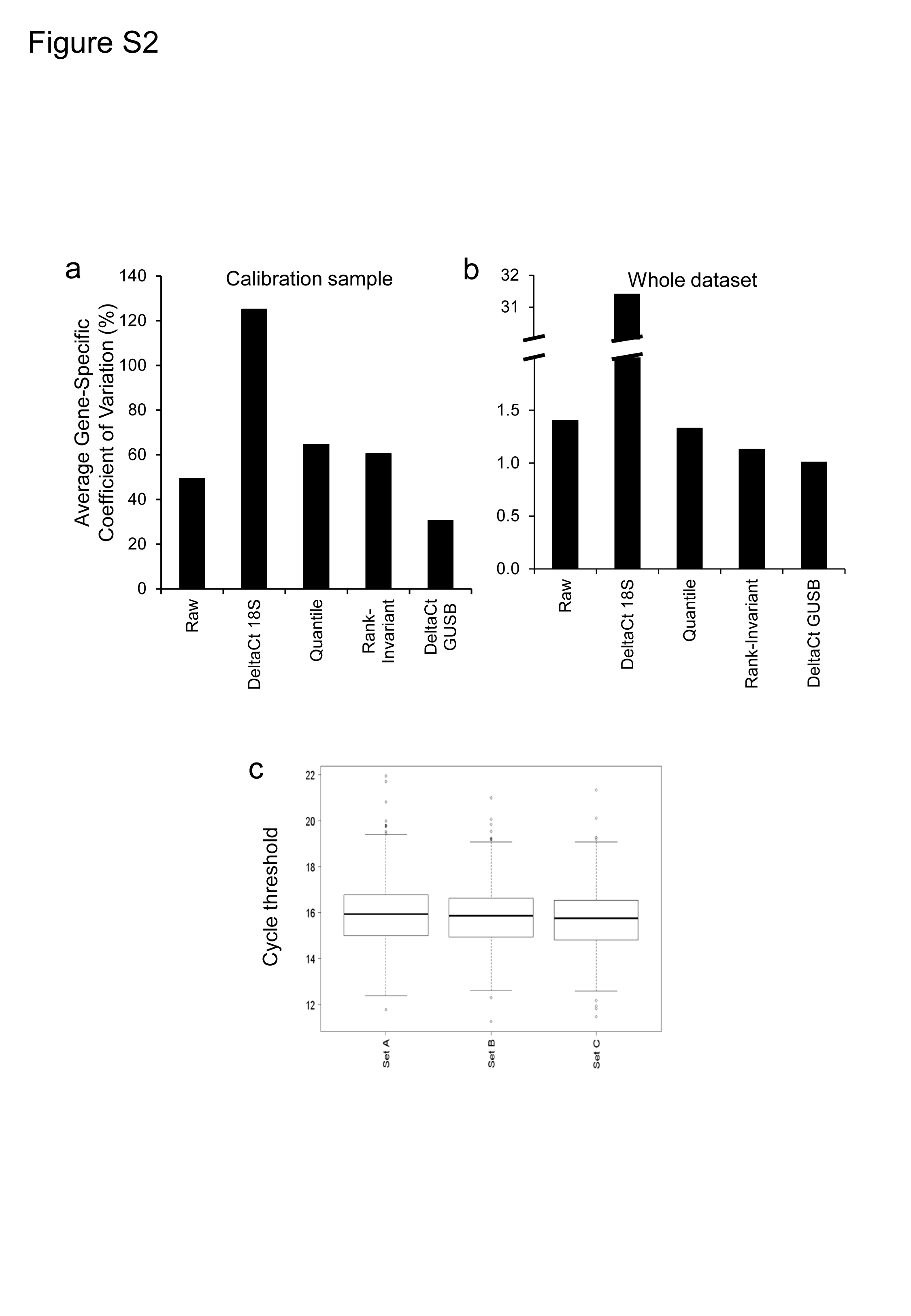

Supplement: Figure S2 — Validation of quantitative PCR normalization methods. (a) Comparison of methods applied to the calibration sample. Bar charts represent coefficient of variation of calibration sample gene expression level using 4 different normalization methods compared to the raw dataset. The quantile and rank-invariant methods are compared to the delta Ct calculation with the 18S, or GUSB, data from the corresponding preamplified cDNA. (b) Comparison of methods applied to the whole dataset. Bar charts represent coefficient of variation of calibration sample gene expression level using 4 different normalization methods compared to the raw dataset. The quantile and rank-invariant methods are compared to the delta Ct calculation with the 18S, or GUSB data from the corresponding preamplified cDNA. (c) GUSB expression levels shown as cycle threshold across the 3 datasets (A, B and C) related to the 3 groups of 96 genes of interest (95 genes plus GUSB) respectively. (TIF) [file pgen.1002959.s002.tif]

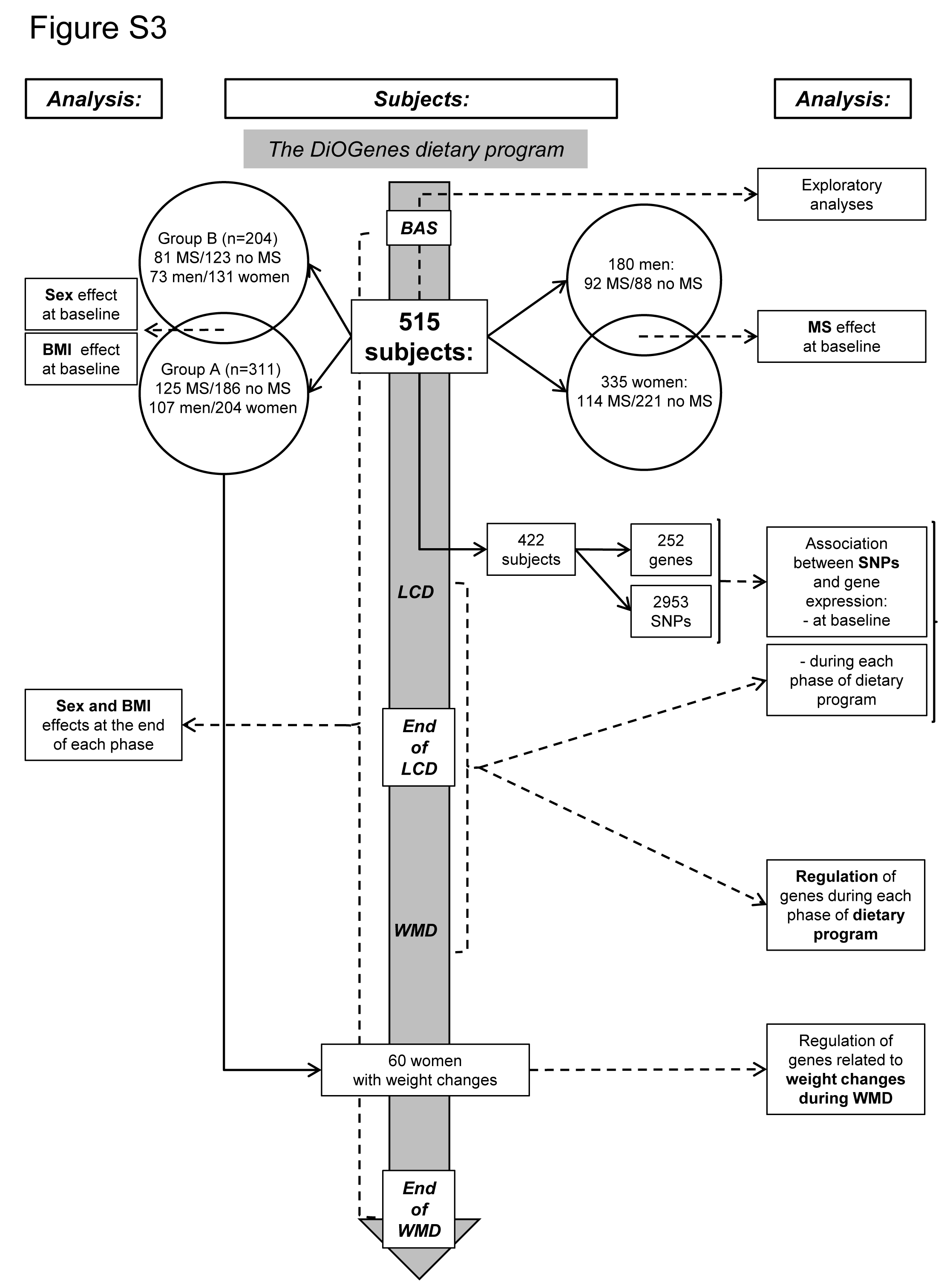

Supplement: Figure S3 — Flow chart of DiOGenes analyses. Individuals from group A are those with gene expression data available at baseline, at the end of the 8-week calorie restriction and at the end of the 26-week weight follow-up. Individuals from group B are those with gene expression data available only at baseline. BAS: baseline, LCD: low calorie diet, WMD: weight maintenance diet. MS: metabolic syndrome. BMI: body mass index. SNP: single nucleotide polymorphism. Dotted arrows represent the statistical analyses output. (TIF) [file pgen.1002959.s003.tif]

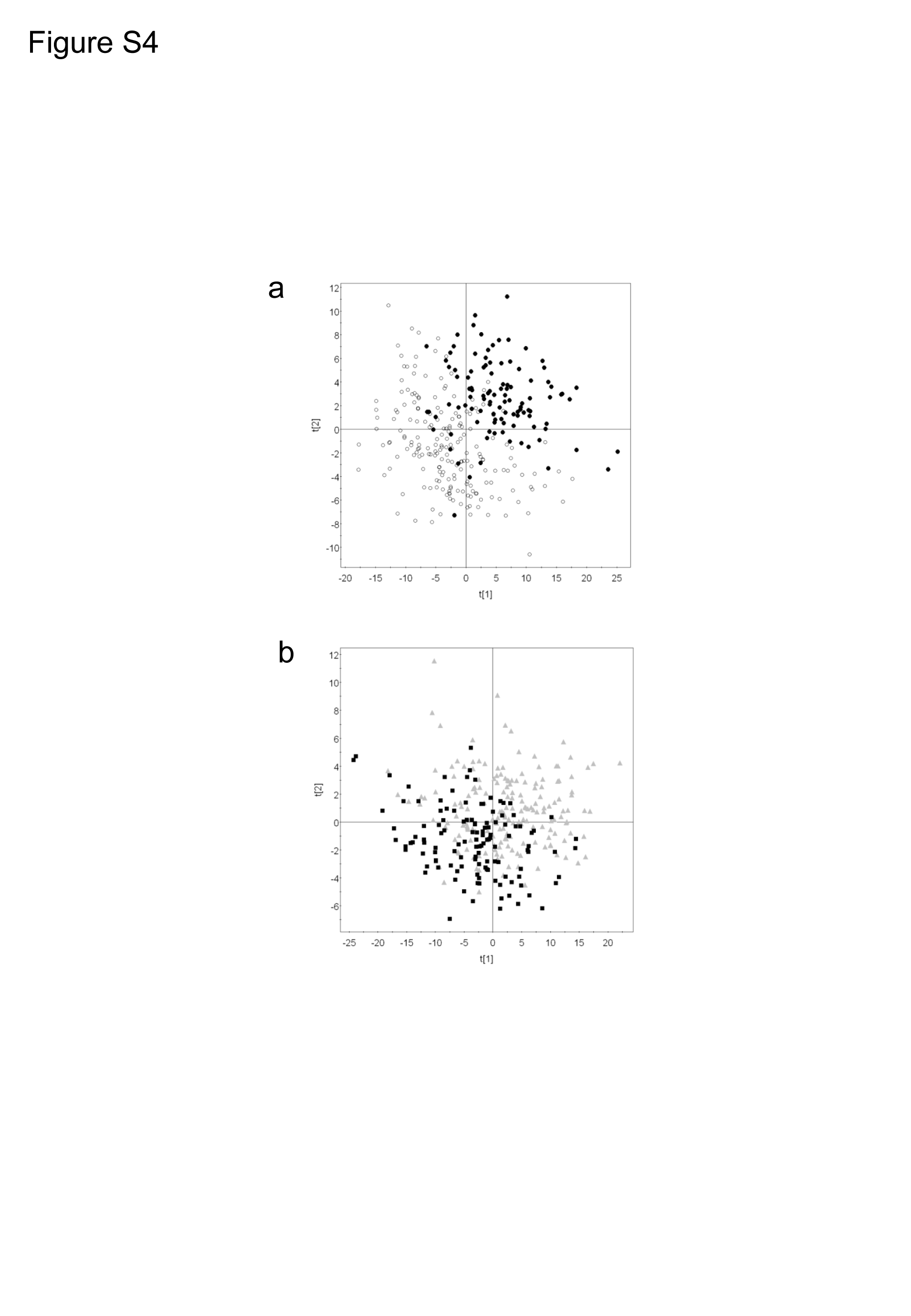

Supplement: Figure S4 — Exploratory analyses of adipose tissue gene expression from 515 subjects at baseline. (a) Plots of PLS-DA (Partial Least Square-Discriminant Analysis) used for explaining differences between 180 men (black circles) and 335 women (open circles) (R2 = 0.257; Q2 = 0.259). (b) Plot of PLS-DA used for explaining differences between 309 non-metabolic syndrome (grey triangles) and 206 metabolic syndrome (black squares) individuals (R2 = 0.270; Q2 = 0.106). (TIF) [file pgen.1002959.s004.tif]

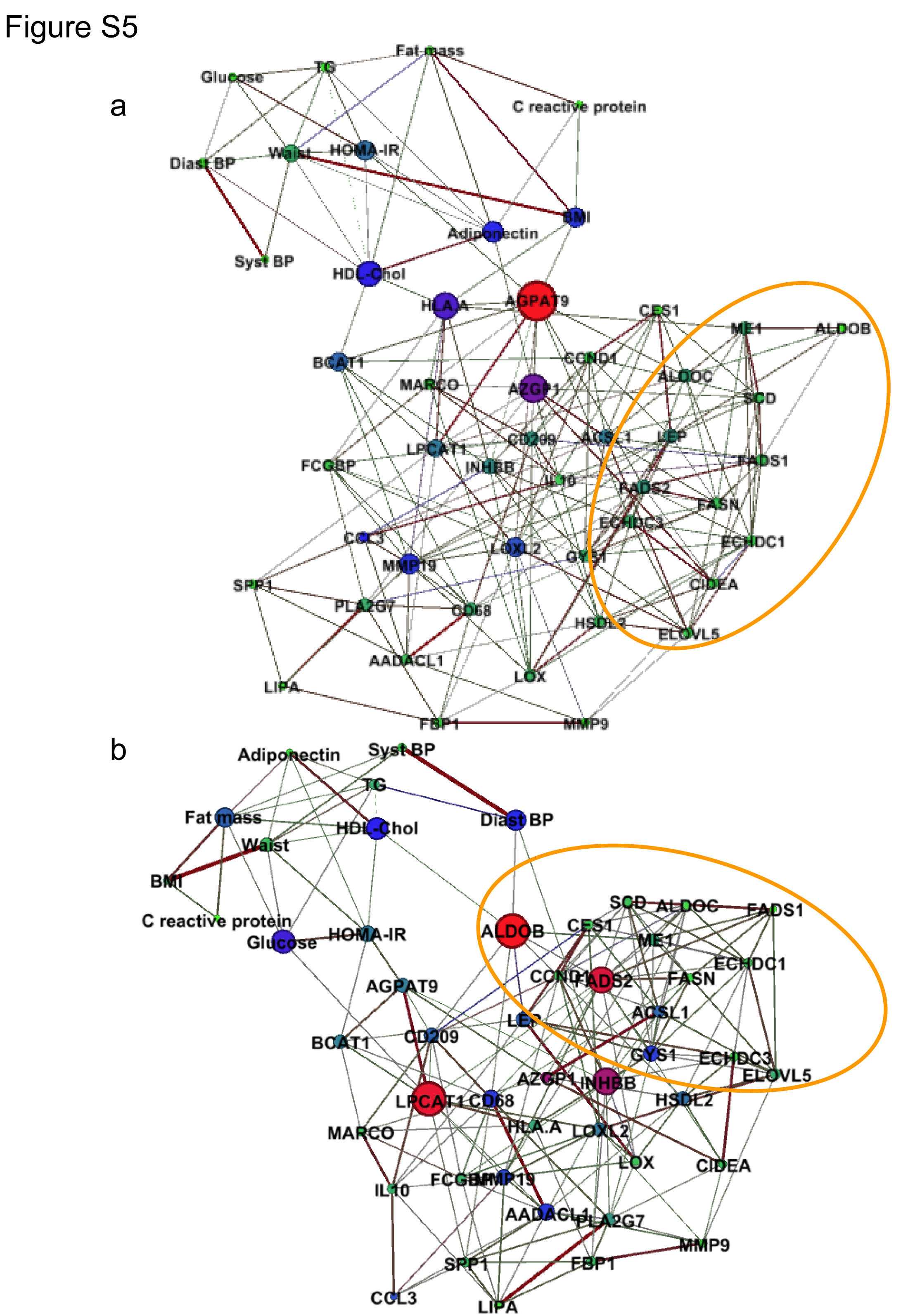

Supplement: Figure S5 — Topology of the male and female networks at baseline. A dependency network was constructed from selected gene expression and bio-clinical data from 180 men (a) and 335 women (b). Each node is a gene or a bio-clinical parameter. Node degree is indicated with node size. Node color indicates betweenness centrality metric that measures how often a node appears on shortest paths between nodes in the network, from red (high level) to green (low level). Betweenness centrality indicates influential nodes for highest values. The variables are connected by an edge only if their partial correlation is significantly nonzero. Edge thickness is proportional to the strength of correlation. Edge color indicates positive (red) or negative (light green) correlation. The orange ellipse indicates the lipogenic module. (TIF) [file pgen.1002959.s005.tif]
