# Supplementary material for: Determinants of Human Adipose Tissue Gene Expression: Impact of Diet, Sex, Metabolic Status, and Cis Genetic Regulation
Source: PLoS Genet. 2012 Sep 27;8(9):e1002959. doi: 10.1371/journal.pgen.1002959 (PMC3459935; doi:10.1371/journal.pgen.1002959)
Supplement: Table S1 — Methods for extraction of total RNA from human adipose tissue. *: 0, degradation; §, low purity with spurious genomic DNA; §§, high quality based on 28 to 18S RNA ratio and absence of genomic DNA. RNA quality was checked using ethidium bromide stained agarose gels. Concentration was determined using Nanodrop spectrophotometer. Values refer to means ± SEM. (DOCX) [file pgen.1002959.s006.docx]

**Table S1 Methods for extraction of total RNA from human adipose tissue.**

| Methods | Total RNA Yield (µg/g fat) | Quality* | n |
| --- | --- | --- | --- |
| RiboPure (Ambion) | 18.7 ± 3.1 | § | 6 |
| RNA STAT-60 (Tel-Test Inc) | 0 | 0 | 6 |
| NucleoSpin RNA II (Macherey Nagel) | 0.13 ± 0.05 | § | 6 |
| RNeasy Mini (QIAGEN) | 21.3 ± 5.7 | §§ | 6 |
| RNeasy Lipid Tissue Mini (QIAGEN) | 36.2 ± 6.5 | § | 5 |
| In-House modified RNeasy Lipid Tissue Mini (QIAGEN) | 43.9 ± 15.7 | §§ | 12 |
