# Supplementary material for: Determinants of Human Adipose Tissue Gene Expression: Impact of Diet, Sex, Metabolic Status, and Cis Genetic Regulation
Source: PLoS Genet. 2012 Sep 27;8(9):e1002959. doi: 10.1371/journal.pgen.1002959 (PMC3459935; doi:10.1371/journal.pgen.1002959)
Supplement: Table S2 — Clinical and biological parameters of obese individuals before dietary intervention. Values refer to means ± SEM. *** p<0.001, ** p<0.01, * p<0.1 between non-metabolic syndrome (non-MS) and metabolic syndrome (MS) subjects as estimated using linear mixed effect model ran separately for men and women with metabolic syndrome status as fixed and centre as random effect. BMI is body mass index [weight (kg)/height2 (m)]. SBP and DBP are, respectively, systolic and diastolic blood pressures. HDL-C and LDL-C are, respectively, high and low density lipoprotein-cholesterol. The homeostatic model assessment (HOMA-IR) is method to assess insulin resistance [glucose (mM) x insulin (mU/l)/22.5]. (DOCX) [file pgen.1002959.s007.docx]

**Table S2 Clinical and biological parameters of obese individuals before dietary intervention.**

| a. Group A (n=311). |  |  |  |  |  |
| --- | --- | --- | --- | --- | --- |
|  | Men | |  | Women | |
|  | n = 107 | |  | n = 204 | |
|  | non-MS | MS |  | non-MS | MS |
|  | n = 51 | n = 56 |  | n = 135 | n = 69 |
| Age (years) | 43.8 ± 5.0 | 43.2 ± 6.3 |  | 41.5 ± 6.7 | 42.5 ± 6.3 |
| Height (m) | 1.8 ± 0.1 | 1.8 ± 0.1 |  | 1.7 ± 0.1 | 1.7 ± 0.1 |
| Weight (kg) | 106.6 ± 16.5 | 107.7 ± 18.8 |  | 91.7 ± 13.6 | 99.2 ± 16.2*** |
| BMI (kg/m^2^) | 33.6 ± 4.4 | 34.1 ± 5.0 |  | 33.4 ± 4.4 | 36.2 ± 4.8*** |
| Fat mass (%) | 32.4 ± 5.5 | 31.3 ± 5.4 |  | 42.6 ± 4.9 | 44.4 ± 4.8* |
| Waist (cm) | 112.7 ± 10.8 | 113.5 ± 12 |  | 100.5 ± 9.6 | 108.1 ± 12.5*** |
| SBP (mmHg) | 127.8 ± 11.7 | 138.4 ± 10.4*** |  | 118.7 ± 12.2 | 132 ± 14.3*** |
| DBP (mmHg) | 79.6 ± 9.8 | 85.1 ± 9.3* |  | 73 ± 9.5 | 80.6 ± 10.9*** |
| Triglycerides (mmol/l) | 1.3 ± 0.6 | 1.9 ± 0.8*** |  | 1.0 ± 0.4 | 1.6 ± 0.6*** |
| Total cholesterol (mmol/l) | 5.1 ± 1.0 | 5.1 ± 1.3 |  | 4.8 ± 0.9 | 4.9 ± 0.9 |
| HDL-C (mmol/l) | 1.2 ± 0.3 | 1.0 ± 0.2*** |  | 1.4 ± 0.3 | 1.1 ± 0.2*** |
| LDL-C (mmol/l) | 3.3 ± 0.9 | 3.2 ± 1.0 |  | 2.9 ± 0.7 | 3.1 ± 0.8 |
| Glucose (mmol/l) | 5.1 ± 0.5 | 5.3 ± 0.6** |  | 4.9 ± 0.5 | 5.5 ± 0.8*** |
| Insulin (µIU/ml) | 10.5 ± 6.1 | 13.5 ± 6.6** |  | 8.7 ± 4.3 | 13.1 ± 6.7*** |
| HOMA-IR | 2.4 ± 1.4 | 3.2 ± 1.7** |  | 1.9 ± 1.0 | 3.3 ± 1.9*** |
| C-reactive protein (mg/ml) | 3.2 ± 2.2 | 2.4 ± 2.2* |  | 4.1 ± 3.6 | 5.1 ± 3.5** |
| Adiponectin (µg/ml) | 9.0 ±3.7 | 7.2 ± 2.5* |  | 11.1 ± 4.9 | 9.2 ± 4.5*** |
|  |  |  |  |  |  |
|  |  |  |  |  |  |
| b. Group B (n=204). |  |  |  |  |  |
|  | Men | |  | Women | |
|  | n = 73 | |  | n = 131 | |
|  | non-MS | MS |  | non-MS | MS |
|  | n = 37 | n = 36 |  | n = 86 | n = 45 |
| Age (years) | 41 ± 6.4 | 42.3 ± 5.7 |  | 39 ± 6.1 | 41.9 ± 6.3* |
| Height (m) | 1.8 ± 0.1 | 1.8 ± 0.1 |  | 1.7 ± 0.1 | 1.7 ± 0.1 |
| Weight (kg) | 108.9 ± 15.8 | 112.9 ± 17.7 |  | 93.9 ± 15.1 | 100.2 ± 14.8* |
| BMI (kg/m^2^) | 34.5 ± 4.4 | 35.5 ± 4.6 |  | 34.4 ± 4.9 | 36.3 ± 4.8* |
| Fat mass (%) | 33.6 ± 5.1 | 34.8 ± 7.0 |  | 44.2 ± 5.1 | 43.3 ± 4.2 |
| Waist (cm) | 113.7 ± 11.4 | 118.8 ± 12.7* |  | 104.1 ± 13.4 | 108.4 ± 9.9** |
| SBP (mmHg) | 125.3 ± 8.8 | 135.9 ± 13.3*** |  | 115.3 ± 9.9 | 130.6 ± 11.9*** |
| DBP (mmHg) | 76.7 ± 9.2 | 84.9 ± 9.5*** |  | 72.1 ± 9.7 | 82.3 ± 10.1*** |
| Triglycerides (mmol/l) | 1.2 ± 0.3 | 1.8 ± 0.6*** |  | 1.1 ± 0.3 | 1.6 ± 0.7*** |
| Total cholesterol (mmol/l) | 5.1 ± 0.8 | 5.3 ± 1.2 |  | 4.9 ± 1.0 | 5.3 ± 0.9* |
| HDL-C (mmol/l) | 1.3 ± 0.2 | 1.0 ± 0.2*** |  | 1.4 ± 0.2 | 1.1 ± 0.2*** |
| LDL-C (mmol/l) | 3.3 ± 0.7 | 3.5 ± 1.0 |  | 3.1 ± 0.9 | 3.5 ± 0.9** |
| Glucose (mmol/l) | 5.1 ± 0.5 | 5.6 ± 0.7*** |  | 4.9 ± 0.4 | 5.2 ± 0.8*** |
| Insulin (µIU/ml) | 12.1 ± 6.8 | 15.2 ± 8.3** |  | 9.2 ± 6.5 | 11.5 ± 7.3* |
| HOMA-IR | 2.8 ± 1.6 | 3.9 ± 2.6*** |  | 2.0 ± 1.3 | 2.7 ± 1.8** |
| C-reactive protein (mg/ml) | 3.2 ± 3.3 | 4.9 ± 3.4* |  | 4.5 ± 4.1 | 5.8 ± 4.5* |
| Adiponectin (µg/ml) | 8.1 ± 3.8 | 7.5 ± 3.1 |  | 10.2 ± 4.8 | 10 ± 3.9 |
