# Supplementary material for: Determinants of Human Adipose Tissue Gene Expression: Impact of Diet, Sex, Metabolic Status, and Cis Genetic Regulation
Source: PLoS Genet. 2012 Sep 27;8(9):e1002959. doi: 10.1371/journal.pgen.1002959 (PMC3459935; doi:10.1371/journal.pgen.1002959)
Supplement: Table S3 — Description of target genes. Cell marker column refers to the adipose tissue cell type. (DOCX) [file pgen.1002959.s008.docx]

**Table S3 Description of target genes**

| **Gene Symbol** | **Gene Name** | **Biological Process** | **Function** | **Cell Marker** | **References** |
| --- | --- | --- | --- | --- | --- |
| AACS | acetoacetyl-CoA synthetase | lipid metabolism | lipogenesis | adipocyte | 12 |
| AADACL1 | neutral cholesterol ester hydrolase 1 | lipid metabolism | cholesterol metabolism | macrophage | 12; 15; 16 |
| ABHD5 | abhydrolase domain containing 5 | lipid metabolism | lipolysis | adipocyte | 15; 16 |
| ACACB | acetyl-Coenzyme A carboxylase beta | lipid metabolism | lipogenesis | adipocyte | 15; 16 |
| ACAD9 | acyl-Coenzyme A dehydrogenase family, member 9 | lipid metabolism | lipogenesis | adipocyte | 12 |
| ACADM | acyl-Coenzyme A dehydrogenase, C-4 to C-12 straight chain | lipid metabolism | fatty acid oxydation | adipocyte | 12; 15; 16 |
| ACAT1 | acetyl-Coenzyme A acetyltransferase 1 | lipid metabolism | lipogenesis | adipocyte | 15; 16 |
| ACOX1 | acyl-Coenzyme A oxidase 1, palmitoyl | lipid metabolism | fatty acid oxydation | adipocyte | 12 |
| ACSL1 | acyl-CoA synthetase long-chain family member 1 | lipid metabolism | lipogenesis | adipocyte | 12; 15; 16 |
| ACSS2 | acyl-CoA synthetase short-chain family member 2 | lipid metabolism | lipogenesis | adipocyte | 12; 15; 16 |
| ACTR3 | ARP3 actin-related protein 3 homolog (yeast) | signal transduction | endocytosis signaling |  | 12; 14; 16 |
| ADH1A | alcohol dehydrogenase 1A (class I), alpha polypeptide | carbohydrate metabolism | aerobic glycolysis | adipocyte | 12; 15; 16 |
| ADH1C | alcohol dehydrogenase 1C (class I), gamma polypeptide | carbohydrate metabolism | aerobic glycolysis | adipocyte | 12; 15; 16 |
| ADHFE1 | alcohol dehydrogenase, iron containing, 1 | ketone metabolism | ketone metabolism | adipocyte | 12; 15; 16 |
| AES | amino-terminal enhancer of split | transcription | transcription |  | 12; 14; 16 |
| AGPAT1 | 1-acylglycerol-3-phosphate O-acyltransferase 1 (lysophosphatidic acid acyltransferase, alpha) | lipid metabolism | lipogenesis |  | 14 |
| AGPAT9 | 1-acylglycerol-3-phosphate O-acyltransferase 9 | lipid metabolism | lipogenesis |  | 15; 16 |
| AK2 | adenylate kinase 2 | purine metabolism | purine metabolism | adipocyte | 12; 15; 16 |
| ALDH6A1 | aldehyde dehydrogenase 6 family, member A1 | amino acid metabolism | amino acid metabolism | adipocyte | 12; 15; 16 |
| ALDOB | aldolase B, fructose-bisphosphate | carbohydrate metabolism | aerobic glycolysis |  | Unpublished |
| ALDOC | aldolase C, fructose-bisphosphate | carbohydrate metabolism | aerobic glycolysis | adipocyte | 12 |
| ALOX12 | arachidonate 12-lipoxygenase | lipid metabolism | arachidonic acid metabolism |  | 13 |
| ALOX5 | arachidonate 5-lipoxygenase | lipid metabolism | arachidonic acid metabolism |  | 15; 16 |
| ANG | angiogenin, ribonuclease, RNase A family, 5 | secreted factor | angiogenesis |  | 12; 16 |
| AP2M1 | adaptor-related protein complex 2, mu 1 subunit | signal transduction | intracellular trafficking |  | 15; 16 |
| AQP1 | aquaporin 1 (Colton blood group) | lipid metabolism | transport |  | 12; 16 |
| AQP7 | aquaporin 7 | lipid metabolism | transport | adipocyte | 12; 15; 16 |
| ARF3 | ADP-ribosylation factor 3 | transport | transport |  | 14 |
| ARPC1A | actin related protein 2/3 complex, subunit 1A, 41kDa | cell structure | cytoskeleton organization | adipocyte | 14 |
| ATF3 | activating transcription factor 3 | transcription | transcription |  | 12 |
| ATOX1 | ATX1 antioxidant protein 1 homolog (yeast) | response to stress | copper ion transport |  | 12; 14 |
| ATP10A | ATPase, class V, type 10A | energy metabolism | oxydative phosphorylation |  | 12; 14; 16 |
| ATP5A1 | ATP synthase, H+ transporting, mitochondrial F1 complex, alpha subunit 1, cardiac muscle | energy metabolism | oxydative phosphorylation | adipocyte | 12 |
| ATP8A1 | ATPase, aminophospholipid transporter (APLT), class I, type 8A, member 1 | energy metabolism | oxydative phosphorylation |  | Unpublished |
| AZGP1 | alpha-2-glycoprotein 1, zinc-binding | secreted factor | lipolysis | adipocyte | 12; 15; 16 |
| BAALC | brain and acute leukemia, cytoplasmic | unknown | unknown |  | Unpublished |
| BCAT1 | branched chain aminotransferase 1, cytosolic | amino acid metabolism | amino acid catabolism | macrophage | 12; 15; 16 |
| BCKDHB | branched chain keto acid dehydrogenase E1, beta polypeptide | amino acid metabolism | amino acid catabolism | adipocyte | 12; 15; 16 |
| BTBD7 | BTB (POZ) domain containing 7 | unknown | unknown |  | 14 |
| C1QA | complement component 1, q subcomponent, A chain | immune response | complement system | macrophage | 12; 15; 16 |
| C1QB | complement component 1, q subcomponent, B chain | immune response | complement system | macrophage | 12; 15; 16 |
| C1QC | complement component 1, q subcomponent, C chain | immune response | complement system | macrophage | 12; 15; 16 |
| C2 | complement component 2 | immune response | complement system |  | 12; 16 |
| C3AR1 | complement component 3a receptor 1 | immune response | complement system |  | 15; 16 |
| CARHSP1 | calcium regulated heat stable protein 1, 24kDa | translation | translation |  | 12 |
| CCL18 | chemokine (C-C motif) ligand 18 (pulmonary and activation-regulated) | immune response | inflammatory response | macrophage | 15; 16 |
| CCL19 | chemokine (C-C motif) ligand 19 | immune response | signal transduction |  | 15; 16 |
| CCL2 | chemokine (C-C motif) ligand 2 | immune response | chemotaxis |  | 12; 16 |
| CCL3 | chemokine (C-C motif) ligand 3 | immune response | chemotaxis |  | 15; 16 |
| CCND1 | cyclin D1 | cell cycle | proliferation | adipocyte | 12; 15; 16 |
| CCR1 | chemokine (C-C motif) receptor 1 | immune response | chemotaxis | macrophage | 12; 15; 16 |
| CD14 | CD14 molecule | immune response | response to LPS | macrophage | 12; 15; 16 |
| CD163 | CD163 molecule | immune response | acute phase response | macrophage | 12; 15; 16 |
| CD163L1 | CD163 molecule-like 1 | immune response | signal transduction | macrophage | 12; 15; 16 |
| CD209 | CD209 molecule | immune response | cell adhesion | macrophage | 12; 15; 16 |
| CD48 | CD48 molecule | immune response | immunoglobulin mediated response |  | 15; 16 |
| CD52 | CD52 molecule | signal transduction | transport |  | 12; 16 |
| CD53 | CD53 molecule | signal transduction | transport |  | 12; 16 |
| CD68 | CD68 molecule | immune response | scavenger receptor activity | macrophage | 12; 15; 16 |
| CD74 | CD74 molecule, major histocompatibility complex, class II invariant chain | immune response | antigen presentation |  | 15; 16 |
| CD9 | CD9 molecule | immune response | signal transduction |  | 12; 16 |
| CD97 | CD97 molecule | immune response | leukocyte migration |  | 12; 16 |
| CDK2AP1 | cyclin-dependent kinase 2 associated protein 1 | cell cycle | proliferation |  | 14 |
| CDKN2C | cyclin-dependent kinase inhibitor 2C (p18, inhibits CDK4) | cell cycle | proliferation | adipocyte | 12; 15; 16 |
| CES1 | carboxylesterase 1 (monocyte/macrophage serine esterase 1) | cell differentiation | adipogenesis | adipocyte | 12; 14; 16 |
| CFL1 | cofilin 1 (non-muscle) | cell structure | cytoskeleton organization |  | 14 |
| CIDEA | cell death-inducing DFFA-like effector a | energy metabolism | thermogenesis | adipocyte | 12; 15; 16 |
| CIDEC | cell death-inducing DFFA-like effector c | lipid metabolism | lipogenesis | adipocyte | 12 |
| CIDECP | cell death-inducing DFFA-like effector c pseudogene | unknown | unknown |  | 12; 16 |
| CKB | creatine kinase, brain | energy metabolism | creatin phosphate shuttle |  | 12; 16 |
| CLEC10A | C-type lectin domain family 10, member A | immune response | innate immune response | macrophage | 12; 15; 16 |
| COX7C | cytochrome c oxidase subunit VIIc | energy metabolism | oxydative phosphorylation | adipocyte | 12; 15; 16 |
| CSF1R | colony stimulating factor 1 receptor | immune response | signal transduction | macrophage | 12; 15; 16 |
| CST3 | cystatin C | protein metabolism | tissue remodelling |  | 15; 16 |
| CSTB | cystatin B (stefin B) | protein metabolism | tissue remodelling |  | 12; 16 |
| CTH | cystathionase (cystathionine gamma-lyase) | amino acid metabolism | amino acid synthesis |  | 15; 16 |
| CTSB | cathepsin B | protein metabolism | tissue remodelling | macrophage | 12; 15; 16 |
| CTSC | cathepsin C | protein metabolism | tissue remodelling | macrophage | 12 |
| CTSS | cathepsin S | protein metabolism | tissue remodelling |  | 12; 16 |
| CTSZ | cathepsin Z | protein metabolism | tissue remodelling |  | 12 |
| CYCS | cytochrome c, somatic | energy metabolism | oxydative phosphorylation |  | 12 |
| CYYR1 | cysteine/tyrosine-rich 1 | unknown | unknown |  | Unpublished |
| DCI | dodecenoyl-Coenzyme A delta isomerase (3,2 trans-enoyl-Coenzyme A isomerase) | lipid metabolism | fatty acid oxydation | adipocyte | 12; 15; 16 |
| DGAT1 | diacylglycerol O-acyltransferase homolog 1 (mouse) | lipid metabolism | lipogenesis | adipocyte | 12 |
| DGAT2 | diacylglycerol O-acyltransferase homolog 2 (mouse) | lipid metabolism | lipogenesis | adipocyte | 12 |
| DNAJC13 | DnaJ (Hsp40) homolog, subfamily C, member 13 | unknown | unknown | adipocyte | Unpublished |
| DNASE2 | deoxyribonuclease II, lysosomal | apoptosis | DNA hydrolysis |  | 12; 16 |
| E2F4 | E2F transcription factor 4, p107/p130-binding | transcription | transcription |  | 14 |
| ECHDC1 | enoyl Coenzyme A hydratase domain containing 1 | lipid metabolism | fatty acid oxydation | adipocyte | 12 |
| ECHDC3 | enoyl Coenzyme A hydratase domain containing 3 | lipid metabolism | fatty acid oxydation | adipocyte | 12; 15; 16 |
| EHD4 | EH-domain containing 4 | signal transduction | endocytosis signaling |  | 14; 16 |
| EIF2B1 | eukaryotic translation initiation factor 2B, subunit 1 alpha, 26kDa | translation | translation |  | Unpublished |
| EIF4A1 | eukaryotic translation initiation factor 4A1 | translation | translation | macrophage | 14; 15; 16 |
| ELOVL5 | ELOVL family member 5, elongation of long chain fatty acids | lipid metabolism | lipogenesis | adipocyte | 12; 15; 16 |
| EMILIN2 | elastin microfibril interfacer 2 | cell adhesion | tissue remodelling | macrophage | 12; 15; 16 |
| EN2 | engrailed homeobox 2 | transcription | transcription |  | Unpublished |
| ENO1 | enolase 1, (alpha) | carbohydrate metabolism | aerobic glycolysis |  | 12; 16 |
| ENO3 | enolase 3 (beta, muscle) | carbohydrate metabolism | aerobic glycolysis | adipocyte | Unpublished |
| ETFA | electron-transfer-flavoprotein, alpha polypeptide | energy metabolism | fatty acid oxidation | adipocyte | 15; 16 |
| ETFDH | electron-transferring-flavoprotein dehydrogenase | energy metabolism | fatty acid oxidation | adipocyte | 12; 15; 16 |
| EXOC1 | exocyst complex component 1 | transport | transport |  | 14 |
| FABP4 | fatty acid binding protein 4, adipocyte | lipid metabolism | transport | adipocyte | 12; 15; 16 |
| FADS1 | fatty acid desaturase 1 | lipid metabolism | lipogenesis | adipocyte | 12; 15; 16 |
| FADS2 | fatty acid desaturase 2 | lipid metabolism | lipogenesis | adipocyte | 12 |
| FASN | fatty acid synthase | lipid metabolism | lipogenesis | adipocyte | 12; 15; 16 |
| FBP1 | fructose-1,6-bisphosphatase 1 | carbohydrate metabolism | aerobic glycolysis |  | 12; 16 |
| FCER1G | Fc fragment of IgE, high affinity I, receptor for; gamma polypeptide | immune response | immunoglobulin mediated response |  | 12 |
| FCGBP | Fc fragment of IgG binding protein | immune response | immunoglobulin mediated response | macrophage | 12 |
| FCGR2B | Fc fragment of IgG, low affinity IIb, receptor (CD32) | immune response | signal transduction | macrophage | 12; 15; 16 |
| FCGRT | Fc fragment of IgG, receptor, transporter, alpha | immune response | antigen presentation | macrophage | 15; 16 |
| FGF2 | fibroblast growth factor 2 (basic) | secreted factor | cell proliferation | adipocyte | 15; 16 |
| FN1 | fibronectin 1 | immune response | cell adhesion |  | 12; 14; 16 |
| FSTL1 | follistatin-like 1 | secreted factor | cell proliferation |  | 15; 16 |
| FTH1 | ferritin, heavy polypeptide 1 | immune response | iron homeostasis |  | 12; 16 |
| FTL | ferritin, light polypeptide | immune response | iron homeostasis |  | 14; 16 |
| FUZ | fuzzy homolog (Drosophila) | cell structure | cytoskeleton organization |  | 15; 16 |
| GAPDH | glyceraldehyde-3-phosphate dehydrogenase | carbohydrate metabolism | aerobic glycolysis |  | 12 |
| GATM | glycine amidinotransferase (L-arginine:glycine amidinotransferase) | energy metabolism | creatin biosynthesis | macrophage | 12 |
| GBA | glucosidase, beta, acid | carbohydrate metabolism | glycogenolysis |  | 15; 16 |
| GHR | growth hormone receptor | signal transduction | lipolysis | adipocyte | 15; 16 |
| GIT2 | G protein-coupled receptor kinase interacting ArfGAP 2 | signal transduction | cytoskeleton organization |  | 14; 16 |
| GPD1L | glycerol-3-phosphate dehydrogenase 1-like | lipid metabolism | glycerophospholipid metabolism |  | 12; 16 |
| GPR109A | G protein-coupled receptor 109A | lipid metabolism | lipolysis |  | Unpublished |
| GPT | glutamic-pyruvate transaminase (alanine aminotransferase) | amino acid metabolism | amino acid metabolism | adipocyte | 12; 15; 16 |
| GPT2 | glutamic pyruvate transaminase (alanine aminotransferase) 2 | amino acid metabolism | amino acid metabolism | adipocyte | 12; 15; 16 |
| GYS1 | glycogen synthase 1 (muscle) | carbohydrate metabolism | glycogenesis | adipocyte | 12 |
| HADH | Hydroxyacyl-Coenzyme A dehydrogenase | lipid metabolism | fatty acid oxidation | adipocyte | 15; 16 |
| HK1 | hexokinase 1 | carbohydrate metabolism | aerobic glycolysis |  | Unpublished |
| HLA-A | major histocompatibility complex, class I, A | immune response | antigen presentation |  | 15; 16 |
| HMOX1 | heme oxygenase (decycling) 1 | immune response | hemoglobin degradation |  | 12; 16 |
| HP | haptoglobin | immune response | hemoglobin degradation | adipocyte | 12 |
| HSDL2 | hydroxysteroid dehydrogenase like 2 | lipid metabolism | lipolysis | adipocyte | 15; 16 |
| IDH1 | isocitrate dehydrogenase 1 (NADP+), soluble | energy metabolism | tricarboxylic acid cycle | adipocyte | 12; 15; 16 |
| IFI30 | interferon, gamma-inducible protein 30 | immune response | antigen presentation |  | 12; 16 |
| IGF1 | insulin-like growth factor 1 (somatomedin C) | secreted factor | cell proliferation | adipocyte | 12; 15; 16 |
| IL10 | interleukin 10 | immune response | inflammatory cytokine | macrophage | 12; 15; 16 |
| IL10RA | interleukin 10 receptor, alpha | immune response | inflammatory response |  | 12; 16 |
| IL1RN | interleukin 1 receptor antagonist | immune response | inflammatory response |  | 12 |
| IL4R | interleukin 4 receptor | immune response | inflammatory response |  | 15; 16 |
| INHBB | inhibin, beta B | secreted factor | energy balance |  | 15; 16 |
| IRF5 | interferon regulatory factor 5 | transcription | transcription | macrophage | 12; 15; 16 |
| IRS1 | insulin receptor substrate 1 | signal transduction | insulin signaling | adipocyte | 12; 15; 16 |
| IRS2 | insulin receptor substrate 2 | signal transduction | insulin signaling | adipocyte | 15; 16 |
| IRX1 | iroquois homeobox 1 | transcription | transcription |  | Unpublished |
| ITGAM | integrin, alpha M (complement component 3 receptor 3 subunit) | immune response | cell adhesion |  | 12; 16 |
| ITGAX | integrin, alpha X (complement component 3 receptor 4 subunit) | immune response | cell adhesion |  | 12; 16 |
| ITGB2 | integrin, beta 2 (complement component 3 receptor 3 and 4 subunit) | immune response | cell adhesion |  | 12; 16 |
| ITGB5 | integrin, beta 5 | immune response | cell adhesion |  | 12; 16 |
| KIT | v-kit Hardy-Zuckerman 4 feline sarcoma viral oncogene homolog | immune response | signal transduction |  | 15; 16 |
| KYNU | kynureninase (L-kynurenine hydrolase) | amino acid metabolism | amino acid catabolism | macrophage | 12 |
| LAPTM5 | lysosomal protein transmembrane 5 | transport | transport |  | 12; 15; 16 |
| LASS2 | LAG1 homolog, ceramide synthase 2 | lipid metabolism | sphingolipid metabolism |  | 12; 14; 16 |
| LCP1 | lymphocyte cytosolic protein 1 (L-plastin) | immune response | immunoglobulin mediated response |  | 12; 14 |
| LDHA | lactate dehydrogenase A | carbohydrate metabolism | anaerobic glycolysis | adipocyte | 12; 14; 16 |
| LDLR | S100 calcium binding protein A4 | immune response | inflammatory response |  | 12; 16 |
| LDLR | low density lipoprotein receptor | lipid metabolism | cholesterol metabolism | adipocyte | 12 |
| LEP | leptin | secreted factor | energy balance | adipocyte | 15; 16 |
| LGR4 | leucine-rich repeat-containing G protein-coupled receptor 4 | signal transduction | signal transduction |  | 15; 16 |
| LILRA6 | leukocyte immunoglobulin-like receptor, subfamily A (with TM domain), member 6 | immune response | antigen presentation |  | 12 |
| LILRB3 | leukocyte immunoglobulin-like receptor, subfamily B (with TM and ITIM domains), member 3 | immune response | inflammatory response |  | 15; 16 |
| LIPA | lipase A, lysosomal acid, cholesterol esterase | lipid metabolism | cholesterol metabolism | macrophage | 12; 15; 16 |
| LIPE | lipase, hormone-sensitive | lipid metabolism | lipolysis |  | Unpublished |
| LOX | lysyl oxidase | cell adhesion | tissue remodelling |  | 12; 16 |
| LOXL1 | lysyl oxidase-like 1 | cell adhesion | tissue remodelling |  | 15; 16 |
| LOXL2 | lysyl oxidase-like 2 | cell adhesion | tissue remodelling | adipocyte | 12; 15; 16 |
| LPCAT1 | lysophosphatidylcholine acyltransferase 1 | lipid metabolism | phospholipid synthesis |  | Unpublished |
| LPIN1 | lipin 1 | lipid metabolism | lipolysis |  | 12; 16 |
| LY86 | lymphocyte antigen 86 | immune response | inflammatory response | macrophage | 12; 15; 16 |
| LY96 | lymphocyte antigen 96 | immune response | inflammatory response |  | 12; 16 |
| MAOA | monoamine oxidase A | signal transduction | dopamin catabolism |  | 15; 16 |
| MAPK3 | mitogen-activated protein kinase 3 | signal transduction | cell proliferation |  | 14 |
| MARCO | macrophage receptor with collagenous structure | immune response | innate immune response | macrophage | 12; 15; 16 |
| MCM3 | minichromosome maintenance complex component 3 | transcription | transcription |  | 14 |
| MDH2 | malate dehydrogenase 2, NAD (mitochondrial) | energy metabolism | tricarboxylic acid cycle |  | 15; 16 |
| ME1 | malic enzyme 1, NADP(+)-dependent, cytosolic | energy metabolism | tricarboxylic acid cycle |  | 12 |
| MECR | mitochondrial trans-2-enoyl-CoA reductase | lipid metabolism | fatty acid oxydation |  | 12; 14 |
| MEST | mesoderm specific transcript homolog (mouse) | cell differentiation | adipogenesis | adipocyte | 12; 14 |
| MMP19 | matrix metallopeptidase 19 | protein metabolism | tissue remodelling |  | 12; 16 |
| MMP9 | matrix metallopeptidase 9 (gelatinase B, 92kDa gelatinase, 92kDa type IV collagenase) | protein metabolism | tissue remodelling |  | 12; 16 |
| MNDA | myeloid cell nuclear differentiation antigen | immune response | response to interferon |  | 15; 16 |
| MRC1L1 | mannose receptor, C type 1-like 1 | immune response | signal transduction | macrophage | 12; 15; 16 |
| MS4A4A | membrane-spanning 4-domains, subfamily A, member 4 | immune response | signal transduction | macrophage | 12; 15; 16 |
| MS4A6A | membrane-spanning 4-domains, subfamily A, member 6A | immune response | signal transduction | macrophage | 12; 15; 16 |
| MS4A7 | membrane-spanning 4-domains, subfamily A, member 7 | immune response | signal transduction | macrophage | 12; 15; 16 |
| MSH6 | mutS homolog 6 (E. coli) | transcription | transcription | macrophage | 14; 15; 16 |
| MT1E | metallothionein 1E | response to stress | response to oxidative stress |  | 12; 16 |
| MT1G | metallothionein 1G | response to stress | response to oxidative stress |  | 12 |
| MYD88 | myeloid differentiation primary response gene (88) | immune response | inflammatory response |  | 12; 16 |
| NDUFA9 | NADH dehydrogenase (ubiquinone) 1 alpha subcomplex, 9, 39kDa | energy metabolism | oxydative phosphorylation |  | 14 |
| NDUFB8 | NADH dehydrogenase (ubiquinone) 1 beta subcomplex, 8, 19kDa | energy metabolism | oxydative phosphorylation |  | 15; 16 |
| NEUROG3 | neurogenin 3 | transcription | transcription |  | Unpublished |
| NFKB2 | nuclear factor of kappa light polypeptide gene enhancer in B-cells 2 (p49/p100) | immune response | signal transduction |  | Unpublished |
| NOMO1 | NODAL modulator 1 | unknown | unknown |  | 12; 14 |
| NPAS3 | neuronal PAS domain protein 3 | transcription | transcription |  | Unpublished |
| NRIP1 | nuclear receptor interacting protein 1 | transcription | transcription | adipocyte | 12; 15; 16 |
| NUP62 | nucleoporin 62kDa | signal transduction | transport | macrophage | 14; 15; 16 |
| OSBPL9 | oxysterol binding protein-like 9 | lipid metabolism | cholesterol transport |  | Unpublished |
| OXSR1 | oxidative-stress responsive 1 | response to stress | response to oxidative stress |  | Unpublished |
| PC | pyruvate carboxylase | carbohydrate metabolism | gluconeogenesis | adipocyte | 12; 15; 16 |
| PCCA | propionyl Coenzyme A carboxylase, alpha polypeptide | lipid metabolism | fatty acid oxidation | adipocyte | 15; 16 |
| PCK1 | phosphoenolpyruvate carboxykinase 1 (soluble) | carbohydrate metabolism | aerobic glycolysis | adipocyte | 15; 16 |
| PCK2 | phosphoenolpyruvate carboxykinase 2 (mitochondrial) | carbohydrate metabolism | aerobic glycolysis |  | 12; 16 |
| PDHA1 | pyruvate dehydrogenase (lipoamide) alpha 1 | carbohydrate metabolism | aerobic glycolysis | adipocyte | 15; 16 |
| PECI | peroxisomal D3,D2-enoyl-CoA isomerase | lipid metabolism | fatty acid oxidation | adipocyte | 15; 16 |
| PECR | peroxisomal trans-2-enoyl-CoA reductase | lipid metabolism | fatty acid biosynthesis |  | 12; 16 |
| PELP1 | proline, glutamate and leucine rich protein 1 | transcription | transcription |  | Unpublished |
| PEX11A | peroxisomal biogenesis factor 11 alpha | signal transduction | cell differentiation | adipocyte | 15; 16 |
| PFKFB1 | 6-phosphofructo-2-kinase/fructose-2,6-biphosphatase 1 | carbohydrate metabolism | aerobic glycolysis | adipocyte | 12; 15; 16 |
| PFKM | phosphofructokinase, muscle | carbohydrate metabolism | aerobic glycolysis |  | Unpublished |
| PGAM1 | phosphoglycerate mutase 1 (brain) | carbohydrate metabolism | aerobic glycolysis |  | 12 |
| PGDS | prostaglandin-H2 D-isomerase | signal transduction | prostaglandin metabolism |  | 12; 16 |
| PGK1 | phosphoglycerate kinase 1 | carbohydrate metabolism | aerobic glycolysis |  | 12 |
| PGM1 | phosphoglucomutase 1 | carbohydrate metabolism | aerobic glycolysis | adipocyte | 12 |
| PHYH | phytanoyl-CoA 2-hydroxylase | lipid metabolism | fatty acid oxidation | adipocyte | 15; 16 |
| PKIG | protein kinase (cAMP-dependent, catalytic) inhibitor gamma | lipid metabolism | lipolysis |  | 14 |
| PKM2 | pyruvate kinase, muscle | carbohydrate metabolism | aerobic glycolysis |  | 12; 16 |
| PLA2G7 | phospholipase A2, group VII (platelet-activating factor acetylhydrolase, plasma) | immune response | inflammatory response | macrophage | 12; 15; 16 |
| PLAU | plasminogen activator, urokinase | cell adhesion | tissue remodelling |  | 12; 16 |
| PLAUR | plasminogen activator, urokinase receptor | signal transduction | proteolysis |  | 15; 16 |
| PNPLA2 | patatin-like phospholipase domain containing 2 | lipid metabolism | lipolysis |  | 12 |
| PNPLA3 | patatin-like phospholipase domain containing 3 | lipid metabolism | lipolysis | adipocyte | Unpublished |
| POP4 | processing of precursor 4, ribonuclease P/MRP subunit (S. cerevisiae) | transport | RNA transport | adipocyte | 12 |
| PRDX6 | peroxiredoxin 6 | response to stress | response to oxidative stress | adipocyte | 15; 16 |
| PRKAR2B | protein kinase, cAMP-dependent, regulatory, type II, beta | signal transduction | signal transduction | adipocyte | 15; 16 |
| PSMC4 | proteasome (prosome, macropain) 26S subunit, ATPase, 4 | protein metabolism | proteolysis |  | 15; 16 |
| PWP1 | PWP1 homolog (S. cerevisiae) | transcription | transcription | adipocyte | 14 |
| RAC1 | ras-related C3 botulinum toxin substrate 1 (rho family, small GTP binding protein Rac1) | immune response | signal transduction |  | Unpublished |
| RDH10 | retinol dehydrogenase 10 (all-trans) | lipid metabolism | retinol metabolism | adipocyte | 15; 16 |
| RNF5 | ring finger protein 5 | protein metabolism | proteolysis |  | 14 |
| RNH1 | ribonuclease/angiogenin inhibitor 1 | transcription | transcription |  | 12; 16 |
| ROBO3 | roundabout, axon guidance receptor, homolog 3 (Drosophila) | signal transduction | cell differenciation |  | 12 |
| RPL6 | ribosomal protein L6 | translation | translation |  | 14; 16 |
| RPN1 | ribophorin I | protein metabolism | protein glycosylation |  | 14 |
| SAA4 | serum amyloid A4, constitutive | immune response | acute phase response | adipocyte | 12 |
| SCARA5 | scavenger receptor class A, member 5 (putative) | immune response | iron transport |  | 15; 16 |
| SCD | stearoyl-CoA desaturase (delta-9-desaturase) | lipid metabolism | lipogenesis | adipocyte | 12 |
| SFRP2 | secreted frizzled-related protein 2 | cell differentiation | adipogenesis |  | 12; 16 |
| SH3BGRL | SH3 domain binding glutamic acid-rich protein like | unknown | unknown |  | 14; 16 |
| SIRT1 | sirtuin (silent mating type information regulation 2 homolog) 1 (S. cerevisiae) | translation | histone deacetylase |  | 15; 16 |
| SLC19A2 | solute carrier family 19 (thiamine transporter), member 2 | transport | thiamine transport | adipocyte | 15; 16 |
| SLC19A3 | solute carrier family 19, member 3 | transport | thiamine transport | adipocyte | 15; 16 |
| SLC2A4 | solute carrier family 2 (facilitated glucose transporter), member 4 | carbohydrate metabolism | glucose transport | adipocyte | 12; 15; 16 |
| SLC35C2 | solute carrier family 35, member C2 | transport | response to hypoxia |  | Unpublished |
| SLC4A4 | solute carrier family 4, sodium bicarbonate cotransporter, member 4 | transport | sodium-bicarbonate transport | adipocyte | 15; 16 |
| SNCA | synuclein, alpha (non A4 component of amyloid precursor) | immune response | microglial cell activation | macrophage | 15; 16 |
| SNTB2 | syntrophin, beta 2 (dystrophin-associated protein A1, 59kDa, basic component 2) | cell structure | cytoskeleton organization |  | 12; 14 |
| SPP1 | secreted phosphoprotein 1 | immune response | chemotaxis | macrophage | 12; 15; 16 |
| SPTAN1 | spectrin, alpha, non-erythrocytic 1 (alpha-fodrin) | cell structure | cytoskeleton organization |  | 14; 16 |
| SREBF1 | sterol regulatory element binding transcription factor 1 | transcription | transcription | adipocyte | 12 |
| SRP9 | signal recognition particle 9kDa | translation | translation |  | 14 |
| TCEAL8 | transcription elongation factor A (SII)-like 8 | transcription | transcription |  | Unpublished |
| TDRD7 | tudor domain containing 7 | translation | translation |  | 12; 14 |
| TFRC | transferrin receptor (p90, CD71) | immune response | iron homeostasis | macrophage | 12; 15; 16 |
| THBS4 | thrombospondin 4 | immune response | cell adhesion |  | 12 |
| THRSP | thyroid hormone responsive (SPOT14 homolog, rat) | lipid metabolism | lipogenesis | adipocyte | 12 |
| TMEM135 | transmembrane protein 135 | unknown | unknown | adipocyte | 12; 15; 16 |
| TNRC6B | trinucleotide repeat containing 6B | translation | translation |  | 14 |
| TPI1 | triosephosphate isomerase 1 | carbohydrate metabolism | aerobic glycolysis |  | Unpublished |
| TPM3 | tropomyosin 3 | cell structure | cytoskeleton organization |  | 12; 14 |
| TPST2 | tyrosylprotein sulfotransferase 2 | protein metabolism | peptidyl-tyrosine sulfation |  | 14; 16 |
| TSEN54 | tRNA splicing endonuclease 54 homolog (S. cerevisiae) | translation | translation |  | Unpublished |
| TUBA1A | tubulin, alpha 1a | transport | transport |  | 12; 16 |
| TWIST1 | twist homolog 1 (Drosophila) | transcription | transcription |  | 12; 16 |
| TXNDC5 | thioredoxin domain containing 5 (endoplasmic reticulum) | transport | transport |  | 12 |
| UCN | urocortin | apoptosis | secreted factor |  | Unpublished |
| UQCRC2 | ubiquinol-cytochrome c reductase core protein II | energy metabolism | oxydative phosphorylation | adipocyte | 12; 15; 16 |
| VEGFA | vascular endothelial growth factor A | secreted factor | angiogenesis |  | 15; 16 |
| VGLL3 | vestigial like 3 (Drosophila) | transcription | transcription | adipocyte | 14 |
| WDR1 | WD repeat domain 1 | cell structure | cytoskeleton organization |  | 12; 14 |
| WISP2 | WNT1 inducible signaling pathway protein 2 | cell differentiation | adipogenesis |  | 15; 16 |
| 18S | eukaryotic ribosomal protein 18S | normalization | normalization |  | Normalization |
| GUSB | glucuronidase, beta | normalization | normalization |  | Normalization |
