# Supplementary material for: Determinants of Human Adipose Tissue Gene Expression: Impact of Diet, Sex, Metabolic Status, and Cis Genetic Regulation
Source: PLoS Genet. 2012 Sep 27;8(9):e1002959. doi: 10.1371/journal.pgen.1002959 (PMC3459935; doi:10.1371/journal.pgen.1002959)
Supplement: Table S4 — Genes with opposite regulation during low calorie diet and weight maintenance diet phases. A linear mixed effect model was run separately for men and women with time point (clinical investigation day, CID), weight at baseline and weight change as fixed effect. Centre and subject were entered as random effect. Diet was included as supplementary fixed effect in the model investigating genes during weight maintenance diet. The regressions equations tested without and with weight are displayed below: Y is the log2 expression value for gene i, in subject l, and centre k. The random term ε represents the random error that was assumed to be normally distributed. The Tukey HSD was used as post-hoc test. The Benjamini-Hochberg procedure was used to control for multiple testing. Only significant genes common to men and women and weight-independent genes were selected. *: Values refer to median mRNA level fold change from 311 subjects (107 men, 204 women). (DOCX) [file pgen.1002959.s009.docx]

**Table S4 Genes with opposite regulation during low calorie diet and weight maintenance diet phases**

|  | **Low Calorie Diet** | | |  | **Weight Maintenance Diet** | | |
| --- | --- | --- | --- | --- | --- | --- | --- |
| **Gene Symbol** | **Fold Change*** | **P-value Men^$^** | **P-value Women^$^** |  | **Fold Change*** | **P-value Men^£^** | **P-value Women^£^** |
| *SPP1* | 1.39 | 0.041310 | 0.000014 |  | 0.44 | 0.000000 | 0.000000 |
| *PLA2G7* | 1.38 | 0.002158 | 0.000000 |  | 0.51 | 0.000000 | 0.000000 |
| *IL1RN* | 1.27 | 0.008192 | 0.000000 |  | 0.54 | 0.000000 | 0.000000 |
| *AADACL1* | 1.26 | 0.004215 | 0.000002 |  | 0.60 | 0.000000 | 0.000000 |
| *CD52* | 1.26 | 0.000380 | 0.000000 |  | 0.68 | 0.000004 | 0.000000 |
| *IFI30* | 1.26 | 0.000085 | 0.000000 |  | 0.65 | 0.000000 | 0.000000 |
| *FBP1* | 1.24 | 0.000002 | 0.000000 |  | 0.64 | 0.000000 | 0.000000 |
| *CCL18* | 1.18 | 0.001200 | 0.009318 |  | 0.72 | 0.002395 | 0.000000 |
| *CD68* | 1.17 | 0.000001 | 0.000000 |  | 0.70 | 0.000000 | 0.000000 |
| *LIPA* | 1.17 | 0.000022 | 0.000000 |  | 0.69 | 0.000000 | 0.000000 |
| *LAPTM5* | 1.13 | 0.019466 | 0.000036 |  | 0.81 | 0.006775 | 0.000033 |
| *ATF3* | 1.12 | 0.036286 | 0.000026 |  | 0.70 | 0.000000 | 0.000000 |
| *IL10* | 1.12 | 0.000577 | 0.000134 |  | 0.70 | 0.000000 | 0.000000 |
| *LY86* | 1.12 | 0.000011 | 0.000310 |  | 0.83 | 0.000011 | 0.000763 |
| *ITGB2* | 1.08 | 0.025433 | 0.000353 |  | 0.84 | 0.011168 | 0.000840 |
| *C3AR1* | 1.07 | 0.031779 | 0.007469 |  | 0.78 | 0.000037 | 0.008713 |
| *MS4A7* | 1.06 | 0.003025 | 0.006431 |  | 0.83 | 0.000012 | 0.000169 |
| *SIRT1* | 0.96 | 0.037797 | 0.000908 |  | 1.15 | 0.000000 | 0.000002 |
| *EXOC1* | 0.93 | 0.028874 | 0.000881 |  | 1.15 | 0.000000 | 0.000004 |
| *NFKB2* | 0.93 | 0.012445 | 0.040417 |  | 1.19 | 0.000000 | 0.000000 |
| *SLC35C2* | 0.93 | 0.011629 | 0.000006 |  | 1.15 | 0.000000 | 0.000000 |
| *RAC1* | 0.92 | 0.004747 | 0.000062 |  | 1.14 | 0.000000 | 0.000000 |
| *TDRD7* | 0.92 | 0.000427 | 0.000001 |  | 1.12 | 0.000000 | 0.000290 |
| *ACTR3* | 0.91 | 0.016830 | 0.000001 |  | 1.11 | 0.000000 | 0.000012 |
| *FTH1* | 0.91 | 0.002635 | 0.014657 |  | 1.05 | 0.000056 | 0.040772 |
| *SH3BGRL* | 0.91 | 0.018513 | 0.001613 |  | 1.11 | 0.000007 | 0.000936 |
| *WDR1* | 0.91 | 0.001990 | 0.001325 |  | 1.14 | 0.000000 | 0.000001 |
| *BTBD7* | 0.90 | 0.009699 | 0.000000 |  | 1.25 | 0.000000 | 0.000000 |
| *ETFA* | 0.90 | 0.000015 | 0.000000 |  | 1.13 | 0.000000 | 0.000002 |
| *LASS2* | 0.90 | 0.005997 | 0.000000 |  | 1.13 | 0.000000 | 0.000000 |
| *NUP62* | 0.90 | 0.014863 | 0.000855 |  | 1.14 | 0.000000 | 0.002343 |
| *OXSR1* | 0.90 | 0.002752 | 0.000004 |  | 1.13 | 0.000157 | 0.000004 |
| *PKIG* | 0.90 | 0.006673 | 0.000000 |  | 1.20 | 0.000000 | 0.000002 |
| *ATP8A1* | 0.89 | 0.000668 | 0.000014 |  | 1.21 | 0.000001 | 0.000063 |
| *HK1* | 0.89 | 0.000050 | 0.000000 |  | 1.21 | 0.000000 | 0.000000 |
| *HLA.A* | 0.89 | 0.010602 | 0.000017 |  | 1.23 | 0.000000 | 0.000000 |
| *TXNDC5* | 0.89 | 0.014563 | 0.005656 |  | 1.05 | 0.015490 | 0.002564 |
| *ACACB* | 0.88 | 0.017544 | 0.000000 |  | 1.39 | 0.000000 | 0.000000 |
| *EIF4A1* | 0.88 | 0.000620 | 0.000000 |  | 1.13 | 0.000000 | 0.000000 |
| *IL4R* | 0.88 | 0.036286 | 0.006037 |  | 1.24 | 0.000000 | 0.000000 |
| *PELP1* | 0.88 | 0.000020 | 0.000000 |  | 1.19 | 0.000000 | 0.000000 |
| *SRP9* | 0.88 | 0.011228 | 0.000002 |  | 1.19 | 0.000000 | 0.000001 |
| *TCEAL8* | 0.88 | 0.000227 | 0.000000 |  | 1.14 | 0.000000 | 0.000000 |
| *AGPAT1* | 0.87 | 0.001239 | 0.000000 |  | 1.27 | 0.000000 | 0.000000 |
| *GIT2* | 0.87 | 0.003887 | 0.000000 |  | 1.26 | 0.000000 | 0.000000 |
| *LPCAT1* | 0.87 | 0.017370 | 0.000001 |  | 1.23 | 0.000000 | 0.000000 |
| *PHYH* | 0.87 | 0.000155 | 0.000000 |  | 1.11 | 0.000009 | 0.000037 |
| *RNH1* | 0.87 | 0.002110 | 0.000000 |  | 1.10 | 0.002207 | 0.037132 |
| *SCARA5* | 0.87 | 0.000387 | 0.000000 |  | 1.15 | 0.000000 | 0.000311 |
| *COX7C* | 0.86 | 0.001328 | 0.000000 |  | 1.20 | 0.000000 | 0.000000 |
| *HADH* | 0.86 | 0.000050 | 0.000000 |  | 1.19 | 0.000000 | 0.000000 |
| *KIT* | 0.86 | 0.003705 | 0.000000 |  | 1.22 | 0.000000 | 0.000000 |
| *MAPK3* | 0.86 | 0.000085 | 0.000000 |  | 1.20 | 0.000000 | 0.000000 |
| *NDUFB8* | 0.86 | 0.000091 | 0.000000 |  | 1.14 | 0.000000 | 0.000000 |
| *OSBPL9* | 0.86 | 0.015462 | 0.000516 |  | 1.14 | 0.000000 | 0.000005 |
| *ADHFE1* | 0.85 | 0.000003 | 0.000000 |  | 1.30 | 0.000000 | 0.000000 |
| *ARF3* | 0.85 | 0.000083 | 0.000000 |  | 1.22 | 0.000000 | 0.000000 |
| *DCI* | 0.85 | 0.000328 | 0.000000 |  | 1.21 | 0.000000 | 0.000000 |
| *GHR* | 0.85 | 0.000010 | 0.000000 |  | 1.21 | 0.000000 | 0.000229 |
| *MCM3* | 0.85 | 0.000002 | 0.000000 |  | 1.24 | 0.000000 | 0.000000 |
| *PCK1* | 0.85 | 0.016621 | 0.000000 |  | 1.18 | 0.009436 | 0.000025 |
| *PECI* | 0.85 | 0.011642 | 0.000060 |  | 1.16 | 0.000150 | 0.000000 |
| *TUBA1A* | 0.85 | 0.000015 | 0.000000 |  | 1.16 | 0.000000 | 0.000015 |
| *ALDH6A1* | 0.84 | 0.028755 | 0.000000 |  | 1.35 | 0.000000 | 0.000000 |
| *DNAJC13* | 0.84 | 0.002809 | 0.000000 |  | 1.20 | 0.000000 | 0.000000 |
| *ECHDC3* | 0.84 | 0.002596 | 0.000000 |  | 1.20 | 0.000001 | 0.000120 |
| *EIF2B1* | 0.84 | 0.000560 | 0.000000 |  | 1.23 | 0.000000 | 0.000000 |
| *ENO3* | 0.84 | 0.000675 | 0.000000 |  | 1.44 | 0.000000 | 0.000132 |
| *MAOA* | 0.84 | 0.000001 | 0.000000 |  | 1.23 | 0.000000 | 0.000000 |
| *PCCA* | 0.84 | 0.000964 | 0.000000 |  | 1.16 | 0.000000 | 0.000000 |
| *PSMC4* | 0.84 | 0.014314 | 0.000000 |  | 1.17 | 0.000000 | 0.000000 |
| *PWP1* | 0.84 | 0.000010 | 0.000000 |  | 1.14 | 0.000000 | 0.000000 |
| *ARPC1A* | 0.83 | 0.000022 | 0.000004 |  | 1.24 | 0.000000 | 0.000245 |
| *E2F4* | 0.83 | 0.000007 | 0.000000 |  | 1.29 | 0.000000 | 0.000000 |
| *HSDL2* | 0.83 | 0.000001 | 0.000000 |  | 1.16 | 0.000000 | 0.000029 |
| *LGR4* | 0.83 | 0.000241 | 0.000000 |  | 1.20 | 0.000000 | 0.000000 |
| *RPN1* | 0.83 | 0.000239 | 0.000000 |  | 1.12 | 0.000001 | 0.000000 |
| *TPI1* | 0.83 | 0.000175 | 0.000000 |  | 1.16 | 0.000041 | 0.000000 |
| *ACAT1* | 0.82 | 0.000085 | 0.000000 |  | 1.23 | 0.000000 | 0.000000 |
| *FGF2* | 0.82 | 0.000003 | 0.000000 |  | 1.29 | 0.000000 | 0.000000 |
| *GPR109A* | 0.82 | 0.006778 | 0.000000 |  | 1.19 | 0.000000 | 0.000000 |
| *PKM2* | 0.82 | 0.000025 | 0.000000 |  | 1.10 | 0.000056 | 0.000000 |
| *CARHSP1* | 0.81 | 0.007104 | 0.000000 |  | 1.34 | 0.000000 | 0.000016 |
| *CYYR1* | 0.81 | 0.000007 | 0.000000 |  | 1.37 | 0.000000 | 0.000000 |
| *IRS2* | 0.81 | 0.000008 | 0.000000 |  | 1.39 | 0.000000 | 0.000000 |
| *PFKM* | 0.81 | 0.000003 | 0.000000 |  | 1.26 | 0.000000 | 0.000000 |
| *AQP7* | 0.80 | 0.000006 | 0.000000 |  | 1.28 | 0.000000 | 0.000000 |
| *BCKDHB* | 0.80 | 0.000003 | 0.000000 |  | 1.40 | 0.000000 | 0.000000 |
| *CFL1* | 0.80 | 0.000508 | 0.000000 |  | 1.17 | 0.000000 | 0.000922 |
| *CKB* | 0.80 | 0.000175 | 0.000000 |  | 1.45 | 0.000000 | 0.000000 |
| *EHD4* | 0.80 | 0.000001 | 0.000000 |  | 1.27 | 0.000000 | 0.000000 |
| *MDH2* | 0.80 | 0.000000 | 0.000000 |  | 1.24 | 0.000000 | 0.000000 |
| *NDUFA9* | 0.80 | 0.000002 | 0.000000 |  | 1.16 | 0.000003 | 0.000000 |
| *PC* | 0.80 | 0.002561 | 0.000000 |  | 1.32 | 0.000000 | 0.000000 |
| *SNTB2* | 0.80 | 0.000113 | 0.000000 |  | 1.29 | 0.000000 | 0.000000 |
| *EN2* | 0.79 | 0.033071 | 0.000000 |  | 1.19 | 0.000294 | 0.000010 |
| *FN1* | 0.79 | 0.000001 | 0.000000 |  | 1.16 | 0.000000 | 0.000037 |
| *LPIN1* | 0.79 | 0.000018 | 0.000000 |  | 1.46 | 0.000000 | 0.000000 |
| *POP4* | 0.79 | 0.000020 | 0.000000 |  | 1.17 | 0.000000 | 0.000000 |
| *SLC4A4* | 0.79 | 0.002636 | 0.000000 |  | 1.24 | 0.000001 | 0.000001 |
| *TPM3* | 0.79 | 0.004969 | 0.000000 |  | 1.14 | 0.000027 | 0.000163 |
| *AES* | 0.78 | 0.000016 | 0.000000 |  | 1.26 | 0.000000 | 0.000000 |
| *AP2M1* | 0.78 | 0.000002 | 0.000000 |  | 1.26 | 0.000000 | 0.000000 |
| *ATP5A1* | 0.78 | 0.000000 | 0.000000 |  | 1.28 | 0.000000 | 0.000000 |
| *NOMO1* | 0.78 | 0.000000 | 0.000000 |  | 1.19 | 0.000000 | 0.000000 |
| *CIDECP* | 0.77 | 0.000000 | 0.000000 |  | 1.30 | 0.000000 | 0.000000 |
| *IGF1* | 0.77 | 0.004643 | 0.000000 |  | 1.37 | 0.000000 | 0.000000 |
| *LOXL1* | 0.77 | 0.008584 | 0.000000 |  | 1.20 | 0.000001 | 0.000000 |
| *PEX11A* | 0.77 | 0.002237 | 0.000000 |  | 1.16 | 0.000130 | 0.000001 |
| *PGK1* | 0.77 | 0.000003 | 0.000000 |  | 1.21 | 0.000000 | 0.000000 |
| *PNPLA2* | 0.77 | 0.000002 | 0.000000 |  | 1.27 | 0.000000 | 0.000000 |
| *SLC2A4* | 0.77 | 0.002161 | 0.000000 |  | 1.28 | 0.000000 | 0.000000 |
| *DNASE2* | 0.76 | 0.000010 | 0.000000 |  | 1.23 | 0.000000 | 0.000000 |
| *ITGB5* | 0.76 | 0.000000 | 0.000000 |  | 1.08 | 0.000009 | 0.019188 |
| *ETFDH* | 0.75 | 0.000000 | 0.000000 |  | 1.28 | 0.000000 | 0.000000 |
| *FSTL1* | 0.75 | 0.000000 | 0.000000 |  | 1.20 | 0.000000 | 0.000000 |
| *IRS1* | 0.75 | 0.000084 | 0.000000 |  | 1.50 | 0.000000 | 0.000000 |
| *MEST* | 0.75 | 0.000120 | 0.000000 |  | 1.30 | 0.000000 | 0.000000 |
| *ACAD9* | 0.74 | 0.000003 | 0.000000 |  | 1.42 | 0.000000 | 0.000000 |
| *CDK2AP1* | 0.74 | 0.000000 | 0.000000 |  | 1.26 | 0.000000 | 0.000000 |
| *CST3* | 0.74 | 0.000000 | 0.000000 |  | 1.24 | 0.000000 | 0.000000 |
| *LIPE* | 0.74 | 0.000000 | 0.000000 |  | 1.30 | 0.000000 | 0.000000 |
| *PECR* | 0.74 | 0.000010 | 0.000000 |  | 1.34 | 0.000000 | 0.000000 |
| *ACSS2* | 0.73 | 0.000014 | 0.000000 |  | 1.41 | 0.000000 | 0.000000 |
| *ENO1* | 0.73 | 0.000000 | 0.000000 |  | 1.22 | 0.000000 | 0.000000 |
| *GAPDH* | 0.73 | 0.000000 | 0.000000 |  | 1.23 | 0.000000 | 0.000000 |
| *INHBB* | 0.73 | 0.000000 | 0.000000 |  | 1.31 | 0.000000 | 0.000000 |
| *ACADM* | 0.72 | 0.000926 | 0.000000 |  | 1.41 | 0.000000 | 0.000000 |
| *ATOX1* | 0.72 | 0.000001 | 0.000000 |  | 1.18 | 0.000000 | 0.000000 |
| *CYCS* | 0.71 | 0.000000 | 0.000000 |  | 1.30 | 0.000000 | 0.000000 |
| *DGAT1* | 0.71 | 0.000003 | 0.000000 |  | 1.51 | 0.000000 | 0.000000 |
| *GPT2* | 0.71 | 0.000000 | 0.000000 |  | 1.51 | 0.000000 | 0.000000 |
| *LDLR* | 0.71 | 0.000000 | 0.000000 |  | 1.37 | 0.000000 | 0.000000 |
| *PFKFB1* | 0.71 | 0.000003 | 0.000000 |  | 1.32 | 0.000000 | 0.000000 |
| *PDHA1* | 0.70 | 0.000004 | 0.000000 |  | 1.31 | 0.000000 | 0.000000 |
| *TMEM135* | 0.70 | 0.000000 | 0.000000 |  | 1.32 | 0.000000 | 0.000000 |
| *ALDOB* | 0.69 | 0.000002 | 0.000000 |  | 1.30 | 0.000005 | 0.000197 |
| *LDHA* | 0.69 | 0.000000 | 0.000000 |  | 1.26 | 0.000000 | 0.000000 |
| *SPTAN1* | 0.69 | 0.000541 | 0.000000 |  | 1.32 | 0.000000 | 0.000002 |
| *PGM1* | 0.68 | 0.000144 | 0.000000 |  | 1.33 | 0.000009 | 0.000000 |
| *CIDEC* | 0.67 | 0.000000 | 0.000000 |  | 1.55 | 0.000000 | 0.000000 |
| *GPT* | 0.66 | 0.000000 | 0.000000 |  | 1.56 | 0.000000 | 0.000000 |
| *TPST2* | 0.66 | 0.000000 | 0.000000 |  | 1.35 | 0.000000 | 0.000000 |
| *ACOX1* | 0.64 | 0.000000 | 0.000000 |  | 1.55 | 0.000000 | 0.000000 |
| *AQP1* | 0.64 | 0.000000 | 0.000000 |  | 1.74 | 0.000000 | 0.000000 |
| *VGLL3* | 0.64 | 0.000005 | 0.000000 |  | 1.33 | 0.000000 | 0.000000 |
| *CDKN2C* | 0.63 | 0.006735 | 0.000000 |  | 1.70 | 0.000000 | 0.000000 |
| *IDH1* | 0.63 | 0.000000 | 0.000000 |  | 1.46 | 0.000000 | 0.000000 |
| *GYS1* | 0.61 | 0.000000 | 0.000000 |  | 1.69 | 0.000000 | 0.000000 |
| *MECR* | 0.60 | 0.000000 | 0.000000 |  | 1.49 | 0.000000 | 0.000000 |
| *ELOVL5* | 0.59 | 0.000000 | 0.000000 |  | 1.67 | 0.000000 | 0.000000 |
| *ME1* | 0.59 | 0.000000 | 0.000000 |  | 1.60 | 0.000000 | 0.000000 |
| *THRSP* | 0.59 | 0.000000 | 0.000000 |  | 1.40 | 0.000000 | 0.000000 |
| *AACS* | 0.58 | 0.000000 | 0.000000 |  | 1.85 | 0.000000 | 0.000000 |
| *SREBF1* | 0.58 | 0.000000 | 0.000000 |  | 1.77 | 0.000000 | 0.000000 |
| *ACSL1* | 0.56 | 0.000000 | 0.000000 |  | 1.78 | 0.000000 | 0.000000 |
| *CCND1* | 0.56 | 0.000000 | 0.000000 |  | 1.43 | 0.000000 | 0.000000 |
| *LOXL2* | 0.56 | 0.000000 | 0.000000 |  | 1.50 | 0.000000 | 0.000000 |
| *LOX* | 0.55 | 0.000000 | 0.000000 |  | 1.50 | 0.000000 | 0.000000 |
| *PNPLA3* | 0.55 | 0.000002 | 0.000000 |  | 1.73 | 0.000000 | 0.000000 |
| *ECHDC1* | 0.54 | 0.000000 | 0.000000 |  | 1.70 | 0.000000 | 0.000000 |
| *LEP* | 0.49 | 0.000000 | 0.000000 |  | 1.54 | 0.000000 | 0.000000 |
| *KLB* | 0.48 | 0.000000 | 0.000000 |  | 1.91 | 0.000000 | 0.000000 |
| *DGAT2* | 0.44 | 0.000000 | 0.000000 |  | 2.18 | 0.000000 | 0.000000 |
| *FASN* | 0.44 | 0.000000 | 0.000000 |  | 2.40 | 0.000000 | 0.000000 |
| *CES1* | 0.43 | 0.000000 | 0.000000 |  | 1.53 | 0.000000 | 0.000000 |
| *THBS4* | 0.43 | 0.000000 | 0.000000 |  | 2.71 | 0.000000 | 0.000000 |
| *SFRP2* | 0.42 | 0.000000 | 0.000000 |  | 2.03 | 0.000000 | 0.000000 |
| *ALDOC* | 0.36 | 0.000000 | 0.000000 |  | 2.30 | 0.000000 | 0.000000 |
| *FADS1* | 0.32 | 0.000000 | 0.000000 |  | 2.77 | 0.000000 | 0.000000 |
| *FADS2* | 0.23 | 0.000000 | 0.000000 |  | 3.74 | 0.000000 | 0.000000 |
| *SCD* | 0.23 | 0.000000 | 0.000000 |  | 3.49 | 0.000000 | 0.000000 |
| *PRKAR2B* | 0.86 | 0.000239 | 0.000001 |  | 1.15 | 0.000677 | 0.000001 |
| *UQCRC2* | 0.83 | 0.008742 | 0.000000 |  | 1.22 | 0.000000 | 0.000000 |
| *PGAM1* | 0.77 | 0.000182 | 0.000000 |  | 1.20 | 0.000003 | 0.000000 |
