# Supplementary material for: Determinants of Human Adipose Tissue Gene Expression: Impact of Diet, Sex, Metabolic Status, and Cis Genetic Regulation
Source: PLoS Genet. 2012 Sep 27;8(9):e1002959. doi: 10.1371/journal.pgen.1002959 (PMC3459935; doi:10.1371/journal.pgen.1002959)
Supplement: Table S5 — Genes regulated at the end of the dietary intervention. A linear mixed effect model was ran separately for men and women with weight, clinical investigation day (CID) and diet as fixed, and centre and subject as random effect. The regressions equations tested without and with weight are displayed below:Y is the log2 expression value for gene i, in subject l, and centre k. The random term ε represents the random error that was assumed to be normally distributed. The Tukey HSD was used as post-hoc test. The Benjamini-Hochberg procedure was used to control for multiple testing. Only significant genes common to men and women and weight-independent genes are shown. *: Values refer to median mRNA level fold change from 311 subjects (107 men, 204 women). (DOCX) [file pgen.1002959.s010.docx]

**Table S5 Genes regulated at the end of the dietary intervention**

| **Gene Symbol** | **Fold Change*** | **P-value Men** | **P-value Women** |
| --- | --- | --- | --- |
| *ALOX12* | 1.47 | 0.020888 | 0.005253 |
| *VEGFA* | 1.28 | 0.000261 | 0.035924 |
| *AGPAT9* | 1.25 | 0.002702 | 0.024551 |
| *UCN* | 1.22 | 0.005981 | 0.005081 |
| *ANG* | 1.20 | 0.028077 | 0.008713 |
| *PCK2* | 1.17 | 0.014345 | 0.023043 |
| *AK2* | 1.16 | 0.001637 | 0.006844 |
| *NFKB2* | 1.14 | 0.002777 | 0.000550 |
| *TSEN54* | 1.14 | 0.000287 | 0.033677 |
| *IL4R* | 1.11 | 0.004426 | 0.000128 |
| *LPCAT1* | 1.09 | 0.000078 | 0.023043 |
| *HLA.A* | 1.08 | 0.000245 | 0.038048 |
| *CD97* | 1.07 | 0.029608 | 0.019681 |
| *E2F4* | 1.06 | 0.000261 | 0.045278 |
| *HK1* | 1.06 | 0.003006 | 0.026633 |
| *FABP4* | 0.96 | 0.032896 | 0.000499 |
| *LOXL2* | 0.86 | 0.047089 | 0.005253 |
| *ENO1* | 0.85 | 0.033411 | 0.000029 |
| *FTL* | 0.84 | 0.014644 | 0.000007 |
| *ITGB5* | 0.84 | 0.004679 | 0.000005 |
| *PGDS* | 0.84 | 0.016554 | 0.000677 |
| *C2* | 0.83 | 0.038526 | 0.043911 |
| *CSTB* | 0.83 | 0.014644 | 0.000007 |
| *CD68* | 0.82 | 0.000535 | 0.000514 |
| *MRC1L1* | 0.82 | 0.002858 | 0.000514 |
| *MS4A4A* | 0.82 | 0.030572 | 0.000517 |
| *CCL2* | 0.81 | 0.018450 | 0.028421 |
| *FBP1* | 0.81 | 0.000287 | 0.000029 |
| *MARCO* | 0.80 | 0.029608 | 0.009551 |
| *IL10* | 0.79 | 0.000243 | 0.010403 |
| *MMP19* | 0.79 | 0.000003 | 0.000007 |
| *C1QA* | 0.78 | 0.008627 | 0.000026 |
| *CD163* | 0.77 | 0.000749 | 0.000081 |
| *CD209* | 0.77 | 0.000535 | 0.000395 |
| *LIPA* | 0.77 | 0.001821 | 0.000000 |
| *C1QC* | 0.75 | 0.003042 | 0.000000 |
| *LEP* | 0.75 | 0.011476 | 0.000007 |
| *C1QB* | 0.74 | 0.009098 | 0.000005 |
| *AADACL1* | 0.73 | 0.000183 | 0.001213 |
| *MMP9* | 0.65 | 0.000788 | 0.002545 |
| *PLA2G7* | 0.62 | 0.000666 | 0.009551 |
| *FCGBP* | 0.52 | 0.000000 | 0.000000 |
| *CCL3* | 0.65 | 0.000000 | 0.001348 |
