# Supplementary material for: Determinants of Human Adipose Tissue Gene Expression: Impact of Diet, Sex, Metabolic Status, and Cis Genetic Regulation
Source: PLoS Genet. 2012 Sep 27;8(9):e1002959. doi: 10.1371/journal.pgen.1002959 (PMC3459935; doi:10.1371/journal.pgen.1002959)
Supplement: Table S6 — Markers of weight loss and weight regain during the weight maintenance diet phase. *: Values refer to ratio of mean mRNA level fold change from CID2 to CID3 between 31 women who gained (Regain) and 29 who lost (Loss) at least 50% of the weight lost during calorie restriction. £: Values refer to median mRNA level fold change between CID2 and CID3 in 31 women who gained at least 50% of the weight lost during calorie restriction. §: Values refer to median mRNA level fold change between CID2 and CID3 in 29 women who continued to lose at least 50% of the weight lost during calorie restriction. A linear mixed effect model was ran separately for men and women with weight, CID and diet as fixed, and centre and subject as random effect. The regression equation tested is displayed below:CID3/CID2Expression is the log2 expression value for gene i, in subject l, and centre k. The random term ε represents the random error that was assumed to be normally distributed. The Tukey HSD was used as post-hoc test. The Benjamini-Hochberg procedure was used to control for multiple testing. CID1, CID2 and CID3 are, respectively, clinical investigation days at baseline, after the 8-week calorie restriction and after the 26-week weight maintenance diet. (DOCX) [file pgen.1002959.s011.docx]

**Table S6. Markers of weight loss and weight regain during the weight maintenance diet phase**

| **Gene Symbol** | **Ratio Regain/Loss*** | **Regain Median^£^** | **Loss Median^§^** |
| --- | --- | --- | --- |
| *CIDEA* | 0.69 | 0.63 | 1.00 |
| *PCK2* | 0.83 | 0.86 | 1.13 |
| *LEP* | 0.95 | 1.54 | 1.17 |
| *CCND1* | 1.09 | 1.68 | 1.24 |
| *AP2M1* | 1.14 | 1.30 | 1.10 |
| *PKM2* | 1.16 | 1.15 | 1.02 |
| *ACTR3* | 1.18 | 1.11 | 1.00 |
| *INHBB* | 1.19 | 1.34 | 0.89 |
| *CSTB* | 1.27 | 1.15 | 0.93 |
| *LOXL2* | 1.29 | 1.76 | 1.17 |
| *CTSB* | 1.31 | 1.06 | 0.85 |
| *LOX* | 1.34 | 1.50 | 1.14 |
| *LDLR* | 1.37 | 1.53 | 0.99 |
| *CES1* | 1.37 | 2.02 | 1.00 |
| *FADS1* | 1.99 | 2.93 | 1.34 |
| *BCAT1* | 2.05 | 1.03 | 0.74 |
