# Supplementary material for: Determinants of Human Adipose Tissue Gene Expression: Impact of Diet, Sex, Metabolic Status, and Cis Genetic Regulation
Source: PLoS Genet. 2012 Sep 27;8(9):e1002959. doi: 10.1371/journal.pgen.1002959 (PMC3459935; doi:10.1371/journal.pgen.1002959)
Supplement: Table S7 — Sexual dimorphism of adipose tissue gene expression in 515 obese individuals. A linear mixed effect model was ran with gender as fixed and centre as random effect. The regression equation tested without and with fat mass (% of body weight) is displayed below:Y is the log2 expression value for gene i, in subject l, and centre k. The random term ε represents the random error that was assumed to be normally distributed. The Tukey HSD was used as post-hoc test. The Benjamini-Hochberg procedure was used to control for multiple testing. Only genes consistently significant among all phases of the dietary intervention were considered (see Figure S3). Bold indicates genes which sexual dimorphism is influenced by fat mass. *: Values refer to ratio of mean mRNA levels at baseline between 180 men and 335 women. (DOCX) [file pgen.1002959.s012.docx]

**Table S7.** **Sexual dimorphism of adipose tissue gene expression in 515 obese individuals**

| **Symbol** | **Ratio Men/Women*** | **Cytoband** | **Biological Function** |
| --- | --- | --- | --- |
| *PGM1* | 0.67 | 1p31 | carbohydrate metabolism |
| *CDKN2C* | 0.42 | 1p32 | cell cycle |
| ***C1QC*** | **0.68** | **1p36.11** | **immune response** |
| *C1QA* | 0.66 | 1p36.12 | immune response |
| ***C1QB*** | **0.68** | **1p36.12** | **immune response** |
| *MECR* | 0.64 | 1p36.1-p35.1 | lipid metabolism |
| *ENO1* | 0.78 | 1p36.3-p36.2 | carbohydrate metabolism |
| ***FCGR2B*** | **0.70** | **1q23** | **immune response** |
| *PRDX6* | 0.77 | 1q25.1 | response to stress |
| ***IL10*** | **0.82** | **1q31-q32** | **immune response** |
| *CST3* | 0.83 | 20p11.21 | protein metabolism |
| ***CTSZ*** | **0.86** | **20q13** | **protein metabolism** |
| *PCK1* | 0.59 | 20q13.31 | carbohydrate metabolism |
| *CSTB* | 0.78 | 21q22.3 | protein metabolism |
| *TPST2* | 0.72 | 22q12.1 | protein metabolism |
| ***INHBB*** | **0.82** | **2cen-q13** | **secreted factor** |
| *IDH1* | 0.71 | 2q33.3 | energy metabolism |
| *PECR* | 0.74 | 2q35 | lipid metabolism |
| *IRS1* | 0.58 | 2q36 | signal transduction |
| *SLC19A3* | 0.64 | 2q37 | transport |
| *ABHD5* | 0.75 | 3p21 | lipid metabolism |
| *GPD1L* | 0.76 | 3p22.3 | lipid metabolism |
| *OXSR1* | 0.82 | 3p22-p21.3 | response to stress |
| ***ITGB5*** | **0.82** | **3q21.2** | **immune response** |
| *ACAD9* | 0.75 | 3q21.3 | lipid metabolism |
| *RPN1* | 0.85 | 3q21.3 | lipid metabolism |
| *AP2M1* | 0.79 | 3q28 | signal transduction |
| *SLC4A4* | 0.54 | 4q21 | transport |
| *SFRP2* | 0.51 | 4q31.3 | cell differenciation |
| *ETFDH* | 0.77 | 4q32-q35 | energy metabolism |
| *ACSL1* | 0.70 | 4q34-q35 | lipid metabolism |
| *THBS4* | 0.57 | 5q13 | immune response |
| *COX7C* | 0.72 | 5q14 | energy metabolism |
| *ATOX1* | 0.76 | 5q32 | response to stress |
| ***CSF1R*** | **0.74** | **5q33-q35** | **immune response** |
| *VEGFA* | 0.75 | 6p12 | secreted factor |
| *ELOVL5* | 0.75 | 6p21.1-p12.1 | lipid metabolism |
| *AGPAT1* | 0.84 | 6p21.3 | lipid metabolism |
| ***ECHDC1*** | **0.74** | **6q22.33** | **lipid metabolism** |
| *CYCS* | 0.68 | 7p15.3 | energy metabolism |
| *RAC1* | 0.87 | 7p22 | immune response |
| *PRKAR2B* | 0.81 | 7q22 | signal transduction |
| *AZGP1* | 0.31 | 7q22.1 | secreted factor |
| *LEP* | 0.57 | 7q31.3 | secreted factor |
| *MEST* | 0.78 | 7q32 | cell differenciation |
| *EN2* | 0.61 | 7q36 | transcription |
| *LOXL2* | 0.83 | 8p21.3-p21.2 | cell adhesion |
| *ADHFE1* | 0.84 | 8q13.1 | ketone metabolism |
| *RDH10* | 0.57 | 8q21.11 | lipid metabolism |
| ***BAALC*** | **0.75** | **8q22.3** | **unknown** |
| *AQP7* | 0.75 | 9p13 | lipid metabolism |
| *CCL19* | 1.49 | 9p13 | immune response |
| *ALDOB* | 0.59 | 9q21.3-q22.2 | carbohydrate metabolism |
| *HSDL2* | 0.69 | 9q32 | lipid metabolism |
| ***MRC1L1*** | **0.74** | **10p12.33** | **immune response** |
| *PHYH* | 0.75 | 10p13 | lipid metabolism |
| *ECHDC3* | 0.68 | 10p14 | lipid metabolism |
| *SIRT1* | 0.84 | 10q21.3 | translation |
| *NDUFB8* | 0.80 | 10q23.2-q23.33 | energy metabolism |
| *SCD* | 0.53 | 10q24.31 | lipid metabolism |
| *PGAM1* | 0.80 | 10q25.3 | carbohydrate metabolism |
| *SAA4* | 0.31 | 11p15.1-p14 | immune response |
| *LDHA* | 0.70 | 11p15.4 | carbohydrate metabolism |
| *PNPLA2* | 0.76 | 11p15.5 | lipid metabolism |
| ***MS4A4A*** | **0.78** | **11q12** | **immune response** |
| ***MS4A6A*** | **0.81** | **11q12.1** | **immune response** |
| ***CCND1*** | **0.76** | **11q13** | **cell cycle** |
| *DGAT2* | 0.64 | 11q13.5 | lipid metabolism |
| *THRSP* | 0.63 | 11q13.5 | lipid metabolism |
| *TMEM135* | 0.77 | 11q14.2 | unknown |
| *ACAT1* | 0.66 | 11q22.3-q23.1 | lipid metabolism |
| ***GAPDH*** | **0.88** | **12p13** | **carbohydrate metabolism** |
| *TPI1* | 0.81 | 12p13 | carbohydrate metabolism |
| ***CD163*** | 0.78 | 12p13.3 | immune response |
| *CD163L1* | 0.65 | 12p13.3 | immune response |
| *NDUFA9* | 0.77 | 12p13.3 | energy metabolism |
| ***C3AR1*** | **0.83** | **12p13.31** | **immune response** |
| *IGF1* | 0.57 | 12q22-q23 | secreted factor |
| *PWP1* | 0.81 | 12q23.3 | transcription |
| *CDK2AP1* | 0.82 | 12q24.31 | cell cycle |
| *GPR109A* | 0.62 | 12q24.31 | lipid metabolism |
| *PCCA* | 0.71 | 13q32 | lipid metabolism |
| *NPAS3* | 0.85 | 14q12-q13 | transcription |
| *CKB* | 0.52 | 14q32 | energy metabolism |
| *EHD4* | 0.84 | 15q11.1 | signal transduction |
| *GATM* | 0.84 | 15q21.1 | energy metabolism |
| *ETFA* | 0.72 | 15q23-q25 | energy metabolism |
| *LOXL1* | 0.70 | 15q24-q25 | cell adhesion |
| *UQCRC2* | 0.72 | 16p12 | energy metabolism |
| *CARHSP1* | 0.81 | 16p13.2 | translation |
| *DCI* | 0.71 | 16p13.3 | lipid metabolism |
| *MT1E* | 0.77 | 16q13 | response to stress |
| *CES1* | 0.47 | 16q13-q22.1 | cell differenciation |
| *SLC2A4* | 0.53 | 17p13 | carbohydrate metabolism |
| ***CLEC10A*** | **0.70** | **17p13.1** | **immune response** |
| *ACOX1* | 0.74 | 17q24-q25 | lipid metabolism |
| *ATP5A1* | 0.73 | 18q12-q21 | energy metabolism |
| ***CD209*** | **0.88** | **19p13** | **immune response** |
| *DNASE2* | 0.81 | 19p13.2 | apoptosis |
| *AES* | 0.80 | 19p13.3 | transcription |
| ***POP4*** | **0.81** | **19q12** | **transport** |
| *LIPE* | 0.78 | 19q13.2 | lipid metabolism |
| *FCGRT* | 0.81 | 19q13.3 | immune response |
| *GYS1* | 0.66 | 19q13.3 | carbohydrate metabolism |
| *PFKFB1* | 0.70 | Xp11.21 | carbohydrate metabolism |
| *PDHA1* | 0.68 | Xp22.2-p22.1 | carbohydrate metabolism |
| *PGK1* | 0.79 | Xq13 | carbohydrate metabolism |
| ***SH3BGRL*** | **0.86** | **Xq13.3** | **unknown** |
| *TCEAL8* | 0.85 | Xq22.1 | transcription |
