# Supplementary material for: Determinants of Human Adipose Tissue Gene Expression: Impact of Diet, Sex, Metabolic Status, and Cis Genetic Regulation
Source: PLoS Genet. 2012 Sep 27;8(9):e1002959. doi: 10.1371/journal.pgen.1002959 (PMC3459935; doi:10.1371/journal.pgen.1002959)
Supplement: Table S8 — Differential adipose tissue gene expression according to presence and absence of metabolic syndrome in 515 obese individuals. P-value between non-metabolic syndrome and metabolic syndrome subjects as estimated using linear mixed effect model ran separately for men and women with metabolic syndrome status as fixed and centre as random effect, Tukey HSD as post-hoc test and Benjamini-Hochberg to control for multiple testing. The regression equation tested is displayed below:Y is the log2 expression value for gene i, in subject l, and centre k. The random term ε represents the random error that was assumed to be normally distributed. MS and non-MS are, respectively, subjects categorized according to the occurrence of metabolic syndrome [11], or not. The Tukey HSD was used as post-hoc test. *: Values refer to ratio of mean mRNA levels between metabolic syndrome (92 men, 114 women) and non-metabolic syndrome (88 men, 221 women) subjects at baseline (see Figure S3). (DOCX) [file pgen.1002959.s013.docx]

**Table S8. Differential adipose tissue gene expression according to presence and absence of metabolic syndrome in 515 obese individuals**

|  | **Men** | |  | **Women** | |
| --- | --- | --- | --- | --- | --- |
|  | **n = 180** | |  | **n = 335** | |
| **Genes** | **Ratio MS/non-MS*** | **P-value** |  | **Ratio MS/non-MS*** | **P-value** |
| *CCL3* | 1.38 | 0.01 |  | 1.61 | <0.005 |
| *ATF3* | 1.24 | 0.02 |  | 1.35 | <0.005 |
| *HSDL2* | 0.88 | 0.04 |  | 0.72 | <0.005 |
| *ETFDH* | 0.86 | 0.02 |  | 0.83 | <0.005 |
| *ADHFE1* | 0.86 | 0.04 |  | 0.83 | <0.005 |
| *CIDEC* | 0.85 | 0.04 |  | 0.78 | <0.005 |
| *GPD1L* | 0.84 | 0.01 |  | 0.79 | <0.005 |
| *HADH* | 0.84 | 0.02 |  | 0.83 | <0.005 |
| *FGF2* | 0.83 | 0.02 |  | 0.79 | <0.005 |
| *ECHDC3* | 0.80 | 0.02 |  | 0.72 | <0.005 |
| *ME1* | 0.79 | 0.02 |  | 0.67 | <0.005 |
| *ELOVL5* | 0.78 | <0.005 |  | 0.81 | <0.005 |
| *CKB* | 0.77 | 0.02 |  | 0.71 | <0.005 |
| *SLC2A4* | 0.77 | 0.02 |  | 0.63 | <0.005 |
| *GYS1* | 0.75 | <0.005 |  | 0.80 | <0.005 |
| *GPT* | 0.75 | 0.01 |  | 0.76 | <0.005 |
| *GPT2* | 0.75 | 0.04 |  | 0.76 | <0.005 |
| *ACSL1* | 0.74 | <0.005 |  | 0.79 | <0.005 |
| *FASN* | 0.73 | 0.02 |  | 0.52 | <0.005 |
| *CDKN2C* | 0.71 | 0.02 |  | 0.71 | <0.005 |
| *FADS2* | 0.70 | 0.04 |  | 0.57 | <0.005 |
| *AZGP1* | 0.63 | <0.005 |  | 0.58 | <0.005 |
