# Supplementary material for: Determinants of Human Adipose Tissue Gene Expression: Impact of Diet, Sex, Metabolic Status, and Cis Genetic Regulation
Source: PLoS Genet. 2012 Sep 27;8(9):e1002959. doi: 10.1371/journal.pgen.1002959 (PMC3459935; doi:10.1371/journal.pgen.1002959)
Supplement: Table S9 — Obesity-related genes in women along the dietary intervention. Values refer to ratio of mean mRNA level from highest and lowest BMI decile subjects in 204 women from group A. CID1, CID2 and CID3 are, respectively, clinical investigation days at baseline, after the 8-week calorie restriction and after the 26-week weight maintenance diet. A linear mixed effect model was run with BMI as fixed and centre as random effect at each time point. Diet was entered as supplementary fixed effect in the model investigating genes during weight maintenance diet. The regressions equations tested without and with change in BMI are displayed below: Y is the log2 expression value for gene i, in subject l, centre k and Diet d. The random term ε represents the random error that was assumed to be normally distributed. The Benjamini-Hochberg procedure was used to control for multiple testing. (DOCX) [file pgen.1002959.s014.docx]

**Table S9. Obesity-related genes in women along the dietary intervention**

|  | CID1 | | CID2 | | CID3 | |
| --- | --- | --- | --- | --- | --- | --- |
| n high/low BMI | 22/18 |  | 23/18 |  | 21/21 |  |
| Gene Symbol |  |  |  |  |  |  |
| SPP1 | 3.05 |  | 1.24 |  | 2.24 |  |
| CCL19 | 2.25 |  | 3.07 |  | 1.91 |  |
| PLA2G7 | 2.23 |  | 1.73 |  | 1.46 |  |
| FCGBP | 2.04 |  | 1.87 |  | 1.78 |  |
| HMOX1 | 1.96 |  | 1.95 |  | 2.06 |  |
| ATF3 | 1.95 |  | 1.36 |  | 1.78 |  |
| IL1RN | 1.92 |  | 1.47 |  | 2.41 |  |
| IL10 | 1.86 |  | 1.87 |  | 1.84 |  |
| MARCO | 1.86 |  | 1.59 |  | 1.59 |  |
| C1QA | 1.83 |  | 1.76 |  | 1.55 |  |
| AADACL1 | 1.80 |  | 1.25 |  | 1.68 |  |
| MS4A7 | 1.76 |  | 1.69 |  | 1.20 |  |
| CCL3 | 1.65 |  | 1.76 |  | 2.41 |  |
| C1QB | 1.65 |  | 1.87 |  | 1.25 |  |
| PGDS | 1.64 |  | 1.90 |  | 1.73 |  |
| CD163 | 1.64 |  | 2.01 |  | 1.36 |  |
| FN1 | 1.62 |  | 1.50 |  | 1.63 |  |
| MRC1L1 | 1.60 |  | 1.56 |  | 1.55 |  |
| C1QC | 1.51 |  | 1.54 |  | 1.21 |  |
| MS4A6A | 1.46 |  | 1.78 |  | 1.36 |  |
| LIPA | 1.46 |  | 1.36 |  | 1.40 |  |
| CD68 | 1.44 |  | 1.36 |  | 1.48 |  |
| IFI30 | 1.38 |  | 1.20 |  | 1.56 |  |
| MMP19 | 1.37 |  | 1.38 |  | 1.42 |  |
| C2 | 1.34 |  | 1.49 |  | 1.28 |  |
| CD9 | 1.28 |  | 1.35 |  | 1.51 |  |
| CCL2 | 1.27 |  | 1.95 |  | 2.05 |  |
| ROBO3 | 0.83 |  | 0.67 |  | 0.77 |  |
| PCK2 | 0.81 |  | 0.61 |  | 0.60 |  |
| SLC19A2 | 0.75 |  | 0.61 |  | 0.69 |  |
| ACACB | 0.75 |  | 0.72 |  | 0.61 |  |
| CTH | 0.72 |  | 0.56 |  | 0.62 |  |
| FUZ | 0.71 |  | 0.72 |  | 0.76 |  |
| TWIST1 | 0.69 |  | 0.64 |  | 0.67 |  |
| ALDH6A1 | 0.67 |  | 0.62 |  | 0.49 |  |
| HADH | 0.67 |  | 0.71 |  | 0.76 |  |
| ABHD5 | 0.66 |  | 0.73 |  | 0.61 |  |
| DCI | 0.65 |  | 0.68 |  | 0.72 |  |
| VEGFA | 0.62 |  | 0.67 |  | 0.75 |  |
| GPD1L | 0.62 |  | 0.56 |  | 0.57 |  |
| ECHDC3 | 0.62 |  | 0.77 |  | 0.78 |  |
| CKB | 0.56 |  | 0.58 |  | 0.50 |  |
| ADHFE1 | 0.56 |  | 0.68 |  | 0.69 |  |
| GPT | 0.56 |  | 0.65 |  | 0.61 |  |
| IRS2 | 0.50 |  | 0.52 |  | 0.57 |  |
| LPIN1 | 0.49 |  | 0.62 |  | 0.62 |  |
| CIDEA | 0.48 |  | 0.48 |  | 0.35 |  |
| AGPAT9 | 0.47 |  | 0.50 |  | 0.50 |  |
| SLC2A4 | 0.40 |  | 0.47 |  | 0.51 |  |
| AZGP1 | 0.37 |  | 0.36 |  | 0.34 |  |
| FASN | 0.33 |  | 0.42 |  | 0.42 |  |
