# Supplementary material for: Determinants of Human Adipose Tissue Gene Expression: Impact of Diet, Sex, Metabolic Status, and Cis Genetic Regulation
Source: PLoS Genet. 2012 Sep 27;8(9):e1002959. doi: 10.1371/journal.pgen.1002959 (PMC3459935; doi:10.1371/journal.pgen.1002959)
Supplement: Table S10 — Genes selected for network analysis at baseline. (DOCX) [file pgen.1002959.s015.docx]

**Table S10** Genes selected for network analysis at baseline.

| **Gene symbol** | **Gene name** |
| --- | --- |
| AADACL1 | neutral cholesterol ester hydrolase 1 |
| ACSL1 | acyl-CoA synthetase long-chain family member 1 |
| AGPAT9 | 1-acylglycerol-3-phosphate O-acyltransferase 9 |
| ALDOB | aldolase B, fructose-bisphosphate |
| ALDOC | aldolase C, fructose-bisphosphate |
| AZGP1 | alpha-2-glycoprotein 1, zinc-binding |
| BCAT1 | branched chain amino-acid transaminase 1, cytosolic |
| CCL3 | chemokine (C-C motif) ligand 3 |
| CCND1 | cyclin D1 |
| CD209 | CD209 molecule |
| CD68 | CD68 molecule |
| CES1 | carboxylesterase 1 |
| CIDEA | cell death-inducing DFFA-like effector a |
| ECHDC3 | enoyl Coenzyme A hydratase domain containing 3 |
| ECHDC1 | enoyl CoA hydratase domain containing 1 |
| ELOVL5 | ELOVL fatty acid elongase 5 |
| FADS1 | fatty acid desaturase 1 |
| FADS2 | fatty acid desaturase 2 |
| FASN | fatty acid synthase |
| FBP1 | fructose-1,6-bisphosphatase 1 |
| FCGBP | Fc fragment of IgG binding protein |
| GYS1 | glycogen synthase 1 (muscle) |
| HLA.A | major histocompatibility complex, class I, A |
| HSDL2 | hydroxysteroid dehydrogenase like 2 |
| IL10 | interleukin 10 |
| INHBB | inhibin, beta B |
| LEP | leptin |
| LIPA | lipase A, lysosomal acid, cholesterol esterase |
| LOX | lysyl oxidase |
| LOXL2 | lysyl oxidase-like 2 |
| LPCAT1 | lysophosphatidylcholine acyltransferase 1 |
| MARCO | macrophage receptor with collagenous structure |
| ME1 | malic enzyme 1, NADP(+)-dependent, cytosolic |
| MMP19 | matrix metallopeptidase 19 |
| MMP9 | matrix metallopeptidase 9 (gelatinase B, 92kDa gelatinase, 92kDa type IV collagenase) |
| PLA2G7 | phospholipase A2, group VII (platelet-activating factor acetylhydrolase, plasma) |
| SCD | stearoyl-CoA desaturase (delta-9-desaturase) |
| SPP1 | secreted phosphoprotein 1 |
