# Supplementary material for: Determinants of Human Adipose Tissue Gene Expression: Impact of Diet, Sex, Metabolic Status, and Cis Genetic Regulation
Source: PLoS Genet. 2012 Sep 27;8(9):e1002959. doi: 10.1371/journal.pgen.1002959 (PMC3459935; doi:10.1371/journal.pgen.1002959)
Supplement: Table S11 — Lipogenic and glycolytic genes selected for network analysis during dietary intervention. Genes were selected as part of the lipogenic module observed in Figure S5 with other genes encoding proteins involved in lipogenesis or glucose metabolism. (DOCX) [file pgen.1002959.s016.docx]

**Table S11** Lipogenic and glycolytic genes selected for network analysis during dietary intervention.

| **Gene symbol** | **Gene name** |
| --- | --- |
| ACACB | acetyl-CoA carboxylase beta |
| ALDOB | aldolase B, fructose-bisphosphate |
| ALDOC | aldolase C, fructose-bisphosphate |
| ELOVL5 | ELOVL fatty acid elongase 5 |
| ENO1 | enolase 1, (alpha) |
| ENO3 | enolase 3 (beta, muscle) |
| FADS1 | fatty acid desaturase 1 |
| FADS2 | fatty acid desaturase 2 |
| FASN | fatty acid synthase |
| GAPDH | glyceraldehyde-3-phosphate dehydrogenase |
| HK1 | hexokinase 1 |
| LDHA | lactate dehydrogenase A |
| ME1 | malic enzyme 1, NADP(+)-dependent, cytosolic |
| PC | pyruvate carboxylase |
| PCK1 | phosphoenolpyruvate carboxykinase 1 (soluble) |
| PFKM | phosphofructokinase, muscle |
| PGAM1 | phosphoglycerate mutase 1 (brain) |
| PGK1 | phosphoglycerate kinase 1 |
| PGM1 | phosphoglucomutase 1 |
| PKM2 | pyruvate kinase, muscle |
| PDHA1 | pyruvate dehydrogenase (lipoamide) alpha 1 |
| SCD | stearoyl-CoA desaturase (delta-9-desaturase) |
| SLC2A4 | solute carrier family 2 (facilitated glucose transporter), member 4 |
