# Supplementary material for: Determinants of Human Adipose Tissue Gene Expression: Impact of Diet, Sex, Metabolic Status, and Cis Genetic Regulation
Source: PLoS Genet. 2012 Sep 27;8(9):e1002959. doi: 10.1371/journal.pgen.1002959 (PMC3459935; doi:10.1371/journal.pgen.1002959)
Supplement: Table S12 — SNPs showing associations with adipose tissue gene expression at baseline. Gene expression data were log transformed. Association of 2953 SNPs with gene expression was tested at baseline in 422 individuals (148 men and 274 women) using a linear mixed model with gender (and BMI) as fixed and centre (and diet) as random effect assuming a linear log-additive allele-dose effect with each SNP coded 0, 1 and 2 according to the number of minor alleles an individual carries (0 = wild-type, 1 = heterozygote, 2 = homozygote). The regression equation displayed below was tested without then with BMI:Y is the log2 expression value for gene i, in subject l, and centre k. The random term ε represents the random error that was assumed to be normally distributed. Gender difference between genotype distribution was investigated using a chi2 test to test the independence between the people belonging to the individual and the value criteria. For regression expected values >5, the non-parametric Fisher's exact test was used. The Benjamini-Hochberg procedure was used to control for multiple testing. CID1 is clinical investigation day at baseline. (DOCX) [file pgen.1002959.s017.docx]

**Table S12. SNPs showing associations with adipose tissue gene expression at baseline**

| **Gene** | **SNP** | **Total SNPs #** | **M/m alleles** | **MAF(%)** | **Homozygous Wild-type** | **Heterozygote** | **Homozygous Mutant** | **P-value** |
| --- | --- | --- | --- | --- | --- | --- | --- | --- |
| *ACSL1* | rs3792311 | 13 | G/A | 37.3 | 168 ( 39.8 ; G/G ) | 193 ( 45.7 ; G/A ) | 61 ( 14.5 ; A/A ) | 0.04111 |
| *ADH1C* | rs1614377 | 10 | G/A | 28.7 | 214 ( 50.8 ; G/G ) | 172 ( 40.9 ; G/A ) | 35 ( 8.3 ; A/A ) | 0.00228 |
|  | rs1614972 | 10 | C/T | 30.5 | 204 ( 48.5 ; C/C ) | 177 ( 42 ; C/T ) | 40 ( 9.5 ; T/T ) | 0.00003 |
|  | rs1662037 | 10 | G/A | 28.9 | 214 ( 50.7 ; G/G ) | 172 ( 40.8 ; G/A ) | 36 ( 8.5 ; A/A ) | 0.00225 |
|  | rs1789924 | 10 | C/T | 38.7 | 157 ( 37.2 ; C/C ) | 203 ( 48.1 ; C/T ) | 62 ( 14.7 ; T/T ) | 0.00003 |
|  | rs2851300 | 10 | C/T | 38.7 | 157 ( 37.2 ; C/C ) | 203 ( 48.1 ; C/T ) | 62 ( 14.7 ; T/T ) | 0.00003 |
|  | rs698 | 10 | T/C | 38.8 | 155 ( 36.9 ; T/T ) | 204 ( 48.6 ; T/C ) | 61 ( 14.5 ; C/C ) | 0.00004 |
|  | rs9307239 | 10 | C/T | 39.2 | 158 ( 37.4 ; C/C ) | 197 ( 46.7 ; C/T ) | 67 ( 15.9 ; T/T ) | 0.01454 |
| *ALDOB* | rs479600 | 7 | C/T | 24.1 | 249 ( 59 ; C/C ) | 143 ( 33.9 ; T/C ) | 30 ( 7.1 ; T/T ) | 0.00000 |
|  | rs533017 | 7 | T/C | 33.1 | 198 ( 46.9 ; T/T ) | 169 ( 40 ; T/C ) | 55 ( 13 ; C/C ) | 0.00191 |
|  | rs550915 | 7 | A/C | 14.7 | 306 ( 73.2 ; A/A ) | 101 ( 24.2 ; A/C ) | 11 ( 2.6 ; C/C ) | 0.00908 |
| *ANG* | rs1010458 | 10 | G/A | 18.2 | 282 ( 66.8 ; G/G ) | 126 ( 29.9 ; G/A ) | 14 ( 3.3 ; A/A ) | 0.04123 |
| *AP2M1* | rs843346 | 8 | C/T | 20.6 | 259 ( 61.4 ; C/C ) | 152 ( 36 ; C/T ) | 11 ( 2.6 ; T/T ) | 0.04860 |
| *ATP8A1* | rs4550967 | 45 | A/C | 32.9 | 194 ( 46.1 ; A/A ) | 177 ( 42 ; A/C ) | 50 ( 11.9 ; C/C ) | 0.02306 |
|  | rs6851867 | 45 | G/A | 29.1 | 206 ( 48.8 ; G/G ) | 186 ( 44.1 ; G/A ) | 30 ( 7.1 ; A/A ) | 0.01203 |
| *CARHSP1* | rs10163323 | 7 | G/A | 12.7 | 311 ( 77 ; G/G ) | 83 ( 20.5 ; G/A ) | 10 ( 2.5 ; A/A ) | 0.01832 |
|  | rs4985053 | 7 | T/C | 25.2 | 234 ( 55.5 ; T/T ) | 163 ( 38.6 ; C/T ) | 25 ( 5.9 ; C/C ) | 0.01755 |
| *CCL19* | rs10972195 | 3 | C/A | 22.3 | 254 ( 60.2 ; C/C ) | 148 ( 35.1 ; C/A ) | 20 ( 4.7 ; A/A ) | 0.01354 |
| *CCL3* | rs1634508 | 7 | C/A | 26.7 | 228 ( 54 ; C/C ) | 163 ( 38.6 ; C/A ) | 31 ( 7.3 ; A/A ) | 0.00025 |
|  | rs1719220 | 7 | C/T | 30.5 | 205 ( 48.6 ; C/C ) | 177 ( 41.9 ; C/T ) | 40 ( 9.5 ; T/T ) | 0.01854 |
|  | rs1851503 | 7 | A/C | 35 | 180 ( 42.7 ; A/A ) | 189 ( 44.8 ; A/C ) | 53 ( 12.6 ; C/C ) | 0.00589 |
|  | rs9972960 | 7 | G/A | 36.8 | 172 ( 41 ; G/G ) | 187 ( 44.5 ; G/A ) | 61 ( 14.5 ; A/A ) | 0.00316 |
| *CD14* | rs12517200 | 7 | A/G | 31.9 | 201 ( 47.6 ; A/A ) | 173 ( 41 ; A/G ) | 48 ( 11.4 ; G/G ) | 0.03020 |
|  | rs2569193 | 7 | G/A | 31.4 | 205 ( 48.6 ; G/G ) | 169 ( 40 ; G/A ) | 48 ( 11.4 ; A/A ) | 0.02185 |
|  | rs3822356 | 7 | A/G | 23.9 | 241 ( 57.1 ; A/A ) | 160 ( 37.9 ; A/G ) | 21 ( 5 ; G/G ) | 0.02037 |
|  | rs7721577 | 7 | T/C | 23.8 | 243 ( 57.6 ; T/T ) | 157 ( 37.2 ; T/C ) | 22 ( 5.2 ; C/C ) | 0.01573 |
| *CD48* | rs1126644 | 10 | T/C | 22.9 | 248 ( 58.8 ; T/T ) | 155 ( 36.7 ; C/T ) | 19 ( 4.5 ; C/C ) | 0.02075 |
| *CDK2AP1* | rs10846489 | 1 | A/G | 31.3 | 202 ( 47.9 ; A/A ) | 176 ( 41.7 ; G/A ) | 44 ( 10.4 ; G/G ) | 0.00918 |
| *CIDEA* | rs10853223 | 12 | T/C | 45.6 | 129 ( 30.6 ; T/T ) | 201 ( 47.6 ; T/C ) | 92 ( 21.8 ; C/C ) | 0.03874 |
|  | rs12966216 | 12 | C/T | 37.4 | 159 ( 39.2 ; C/C ) | 190 ( 46.8 ; C/T ) | 57 ( 14 ; T/T ) | 0.00000 |
|  | rs7230480 | 12 | C/T | 23.8 | 246 ( 58.3 ; C/C ) | 151 ( 35.8 ; T/C ) | 25 ( 5.9 ; T/T ) | 0.02857 |
|  | rs7504200 | 12 | A/C | 48.6 | 106 ( 25.1 ; A/A ) | 222 ( 52.6 ; C/A ) | 94 ( 22.3 ; C/C ) | 0.00003 |
| *CSTB* | rs2838363 | 7 | T/C | 48 | 116 ( 27.8 ; T/T ) | 202 ( 48.4 ; C/T ) | 99 ( 23.7 ; C/C ) | 0.02037 |
|  | rs3761385 | 7 | T/G | 30.7 | 203 ( 48.1 ; T/T ) | 179 ( 42.4 ; T/G ) | 40 ( 9.5 ; G/G ) | 0.00575 |
| *CTSZ* | rs163801 | 5 | G/A | 19.8 | 276 ( 65.6 ; G/G ) | 123 ( 29.2 ; A/G ) | 22 ( 5.2 ; A/A ) | 0.02489 |
| *CYYR1* | rs1395053 | 43 | C/T | 46.7 | 127 ( 30.1 ; C/C ) | 196 ( 46.4 ; T/C ) | 99 ( 23.5 ; T/T ) | 0.00521 |
|  | rs219672 | 43 | A/G | 36.5 | 169 ( 40 ; A/A ) | 198 ( 46.9 ; A/G ) | 55 ( 13 ; G/G ) | 0.02037 |
|  | rs222956 | 43 | T/C | 23.9 | 250 ( 59.2 ; T/T ) | 142 ( 33.6 ; C/T ) | 30 ( 7.1 ; C/C ) | 0.01111 |
| *ECHDC3* | rs2055040 | 6 | C/T | 34.6 | 181 ( 42.9 ; C/C ) | 190 ( 45 ; T/C ) | 51 ( 12.1 ; T/T ) | 0.00689 |
| *EHD4* | rs10518743 | 26 | C/T | 11.7 | 325 ( 77 ; C/C ) | 95 ( 22.5 ; C/T ) | 2 ( 0.5 ; T/T ) | 0.00000 |
|  | rs16972308 | 26 | G/A | 11.8 | 322 ( 76.8 ; G/G ) | 95 ( 22.7 ; G/A ) | 2 ( 0.5 ; A/A ) | 0.00000 |
|  | rs17739167 | 26 | A/G | 29.4 | 210 ( 49.8 ; A/A ) | 176 ( 41.7 ; A/G ) | 36 ( 8.5 ; G/G ) | 0.03261 |
| *EN2* | rs2885339 | 4 | T/G | 14.5 | 301 ( 72.4 ; T/T ) | 109 ( 26.2 ; G/T ) | 6 ( 1.4 ; G/G ) | 0.02791 |
| *FADS1* | rs174546 | 4 | C/T | 31.9 | 202 ( 47.9 ; C/C ) | 171 ( 40.5 ; C/T ) | 49 ( 11.6 ; T/T ) | 0.04056 |
|  | rs174556 | 4 | C/T | 26.7 | 231 ( 54.7 ; C/C ) | 157 ( 37.2 ; C/T ) | 34 ( 8.1 ; T/T ) | 0.01176 |
| *FBP1* | rs4129219 | 12 | T/C | 16.1 | 298 ( 70.6 ; T/T ) | 112 ( 26.5 ; T/C ) | 12 ( 2.8 ; C/C ) | 0.03128 |
| *FCER1G* | rs2070902 | 9 | C/T | 24.3 | 237 ( 56.2 ; C/C ) | 165 ( 39.1 ; C/T ) | 20 ( 4.7 ; T/T ) | 0.01582 |
|  | rs4233368 | 9 | C/A | 23.8 | 239 ( 56.6 ; C/C ) | 165 ( 39.1 ; C/A ) | 18 ( 4.3 ; A/A ) | 0.02037 |
|  | rs4489574 | 9 | C/T | 30.3 | 198 ( 46.9 ; C/C ) | 192 ( 45.5 ; C/T ) | 32 ( 7.6 ; T/T ) | 0.01011 |
| *GATM* | rs1346268 | 3 | T/C | 28.7 | 222 ( 52.6 ; T/T ) | 158 ( 37.4 ; T/C ) | 42 ( 10 ; C/C ) | 0.00000 |
|  | rs2461700 | 3 | T/C | 39.8 | 159 ( 37.7 ; T/T ) | 190 ( 45 ; T/C ) | 73 ( 17.3 ; C/C ) | 0.00025 |
|  | rs4774580 | 3 | G/A | 28.4 | 224 ( 53.1 ; G/G ) | 156 ( 37 ; G/A ) | 42 ( 10 ; A/A ) | 0.00000 |
| *HLA-A* | rs16896742 | 4 | A/G | 35.9 | 178 ( 42.8 ; A/A ) | 177 ( 42.5 ; A/G ) | 61 ( 14.7 ; G/G ) | 0.00000 |
|  | rs2860580 | 4 | G/A | 44 | 138 ( 32.7 ; G/G ) | 197 ( 46.7 ; A/G ) | 87 ( 20.6 ; A/A ) | 0.00000 |
| *HP* | rs1424241 | 2 | G/A | 18.1 | 281 ( 66.6 ; G/G ) | 129 ( 30.6 ; G/A ) | 12 ( 2.8 ; A/A ) | 0.00017 |
| *HSDL2* | rs10817330 | 12 | G/A | 40.5 | 153 ( 36.3 ; G/G ) | 196 ( 46.4 ; A/G ) | 73 ( 17.3 ; A/A ) | 0.00075 |
|  | rs10817344 | 12 | G/T | 34.7 | 183 ( 43.4 ; G/G ) | 185 ( 43.8 ; T/G ) | 54 ( 12.8 ; T/T ) | 0.00191 |
|  | rs16916995 | 12 | G/A | 29.1 | 211 ( 50 ; G/G ) | 176 ( 41.7 ; G/A ) | 35 ( 8.3 ; A/A ) | 0.00414 |
|  | rs4978489 | 12 | T/C | 31.5 | 198 ( 46.9 ; T/T ) | 182 ( 43.1 ; T/C ) | 42 ( 10 ; C/C ) | 0.00226 |
|  | rs4979108 | 12 | C/T | 34.2 | 186 ( 44.1 ; C/C ) | 183 ( 43.4 ; C/T ) | 53 ( 12.6 ; T/T ) | 0.00253 |
| *IL1RN* | rs315948 | 8 | C/T | 15.6 | 300 ( 71.1 ; C/C ) | 112 ( 26.5 ; C/T ) | 10 ( 2.4 ; T/T ) | 0.04454 |
|  | rs315952 | 8 | T/C | 29.3 | 209 ( 49.6 ; T/T ) | 177 ( 42 ; T/C ) | 35 ( 8.3 ; C/C ) | 0.04894 |
| *IRF5* | rs4728142 | 4 | G/A | 45.3 | 130 ( 30.8 ; G/G ) | 202 ( 47.9 ; G/A ) | 90 ( 21.3 ; A/A ) | 0.02090 |
| *ITGB5* | rs1007856 | 34 | A/G | 42.7 | 143 ( 33.9 ; A/A ) | 198 ( 46.9 ; A/G ) | 81 ( 19.2 ; G/G ) | 0.00314 |
|  | rs11928651 | 34 | T/C | 42.7 | 144 ( 34.1 ; T/T ) | 196 ( 46.4 ; T/C ) | 82 ( 19.4 ; C/C ) | 0.00433 |
|  | rs16836080 | 34 | G/A | 26.4 | 231 ( 55.3 ; G/G ) | 153 ( 36.6 ; G/A ) | 34 ( 8.1 ; A/A ) | 0.02268 |
|  | rs4141663 | 34 | C/T | 43.5 | 138 ( 32.7 ; C/C ) | 201 ( 47.6 ; C/T ) | 83 ( 19.7 ; T/T ) | 0.00374 |
|  | rs9968182 | 34 | C/T | 42.4 | 145 ( 34.4 ; C/C ) | 196 ( 46.4 ; C/T ) | 81 ( 19.2 ; T/T ) | 0.00314 |
| *KLB* | rs2381378 | 19 | G/T | 17.5 | 290 ( 68.9 ; G/G ) | 115 ( 27.3 ; G/T ) | 16 ( 3.8 ; T/T ) | 0.01111 |
|  | rs900563 | 19 | C/A | 17.6 | 288 ( 68.6 ; C/C ) | 116 ( 27.6 ; C/A ) | 16 ( 3.8 ; A/A ) | 0.00930 |
| *LILRA6* | rs4575638 | 6 | A/G | 31.1 | 191 ( 45.4 ; A/A ) | 198 ( 47 ; A/G ) | 32 ( 7.6 ; G/G ) | 0.00146 |
| *LILRB3* | rs13353326 | 4 | G/A | 11.8 | 330 ( 78.2 ; G/G ) | 84 ( 19.9 ; G/A ) | 8 ( 1.9 ; A/A ) | 0.00000 |
|  | rs3865475 | 4 | T/C | 39.2 | 163 ( 38.6 ; T/T ) | 187 ( 44.3 ; T/C ) | 72 ( 17.1 ; C/C ) | 0.00010 |
| *LIPA* | rs1051338 | 33 | T/G | 26.4 | 228 ( 54.2 ; T/T ) | 164 ( 39 ; T/G ) | 29 ( 6.9 ; G/G ) | 0.04606 |
|  | rs2243547 | 33 | A/C | 27.4 | 221 ( 52.5 ; A/A ) | 169 ( 40.1 ; A/C ) | 31 ( 7.4 ; C/C ) | 0.04447 |
|  | rs2243548 | 33 | C/T | 49.2 | 102 ( 24.4 ; C/C ) | 221 ( 52.9 ; C/T ) | 95 ( 22.7 ; T/T ) | 0.02278 |
|  | rs2250645 | 33 | C/T | 25.5 | 236 ( 55.9 ; C/C ) | 157 ( 37.2 ; C/T ) | 29 ( 6.9 ; T/T ) | 0.03565 |
| *LOX* | rs2731647 | 6 | A/G | 49.8 | 110 ( 26.1 ; A/A ) | 204 ( 48.3 ; A/G ) | 108 ( 25.6 ; G/G ) | 0.01176 |
|  | rs3853401 | 6 | G/A | 29.5 | 214 ( 50.7 ; G/G ) | 167 ( 39.6 ; G/A ) | 41 ( 9.7 ; A/A ) | 0.01249 |
| *LY86* | rs1999652 | 40 | G/T | 28.3 | 209 ( 50.9 ; G/G ) | 171 ( 41.6 ; T/G ) | 31 ( 7.5 ; T/T ) | 0.00066 |
|  | rs3804474 | 40 | T/C | 47.6 | 125 ( 29.6 ; T/T ) | 192 ( 45.5 ; C/T ) | 105 ( 24.9 ; C/C ) | 0.00172 |
|  | rs6597220 | 40 | T/C | 23.9 | 239 ( 56.6 ; T/T ) | 164 ( 38.9 ; C/T ) | 19 ( 4.5 ; C/C ) | 0.00000 |
|  | rs9328374 | 40 | A/G | 20 | 267 ( 63.3 ; A/A ) | 141 ( 33.4 ; G/A ) | 14 ( 3.3 ; G/G ) | 0.00021 |
|  | rs9328376 | 40 | C/T | 39.3 | 153 ( 36.3 ; C/C ) | 206 ( 48.8 ; C/T ) | 63 ( 14.9 ; T/T ) | 0.02278 |
|  | rs9405942 | 40 | G/A | 23.7 | 241 ( 57.1 ; G/G ) | 162 ( 38.4 ; A/G ) | 19 ( 4.5 ; A/A ) | 0.00000 |
|  | rs977785 | 40 | C/A | 24.2 | 237 ( 56.2 ; C/C ) | 166 ( 39.3 ; A/C ) | 19 ( 4.5 ; A/A ) | 0.00000 |
|  | rs1905045 | 9 | T/C | 24.4 | 244 ( 57.8 ; T/T ) | 150 ( 35.5 ; C/T ) | 28 ( 6.6 ; C/C ) | 0.01542 |
| *MARCO* | rs11693199 | 17 | T/C | 35.9 | 170 ( 40.3 ; T/T ) | 201 ( 47.6 ; T/C ) | 51 ( 12.1 ; C/C ) | 0.00032 |
|  | rs12997897 | 17 | G/A | 35.7 | 174 ( 41.2 ; G/G ) | 195 ( 46.2 ; G/A ) | 53 ( 12.6 ; A/A ) | 0.00059 |
|  | rs1371562 | 17 | G/T | 38.7 | 156 ( 37 ; G/G ) | 205 ( 48.6 ; G/T ) | 61 ( 14.5 ; T/T ) | 0.00255 |
|  | rs17009716 | 17 | C/T | 12 | 328 ( 77.7 ; C/C ) | 87 ( 20.6 ; C/T ) | 7 ( 1.7 ; T/T ) | 0.02026 |
|  | rs17796260 | 17 | G/A | 14.5 | 307 ( 72.7 ; G/G ) | 108 ( 25.6 ; G/A ) | 7 ( 1.7 ; A/A ) | 0.00000 |
|  | rs2011839 | 17 | C/T | 37.9 | 162 ( 38.4 ; C/C ) | 200 ( 47.4 ; C/T ) | 60 ( 14.2 ; T/T ) | 0.01249 |
|  | rs2119112 | 17 | G/A | 12 | 328 ( 77.7 ; G/G ) | 87 ( 20.6 ; G/A ) | 7 ( 1.7 ; A/A ) | 0.02026 |
|  | rs3731612 | 17 | G/A | 17.7 | 284 ( 67.3 ; G/G ) | 127 ( 30.1 ; G/A ) | 11 ( 2.6 ; A/A ) | 0.00000 |
|  | rs3806496 | 17 | G/A | 48.9 | 116 ( 27.5 ; G/G ) | 199 ( 47.2 ; A/G ) | 107 ( 25.4 ; A/A ) | 0.00075 |
|  | rs4491733 | 17 | A/G | 26.7 | 225 ( 53.3 ; A/A ) | 169 ( 40 ; A/G ) | 28 ( 6.6 ; G/G ) | 0.00000 |
|  | rs4849743 | 17 | T/C | 14.4 | 307 ( 72.9 ; T/T ) | 107 ( 25.4 ; T/C ) | 7 ( 1.7 ; C/C ) | 0.00000 |
|  | rs7599352 | 17 | T/C | 43.4 | 137 ( 32.5 ; T/T ) | 204 ( 48.3 ; C/T ) | 81 ( 19.2 ; C/C ) | 0.00000 |
| *MMP9* | rs17576 | 5 | A/G | 37.8 | 154 ( 36.5 ; A/A ) | 217 ( 51.4 ; A/G ) | 51 ( 12.1 ; G/G ) | 0.00003 |
|  | rs2274756 | 5 | G/A | 15.4 | 304 ( 72 ; G/G ) | 106 ( 25.1 ; G/A ) | 12 ( 2.8 ; A/A ) | 0.00000 |
|  | rs3918261 | 5 | A/G | 15.5 | 304 ( 72 ; A/A ) | 105 ( 24.9 ; A/G ) | 13 ( 3.1 ; G/G ) | 0.00000 |
|  | rs4810482 | 5 | T/C | 38.7 | 150 ( 35.5 ; T/T ) | 217 ( 51.4 ; T/C ) | 55 ( 13 ; C/C ) | 0.00003 |
| *MS4A7* | rs950802 | 3 | G/A | 39.8 | 154 ( 36.5 ; G/G ) | 200 ( 47.4 ; G/A ) | 68 ( 16.1 ; A/A ) | 0.02456 |
| *MT1E* | rs12934166 | 1 | G/T | 10.6 | 334 ( 79.5 ; G/G ) | 83 ( 19.8 ; G/T ) | 3 ( 0.7 ; T/T ) | 0.00075 |
| *NRIP1* | rs2178894 | 10 | C/T | 35.9 | 182 ( 43.1 ; C/C ) | 177 ( 41.9 ; T/C ) | 63 ( 14.9 ; T/T ) | 0.03874 |
| *PCK1* | rs6070157 | 10 | C/T | 20.5 | 268 ( 63.5 ; C/C ) | 135 ( 32 ; C/T ) | 19 ( 4.5 ; T/T ) | 0.03261 |
| *PCK2* | rs1951634 | 3 | G/T | 27.7 | 223 ( 53 ; G/G ) | 163 ( 38.7 ; T/G ) | 35 ( 8.3 ; T/T ) | 0.00000 |
|  | rs3561 | 3 | T/C | 36.5 | 169 ( 40 ; T/T ) | 198 ( 46.9 ; C/T ) | 55 ( 13 ; C/C ) | 0.00000 |
| *PECR* | rs17548270 | 10 | G/A | 14.8 | 308 ( 73 ; G/G ) | 103 ( 24.4 ; G/A ) | 11 ( 2.6 ; A/A ) | 0.02037 |
|  | rs2303842 | 10 | C/T | 23.6 | 247 ( 58.5 ; C/C ) | 151 ( 35.8 ; C/T ) | 24 ( 5.7 ; T/T ) | 0.02278 |
| *PHYH* | rs3802577 | 16 | T/C | 31.6 | 197 ( 46.7 ; T/T ) | 183 ( 43.4 ; T/C ) | 42 ( 10 ; C/C ) | 0.00247 |
| *SCARA5* | rs11779320 | 37 | C/T | 17.3 | 294 ( 69.7 ; C/C ) | 110 ( 26.1 ; C/T ) | 18 ( 4.3 ; T/T ) | 0.03261 |
|  | rs2726938 | 37 | T/G | 47 | 126 ( 29.9 ; T/T ) | 195 ( 46.2 ; G/T ) | 101 ( 23.9 ; G/G ) | 0.00689 |
|  | rs2726942 | 37 | C/T | 46.9 | 127 ( 30.1 ; C/C ) | 194 ( 46 ; T/C ) | 101 ( 23.9 ; T/T ) | 0.00689 |
|  | rs884829 | 37 | T/C | 25.9 | 229 ( 54.3 ; T/T ) | 167 ( 39.6 ; T/C ) | 26 ( 6.2 ; C/C ) | 0.01520 |
|  | rs939705 | 37 | A/C | 47 | 126 ( 29.9 ; A/A ) | 195 ( 46.2 ; C/A ) | 101 ( 23.9 ; C/C ) | 0.00689 |
| *TXNDC5* | rs11962800 | 41 | A/G | 10.1 | 341 ( 80.8 ; A/A ) | 77 ( 18.2 ; A/G ) | 4 ( 0.9 ; G/G ) | 0.00806 |
