# Supplementary material for: Determinants of Human Adipose Tissue Gene Expression: Impact of Diet, Sex, Metabolic Status, and Cis Genetic Regulation
Source: PLoS Genet. 2012 Sep 27;8(9):e1002959. doi: 10.1371/journal.pgen.1002959 (PMC3459935; doi:10.1371/journal.pgen.1002959)
Supplement: Table S13 — Significativity of SNP association with adipose tissue gene expression along the dietary program. To test the validity of the association of the SNPs significant at CID1 with gene expression along the dietary program, a linear mixed model was ran with gene expression data at CID1, CID2 and CID3 according to the regression equations displayed below. Each SNP was evaluated under a log additive model assuming a linear allele-dose effect (0 = Wild-type, 1 = heterozygote, 2 = homozygote). The regression equations were tested without then with BMI. Y is the log2 expression value for gene i, in subject l, and centre k. The random term ε represents the random error that was assumed to be normally distributed. The Benjamini-Hochberg procedure was used to control for multiple testing. CID1, CID2 and CID3 are, respectively, clinical investigation days at baseline, after the 8-week calorie restriction and after the 26-week weight maintenance diet. (DOCX) [file pgen.1002959.s018.docx]

**Table S13. Significativity of SNP association with adipose tissue gene expression along the dietary program**

| Gene | SNP | CID1 and CID2* | CID1, CID2 and CID3** | CID1 and CID3*** |
| --- | --- | --- | --- | --- |
| *ACSL1* | rs3792311 | 0.15297 | 0.23540 | 0.13153 |
| *ADH1C* | rs1614972 | 0.00004 | 0.03607 | 0.03487 |
|  | rs2851300 | 0.00000 | 0.00890 | 0.05179 |
|  | rs1789924 | 0.00000 | 0.00890 | 0.05179 |
|  | rs698 | 0.00001 | 0.01456 | 0.07252 |
|  | rs1662037 | 0.00468 | 0.05017 | 0.05734 |
|  | rs1614377 | 0.00559 | 0.05241 | 0.06389 |
|  | rs9307239 | 0.10753 | 0.27587 | 0.10240 |
| *ALDOB* | rs479600 | 0.00000 | 0.00000 | 0.00000 |
|  | rs533017 | 0.00596 | 0.00948 | 0.00348 |
|  | rs550915 | 0.00689 | 0.04254 | 0.03834 |
| *ANG* | rs1010458 | 0.18676 | 0.69575 | 0.70820 |
| *AP2M1* | rs843346 | 0.01480 | 0.26585 | 0.25068 |
| *ATP8A1* | rs6851867 | 0.07911 | 0.00000 | 0.00000 |
|  | rs4550967 | 0.17810 | 0.00001 | 0.00002 |
| *CARHSP1* | rs4985053 | 0.00216 | 0.24239 | 0.32365 |
|  | rs10163323 | 0.00132 | 0.04816 | 0.14238 |
| *CCL19* | rs10972195 | 0.00941 | 0.06786 | 0.05790 |
| *CCL3* | rs1634508 | 0.00001 | 0.00680 | 0.00886 |
|  | rs9972960 | 0.00014 | 0.19210 | 0.39976 |
|  | rs1851503 | 0.00089 | 0.05241 | 0.15621 |
|  | rs1719220 | 0.00154 | 0.02634 | 0.05519 |
| *CD14* | rs7721577 | 0.00322 | 0.00051 | 0.00148 |
|  | rs3822356 | 0.00415 | 0.00062 | 0.00173 |
|  | rs2569193 | 0.00121 | 0.00052 | 0.00291 |
|  | rs12517200 | 0.00206 | 0.00081 | 0.00347 |
| *CD48* | rs1126644 | 0.01480 | 0.15183 | 0.40617 |
| *CDK2AP1* | rs10846489 | 0.00177 | 0.00004 | 0.00001 |
| *CIDEA* | rs12966216 | 0.00000 | 0.00007 | 0.00012 |
|  | rs7504200 | 0.00001 | 0.00358 | 0.00795 |
|  | rs7230480 | 0.01994 | 0.04254 | 0.05734 |
|  | rs10853223 | 0.02546 | 0.27992 | 0.40629 |
| *CSTB* | rs3761385 | 0.02132 | 0.27992 | 0.12792 |
|  | rs2838363 | 0.00775 | 0.10074 | 0.09027 |
| *CTSZ* | rs163801 | 0.00017 | 0.00135 | 0.01060 |
| *CYYR1* | rs1395053 | 0.04676 | 0.03221 | 0.03469 |
|  | rs222956 | 0.22283 | 0.05148 | 0.01069 |
|  | rs219672 | 0.00668 | 0.07324 | 0.19875 |
| *ECHDC3* | rs2055040 | 0.01391 | 0.06735 | 0.04332 |
| *EHD4* | rs10518743 | 0.00006 | 0.00009 | 0.00001 |
|  | rs16972308 | 0.00011 | 0.00013 | 0.00001 |
|  | rs17739167 | 0.15840 | 0.05081 | 0.02899 |
| *EN2* | rs2885339 | 0.01129 | 0.00163 | 0.00023 |
| *FADS1* | rs174556 | 0.00178 | 0.00048 | 0.00300 |
|  | rs174546 | 0.00309 | 0.00038 | 0.00399 |
| *FBP1* | rs4129219 | 0.00509 | 0.64215 | 0.37440 |
| *FCER1G* | rs4489574 | 0.02001 | 0.06776 | 0.13871 |
|  | rs2070902 | 0.03059 | 0.01864 | 0.02977 |
|  | rs4233368 | 0.02862 | 0.02634 | 0.05734 |
| *GATM* | rs4774580 | 0.00000 | 0.00000 | 0.00000 |
|  | rs1346268 | 0.00000 | 0.00000 | 0.00000 |
|  | rs2461700 | 0.00006 | 0.00014 | 0.00012 |
| *HLA-A* | rs16896742 | 0.00000 | 0.00000 | 0.00000 |
|  | rs2860580 | 0.00000 | 0.00001 | 0.00002 |
| *HP* | rs1424241 | 0.00000 | 0.00180 | 0.02289 |
| *HSDL2* | rs10817330 | 0.00344 | 0.00163 | 0.00190 |
|  | rs10817344 | 0.00322 | 0.00762 | 0.00886 |
|  | rs4978489 | 0.01047 | 0.05017 | 0.06711 |
|  | rs4979108 | 0.00216 | 0.00358 | 0.01672 |
|  | rs16916995 | 0.00795 | 0.06023 | 0.09027 |
| *IL1RN* | rs315948 | 0.03123 | 0.61978 | 0.64154 |
|  | rs315952 | 0.02463 | 0.58168 | 0.60469 |
| *IRF5* | rs4728142 | 0.04654 | 0.02866 | 0.03189 |
| *ITGB5* | rs1007856 | 0.00132 | 0.01395 | 0.00875 |
|  | rs9968182 | 0.00135 | 0.01586 | 0.00974 |
|  | rs4141663 | 0.00132 | 0.01136 | 0.00621 |
|  | rs11928651 | 0.00092 | 0.01456 | 0.01264 |
|  | rs16836080 | 0.01177 | 0.02634 | 0.04173 |
| *KLB* | rs900563 | 0.00268 | 0.09912 | 0.10240 |
|  | rs2381378 | 0.00415 | 0.11567 | 0.11728 |
| *LILRA6* | rs4575638 | 0.00023 | 0.00706 | 0.05087 |
| *LILRB3* | rs13353326 | 0.00000 | 0.00000 | 0.00000 |
|  | rs3865475 | 0.00000 | 0.00000 | 0.00029 |
| *LIPA* | rs2243548 | 0.04749 | 0.05065 | 0.05734 |
|  | rs2250645 | 0.01002 | 0.01743 | 0.05306 |
|  | rs2243547 | 0.00957 | 0.02634 | 0.13855 |
|  | rs1051338 | 0.01218 | 0.02123 | 0.07706 |
| *LOX* | rs2731647 | 0.00415 | 0.00576 | 0.00875 |
|  | rs3853401 | 0.00575 | 0.01743 | 0.03581 |
| *LY86* | rs977785 | 0.00000 | 0.00003 | 0.00022 |
|  | rs6597220 | 0.00000 | 0.00003 | 0.00025 |
|  | rs9405942 | 0.00000 | 0.00003 | 0.00043 |
|  | rs9328374 | 0.00002 | 0.01117 | 0.02337 |
|  | rs1999652 | 0.00006 | 0.00627 | 0.00886 |
|  | rs3804474 | 0.05541 | 0.03698 | 0.00816 |
|  | rs9328376 | 0.05072 | 0.00851 | 0.00399 |
|  | rs1905045 | 0.00945 | 0.02123 | 0.01774 |
| *MARCO* | rs3731612 | 0.00000 | 0.00000 | 0.00000 |
|  | rs4491733 | 0.00000 | 0.00000 | 0.00000 |
|  | rs17796260 | 0.00000 | 0.00000 | 0.00000 |
|  | rs4849743 | 0.00000 | 0.00000 | 0.00000 |
|  | rs7599352 | 0.00000 | 0.00043 | 0.00022 |
|  | rs11693199 | 0.00089 | 0.02634 | 0.04173 |
|  | rs12997897 | 0.00026 | 0.01031 | 0.06948 |
|  | rs3806496 | 0.00208 | 0.15314 | 0.09348 |
|  | rs1371562 | 0.00206 | 0.02634 | 0.04332 |
|  | rs2011839 | 0.02507 | 0.15317 | 0.18279 |
|  | rs2119112 | 0.05072 | 0.02634 | 0.02087 |
|  | rs17009716 | 0.05072 | 0.02634 | 0.02087 |
| *MMP9* | rs3918261 | 0.00000 | 0.00000 | 0.00000 |
|  | rs2274756 | 0.00000 | 0.00000 | 0.00000 |
|  | rs17576 | 0.00001 | 0.00013 | 0.00085 |
|  | rs4810482 | 0.00001 | 0.00006 | 0.00025 |
| *MS4A7* | rs950802 | 0.00157 | 0.04211 | 0.04423 |
| *MT1E* | rs12934166 | 0.00358 | 0.01796 | 0.00291 |
| *NRIP1* | rs2178894 | 0.02663 | 0.01326 | 0.00399 |
| *PCK1* | rs6070157 | 0.00199 | 0.24356 | 0.36076 |
| *PCK2* | rs1951634 | 0.00008 | 0.00051 | 0.00025 |
|  | rs3561 | 0.00006 | 0.00009 | 0.00002 |
| *PECR* | rs17548270 | 0.04097 | 0.90246 | 0.83432 |
|  | rs2303842 | 0.01480 | 0.23176 | 0.20045 |
| *PHYH* | rs3802577 | 0.00084 | 0.01139 | 0.00166 |
| *SCARA5* | rs2726942 | 0.00291 | 0.04609 | 0.02289 |
|  | rs939705 | 0.00260 | 0.04609 | 0.02289 |
|  | rs2726938 | 0.00260 | 0.04609 | 0.02289 |
|  | rs884829 | 0.00178 | 0.00294 | 0.00304 |
|  | rs11779320 | 0.01129 | 0.28632 | 0.20687 |
| *TXNDC5* | rs11962800 | 0.01609 | 0.10571 | 0.10765 |
